# Layer-by-Layer Proteomic Analysis of *Mytilus galloprovincialis* Shell

**DOI:** 10.1371/journal.pone.0133913

**Published:** 2015-07-28

**Authors:** Peng Gao, Zhi Liao, Xin-xing Wang, Lin-fei Bao, Mei-hua Fan, Xiao-min Li, Chang-wen Wu, Shu-wei Xia

**Affiliations:** 1 College of Chemistry and Chemical Engineering, Ocean University of Chinese, Qingdao, China; 2 Laboratory of Marine Biological Protein Engineering, Zhejiang Ocean University, Zhoushan, Zhejiang, China; 3 Biotechnology Center, Chinese Academy of Fishery Science, Beijing, China; University of Connecticut, UNITED STATES

## Abstract

Bivalve shell is a biomineralized tissue with various layers/microstructures and excellent mechanical properties. Shell matrix proteins (SMPs) pervade and envelop the mineral crystals and play essential roles in biomineralization. Despite that *Mytilus* is an economically important bivalve, only few proteomic studies have been performed for the shell, and current knowledge of the SMP set responsible for different shell layers of *Mytilus* remains largely patchy. In this study, we observed that *Mytilus galloprovincialis* shell contained three layers, including nacre, fibrous prism, and myostracum that is involved in shell-muscle attachment. A parallel proteomic analysis was performed for these three layers. By combining LC-MS/MS analysis with *Mytilus* EST database interrogations, a whole set of 113 proteins was identified, and the distribution of these proteins in different shell layers followed a mosaic pattern. For each layer, about a half of identified proteins are unique and the others are shared by two or all of three layers. This is the first description of the protein set exclusive to nacre, myostracum, and fibrous prism in *Mytilus* shell. Moreover, most of identified proteins in the present study are novel SMPs, which greatly extended biomineralization-related protein data of *Mytilus*. These results are useful, on one hand, for understanding the roles of SMPs in the deposition of different shell layers. On the other hand, the identified protein set of myostracum provides candidates for further exploring the mechanism of adductor muscle-shell attachment.

## Introduction

Mollusk shell, primarily consisting of calcium carbonate crystals together with organic matrix, has been investigated as a typical biomineralization model for dozens years [1–4]. It is well known that there are three major calcium carbonate minerals (calcite, aragonite, and vaterite) with the same principal composition, despite the different structures [5]. Of these minerals, the two most thermodynamically stable structures, calcite and aragonite, are deposited extensively as biominerals. In general, most of the adult shell of bivalves found in nature are composed of calcite and/or aragonite with different shapes and morphologies, including ‘‘nacreous”, ‘‘prismatic”, ‘‘foliated”, ‘‘cross-lamellar”, ‘‘granular”, ‘‘composite-prismatic”, and ‘‘homogeneous” structures [6, 7]. Among the most studied of them are nacre and prism. Nacre is a widespread mollusk shell texture. It is represented within the three main classes of mollusks, Bivalves, Gastropods, and Cephalopods. The “nacre” terminology refers to a well-defined type of microstructure characterized by small flat tablets of aragonite tightly packed together by organic cement [8, 9]. Prism is usually calcite needles of various lengths and diameters. The thin oblique calcite prism named “fibrous prism” had been detected in *Mytilus edulis* shell [10]. Myostracum is usually a very thin layer located in the attachment of the adductor muscle, commonly called the muscle scar or imprint, to the umbo of each valve. The adductor muscle scar, where the adductor muscle functions to close the shell, is the most conspicuous area on bivalve shell [11, 12].

Shell matrix proteins (SMPs) embedded within various calcified layers of mollusk shell were supposed to play an essential role in increasing shell mechanical properties and controlling the biomineral synthesis, such as crystal nucleation, crystal orientation, crystal size regulation, and crystal polymorphism [13,14]. Thus, characterization of the SMPs offers an opportunity for understanding the mechanism of biomineralization. However, most of SMPs are insoluble and cross-linked in the shell [15]. Difficulties are therefore encountered in extraction and purification strategies for protein characterization. To solve this problem, previously established methods to identify SMPs, such as individually biochemical characterization or molecular biology approaches, have been recently complemented by the use of mass spectrometry-based proteomics analysis or a combination of proteomics and transcriptomic studies [16–18]. However, most of identified SMPs so far are from *Pinctada* and *Haliotis*. The studies of *Mytilus* SMPs are very few in number. Additionally, considering the diversity of shell microstructures, one can speculate that different shell layers may consist of different protein assemblages. Earlier works had preliminarily confirmed the differences in the amino acid composition associated with nacre and prism [19]. While for *Mytilus*, the full picture of the protein repertoire associated with different shell layers is still absent.

*Mytilus galloprovincialis* is an economically important species and the shell consists of three layers, the inner layer of aragonite nacre, the outer layer of calcite fibrous prism, and the myostracum that mostly buried in nacre layer but exposed at the adductor muscle scar where the posterior adductor muscle attached to. The ready source of specimens and the nacro-prismatic shell microstructures make *M*. *galloprovincialis* an ideal model for studying the proteome of each shell layer. In the present study, we collected the nacre, myostracum, and fibrous prism from *M*. *galloprovincialis* shell separately and the shell matrix was extracted successively by cold acetic acid (designated as ASM) and 8M urea (designated as USM). Both of ASM and USM were further fractioned by HPLC and analyzed by LC-MS/MS for protein identification. For the resulting insoluble matrix, an LTQ-Orbitrap mass spectrometer was used for protein identification. By interrogating the MS/MS data against EST dataset of three closely related species of *Mytilus* (*M*. *edulis*, *M*. *galloprovincialis* and *M*. *californianus*), we report here for the first time the proteome of different shell layers and novel SMPs associated with nacre, myostracum, and fibrous prism in *Mytilus*.

## Materials and Methods

### Ethics Statement

Mussels were handled in accordance with the guidelines on the care and use of animals for scientific purposes set by the Institutional Animal Care and Use Committee (IACUC) of Zhejiang Ocean University, Zhoushan, China.

### Sample preparation

*M*. *galloprovincialis* was purchased from The Mussel Farm of Gouqi Island (30°42′48″N 122°46′42″E) of Zhejiang Province, China. The adult mussels (two-year old and 6~7 cm in shell length) were cut open using a razor blade and the soft parts were removed. The shells were freshly collected and the superficial organic contaminants (including adductor muscle) were removed by incubating each intact shell in 200 mL NaOH (5%, v/v) for 1 h at 25°C. The shells were then washed with de-ionized water six times and dried. Powdered samples were collected using a scalpel from shell interior surface at the regions corresponding to nacre, myostracum, and fibrous prism, respectively.

### SEM

The shell was fractured at the region around adductor muscle scar and the prepared samples were sputter-coated with gold. The section of shell samples were examined with a VEGA-3 TCSCANER scanning electron microscope at 20 kV accelerator voltage. Shell layers were identified by the sharp contact between the two types of shell microstructures, and by the change in their mineralogy.

### Protein extraction and purification

Step 1, the powdered samples (50mg) from each shell layer were suspended in 100mL acetic acid (5%, *v/v*) for 12 h at 4°C with continuous stirring. The suspension was then centrifuged (10,000×g, 20 min, 4°C). The supernatant comprising the acid-soluble matrix (ASM) was filtered (0.22 μm) and dialyzed against de-ionized water before being freeze-dried and weighed.

Step 2, the acid-insoluble matrix was recovered in 5% acetic acid (*v/v*,) containing 8M urea and 0.1 mM tri (carboxyethyl)-phosphine. After homogenization at 4°C using a small hand-held tissue grinder., the homogenate was centrifuged (12,000 ×g, 20 min, 4°C) and the supernatant was dialyzed against 4 liters of 5% acetic acid twice. The supernatant, corresponding to the urea-soluble matrix (USM), was then freeze-dried and weighed.

Step 3, after extracted by acetic acid and urea, the resulting pellets, corresponding to insoluble matrix, was rinsed six times with de-ionized water, freeze-dried and weighed.

HPLC purification was carried out on a Waters Delta 600 HPLC system equipped with a Waters 2487 absorbance detector (Waters USA). About 1 mg of ASM and USM from each of the shell layers were subjected to a Sunfire analytic C8 column (4.6×250 mm, Waters) that equilibrated with deionized water containing 0.1% trifluoroacetic acid (TFA). Gradient of 5–20% acetonitrile containing 0.1% TFA over 5 min, followed by 20–60% gradient over 60 min and 60–95% gradient over 5 min were performed at a flow rate of 1.0 mL/min. The eluted fractions (monitored at 280 nm) were collected manually and freeze-dried for LC-MS/MS analysis.

SDS-PAGE was done on a Mini-PROTEAN Tetra Electrophoresis System (8.3×7.3cm, Bio-Rad) with a 4% stacking gel and 13% resolving gel. Samples (100μg) of ASM, USM, and insoluble matrices were suspended in 20 μL loading buffer and heated to 95°C for 5 min. Loading buffer-insoluble material was removed by centrifugation in an Eppendorf bench top centrifuge for 5 min at 13000 rpm. Gels were loaded with 15 μL of matrix sample supernatant per lane and stained with Coomassie Brilliant Blue R250 after electrophoresis.

### MS analysis

Before mass spectrometry (MS) analysis, the protein sample (about 100 μg) was reduced with 10 mM dithiothreitol in 50 mM NH_4_HCO_3_ at 57°C for 1 h and alkylated by 50 mM iodoacetamide in 50 mM NH_4_HCO_3_ for 45 min at room temperature in the dark. After treated with 2 μg of trypsin in 10 μL 50 mM NH_4_HCO_3_ for 18 h at 37°C, the sample was dried in a vacuum concentrator and re-suspended in 30 μL of 0.1% (TFA) and 4% acetonitrile.

The LC-MS/MS analysis was performed using a QSTAR-ELite system (Applied Biosystems, USA) interfaced with a 20 AD HPLC system (Shimadzu, Japanese). The trypsin digested mixture of protein sample (about 20 μg) was separated on a Zorbax 300SB-C18 column (0.1×150 mm, 5μ, 300 Å; Microm, Auburn, CA). The HPLC gradient was 5~35% buffer B (95% ACN, 0.1% formic acid) in buffer A (5% ACN, 0.1% formic acid) at a flow rate of 0.3 mL/min over 90 min. MS data were acquired automatically using Analyst QS 1.1 software (Applied Biosystems). Following a MS survey scan over m/z 350~1 500, the MS/MS spectra were sequentially and dynamically acquired for the eight most intense peptide molecular ions over m/z 100~2 000.

The LTQ analysis of insoluble fraction was performed on a LC-20AD system (Shimadzu, Tokyo, Japan) connected to an LTQ Orbitrap mass spectrometer (Thermo Electron, Bremen, Germany) equipped with an online nano-electrospray ion source (Michrom Bioresources, Auburn, CA). The trypsin digested mixture of insoluble fraction was separated on a reverse phase column (100 μ, MICHROM Bioresources Inc., Auburn, CA). The HPLC gradient was 5–45% of solvent B (95% ACN containing 0.1% formic acid) over 90 min at a flow rate of 60 μL/min. Eluted peptides were analyzed by MS and data-dependent MS/MS acquisition, selecting the 8 most abundant precursor ions for MS/MS with a dynamic exclusion duration of 1 min.

### Data analysis

Protein identification was performed using the MASCOT search engine (version 2.1, Matrix Science, London, UK) against a protein database comprising 68209 sequences derived from the EST libraries of *Mytilus spp*. (Taxid: 6548, mainly represented by 42354 sequences from *M*. *californianus*, 19617 from *M*. *galloprovincialis*, 5300 from *M*. *edulis*, and 719 from *M*. *coruscus*) downloaded (August, 2014) from the NCBI server (http://www.ncbi.nlm.nih.gov). Methionine oxidation and carbamidomethylation were set as variable and fixed modifications, respectively. The mass tolerance was set to 20 ppm for the precursor and 0.3 Da for the product ions. The peptide hits were manually confirmed by the inspection of the MS/MS spectra. Ions scores are defined as (-10*Log(p)), where p is the probability that the observed match is a random event. Only peptide matches with an expectation score ≤0.05 were accepted.

Sequence analysis was performed using Lasergene software (version 7.1). Sequence homologies were determined by searching non-redundant protein databases *via* the BLAST server (http://www.ncbi.nlm.nih.gov/BLAST/). Signal peptides were predicted using the SignalP 4.0 online tool (http://www.cbs.dtu.dk/services/SignalP/) and conserved domains were predicted using SMART online tool at http://smart.embl-heidelberg.de/. The amino acid composition was calculated by ProtParam tool at http://web.expasy.org/protparam/. Sequence alignments were performed with ClustalW2 at http://www.ebi.ac.uk/Tools/msa/clustalw2/.

## Results

### The microstructure of *M*. *galloprovincialis* shell

**Fig 1:**
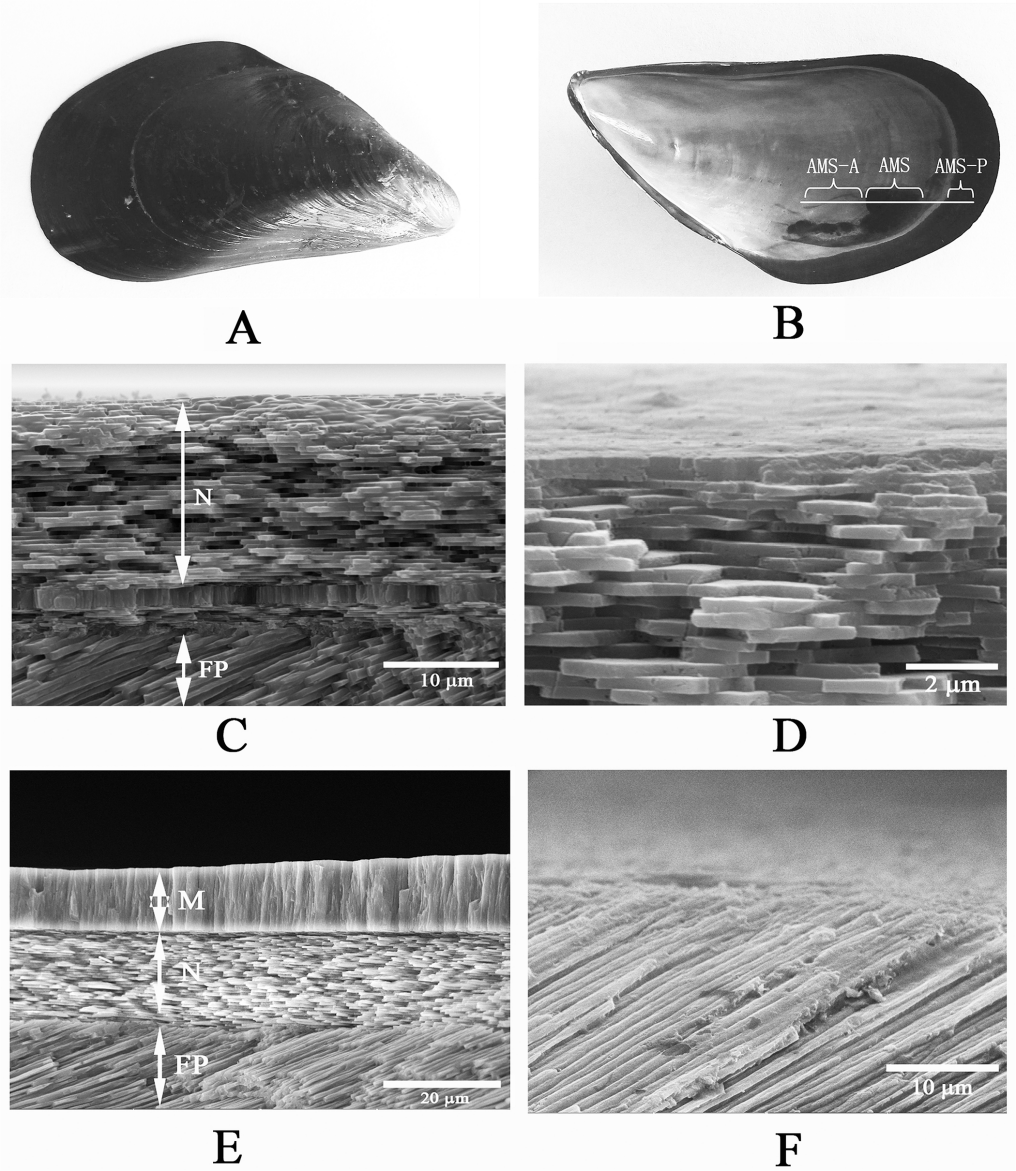
The photograph of the whole shell and the SEM images of cross-section of *M*. *galloprovincialis* shell. A: the exterior side of *M*. *galloprovincialis* shell; B: the interior side of *M*. *galloprovincialis* shell, the white line represents the cutting plane; “AMS”, “AMS-A”, and “AMS-P” represent the area of adductor muscle scar (AMS), anterior side of AMS, and posterior side of AMS, respectively. C: the cross-sectional SEM image of AMS-A, showed nacre (N) on the top layer and fibrous prism (FP) at the bottom of this area; D: enlarged image of nacre from AMS-A; E: the cross-sectional SEM image of central AMS, showed myostracum (M) at the top, nacre (N) at the middle, and fibrous prism (FP) at the bottom of this area; F: the cross-sectional SEM image of AMS-P, showed fibrous prism at this area.

As shown in Fig 1, on the interior surface of adult *M*. *galloprovincialis* shell, three regions with different color and texture can be observed optically, including the adductor muscle scar (AMS), white color region (designated as AMS-A, anterior side of AMS), a black region located on the growing margin of the shell (designated as AMS-P, posterior side of AMS). The SEM images of the section of AMS, AMS-A, and AMS-P were shown in the Fig 1C–1F. Three layers, nacre, myostracum, and fibrous prism, were observed by SEM with different morphology. The top layer of ASM-A, AMS, and AMS-P, corresponding to the nacre, myostracum, and fibrous prism, respectively, was scraped off and the shell matrices were then extracted for proteomic analysis.

### Isolation of shell matrices from different shell layers

The amount of total organic matrix from shell layers represents an average of 3.4% by weight of the shell powder (wt/wt), and the highest content of organic material was presented in nacre (5.3%), followed by myostracum (3.5%) and fibrous prism (1.9%). The amount of ASM was 1.2%, 0.7%, and 0.5% for the nacre, myostracum and fibrous prism, respectively. Further extracted USM yielded additional 1.0%, 0.6%, and 0.8% for the nacre, myostracum and fibrous prism, respectively. The soluble matrix (ASM plus USM), formed more than 40% of the total organic matrix in each shell layer, was further fractioned by HPLC. The insoluble fractions were directly analyzed by LTQ.

**Fig 2:**
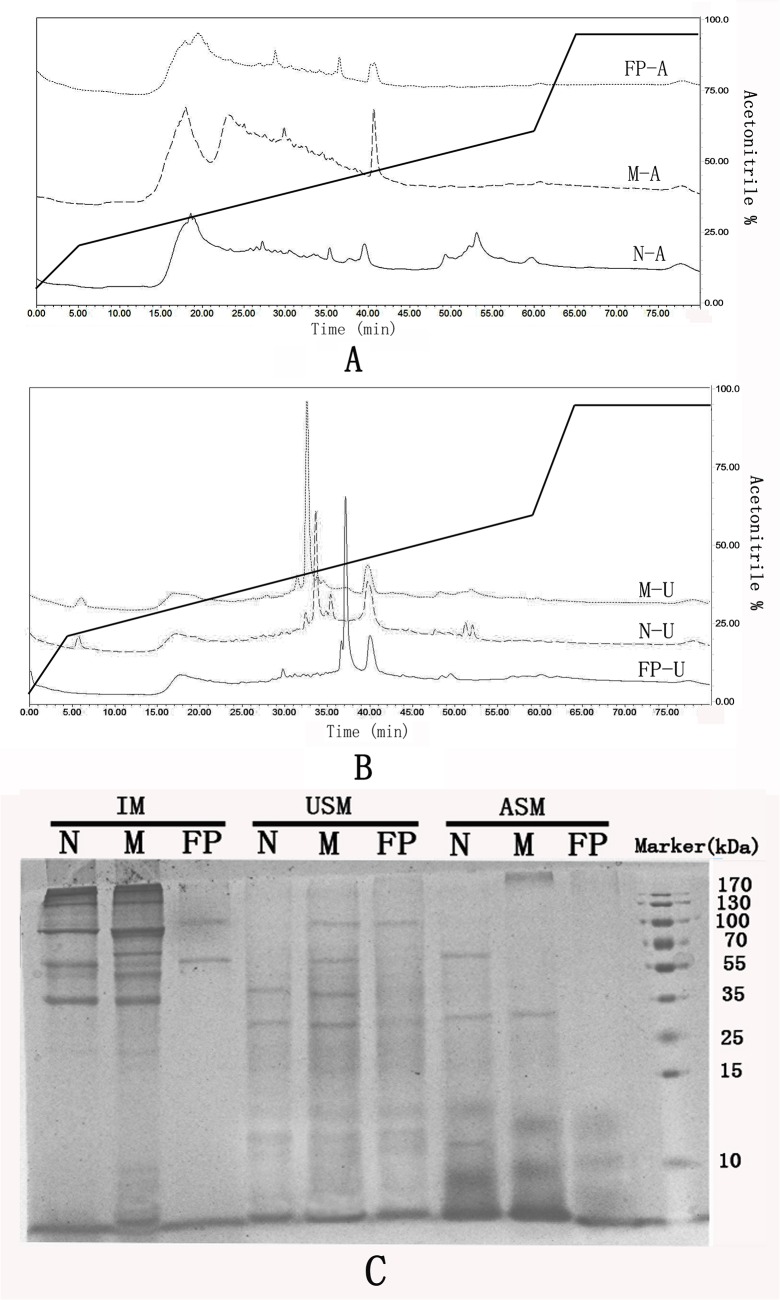
HPLC elution profiles and SDS-PAGE analysis of shell matrices from nacre, myostracum, and fibrous prism, respectively. A: HPLC elution profiles of acid-soluble matrices from nacre (N-A), myostracum (M-A), and fibrous prism (FP-A). The bold line represents the concentration of acetonitrile. B: HPLC elution profiles of urea-soluble matrices from nacre (N-U), myostracum (M-U), and fibrous prism (FP-U). The bold line represents the concentration of acetonitrile. C: PAGE comparison of acid-soluble (ASM), urea-soluble (USM), and insoluble matrices (IM) of nacre (N), myostracum (M), and fibrous prism (FP).

HPLC purification of ASMs from three shell layers showed a similar profile with minor differences (Fig 2). The chromatogram obtained on a C8 column for ASMs showed a bell-shaped elution profile at the region of 25–35% acetonitrile and several peaks at the region of 35–45% acetonitrile, indicating the presence of multiple proteins with similar chromatographic properties. For nacre, an additional fraction was eluted at the region of 45–55% acetonitrile, indicating more hydrophobic proteins of nacre than that of myostracum and fibrous prism. The HPLC chromatogram of USMs showed a better elution profile with several sharp peaks presented in the region of 40–45% of acetonitrile and these individual peaks were observed with different retention time for USMs of three layers. In order to investigate the largest part of *M*. *galloprovincialis* shell proteome, the HPLC eluted fractions were collected according to the order of the eluted peaks (denoted by “I”, “II”, “III”, etc. in S1 Fig) and analyzed by LC–MS/MS.

Comparison of the protein band pattern of matrices from different shell layers showed comparable differences (Fig 2C). More protein bands were observed in insoluble matrices than that in soluble matrix (ASM and USM). Further, the samples of nacre and myostracum apparently showed more complexity of protein composition than that of fibrous prism. These results suggest that the proteome variables were not only in different shell layers but also in different extraction protocols.

### Protein identification of soluble matrices from different shell layers

**Fig 3:**
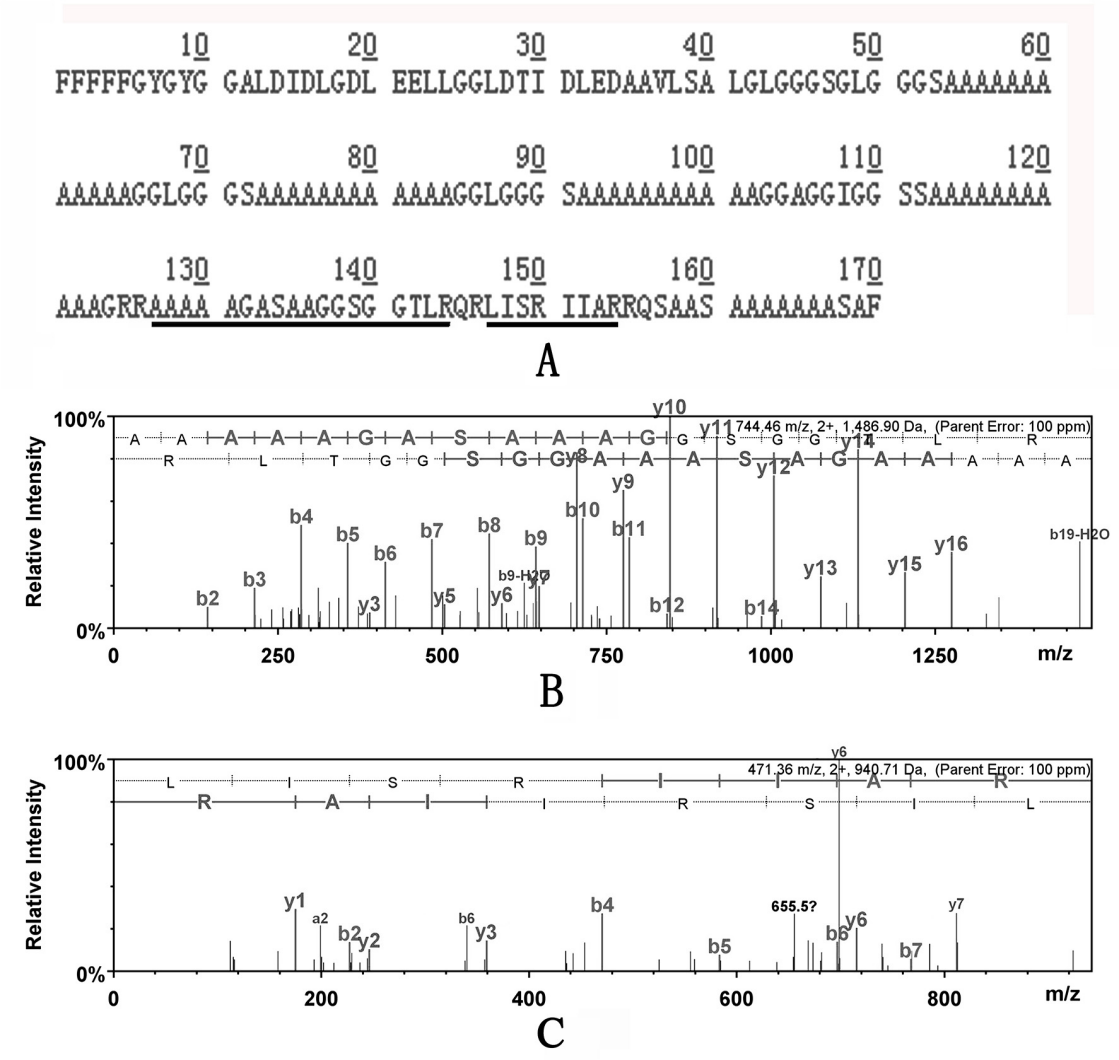
A representative MS/MS spectrum for the two peptides matching with EST gi|58308563, an AGL-rich protein of *M*. *galloprovincialis*. A: protein sequence derived from EST gi|58308563. The matched peptides from MS/MS spectra were underlined; B: MS/MS spectrum of the peptide “-AAAAAGASAAAGGSGGTLR-” with *m/z* of 1486.90 Da; C: MS/MS spectrum of the peptide “-LISRIIAR-” with *m/z* of 940.71 Da.

By combining the LC-MS/MS analysis with *Mytilus* EST database interrogations, a set of 18 proteins were identified from the ASMs of three layers, including fifteen, seven, and five proteins yielded by nacre, myostracum and fibrous prism, respectively (Table 1 and S1 Table). We observed some proteins with high mascot score and high sequence identity (more than 80%) with previously reported *Mytilus* SMPs, including MUSPs, Shell Matrix Protein, Perlucin-like protein, MSI60-like protein, Fibronectin-like protein, and EP-protein [20, 21], In addition, many novel shell proteins were identified from the three layers. From nacre, a protein containing chitin-binding domain was identified with low sequence identity (34%) to BMSP (blue mussel shell protein), a calcium carbonate-binding protein from *M*. *galloprovincialis* shell [22]. EST gi|154348940 was identified as a 216-AA long protein with a 16-AA long signal peptide and showed 71% sequence identity with Apextrin-like protein of *M*. *galloprovincialis*, a potential immune-related protein [23]. Five proteins showed neither homology nor putative domains, but with unusual amino acid composition. For example, EST gi|58308563, the most abundant protein in the ASM, encodes an AGL-rich protein containing abundant Ala (41.5%), Gly (21.6%), and Leu (8.8%), and several poly-Ala blocks (consist of 11–12 alanines per block) (Fig 3 and S1 Table). The other novel proteins were AGS-rich protein (43.7% of Ala, 22.8% of Gly, and 10.2% of Ser), GRS-rich protein (22.8% of Gly, 9.5% of Arg, and 8.2% of Ser), and GA-rich protein (33.4% of Gly and 30.8% of Ala). For myostracum, three novel proteins were identified. Including collagen-like protein, protein with THY domains (thymosin beta actin-binding motif), and AGL-rich protein. For the fibrous prism, only five proteins were identified, including MSI60-like protein.

**Table 1:** The proteome of nacre (N), myostracum (M), and fibrous prism (M) of *M*. *galloprovincialis* shell.

Layer	Fractions	Protein (matched EST)	Homologue / Organism	Domains (ID) or features
N	A	GRS-rich protein (gi|223026932)	—	GRS-rich
N	A	AGS-rich protein (gi|212816250)	—	AGS-rich
N	A	apextrin-like protein (gi|154348940)	apextrin-like protein / M. galloprovincialis	internal repeats
N	A	BMSP-like protein (gi|212814599)	BMSP / M. galloprovincialis	Chitin-binding
N	A	GA-rich protein(gi|237638533)	—	GA-rich
N	A	GRS-rich protein-1 (gi|223026932)	—	GRS-rich
N	A	GRS-rich protein-2 (gi|212816291)	—	GRS-rich
M	A	THY domains containing protein (gi|238649869)	—	THY
FP	A	MSI60-like protein (gi|212814500)	MSI60-like protein / M. californianus	—
N+M+FP	A+I	collagen-like protein-3 (gi|58307858)	distal byssal thread collagen / synthetic construct	Collagen
N+M+FP	A+I	Shell matrix protein-1 (gi|145887968)	Shell matrix protein / M. californianus	Laminin_G_3
N+FP	A+U	EP protein-1 (gi|145893971)	EP protein / M. edulis	C1Q
N+FP	A+U	Fibronectin-like protein-1 (gi|212815602)	Fibronectin-like protein / M. californianus	FN3
N+M+FP	A+U+I	AGL-rich protein (gi 58308563)	—	AGL-rich
N+M+FP	A+U+I	MUSP-3 (gi|212814580)	MUSP-3 / M. californianus	—
N+M+FP	A+U+I	Perlucin-like protein (gi|58306883)	Perlucin-like protein / M. galloprovincialis	CLECT
N+M	A+U+I	Shell matrix protein-2 (gi|145887813)	Shell matrix protein / M. californianus	—
N	A+U+I	MUSP-1 (gi|223021659)	MUSP-1 / M. galloprovincialis	—
N+M+FP	I	calponin-like proteins-1 (gi|212823360)	calponin-like protein / M.galloprovincialis	Calponin
N+M+FP	I	calponin-like proteins-2 (gi|223023515)	calponin-like protein / M.galloprovincialis	Calponin
N+M+FP	I	calponin-like proteins-3 (gi|223026184)	calponin-like protein / M.galloprovincialis	Calponin
N+M+FP	I	collagen-like protein-2 (gi|58307336)	precollagen-D / M. galloprovincialis	Collagen
N+M+FP	I	filament-like protein-1 (gi|37650124)	60 kDa neurofilament protein / C. gigas	Filament
N+M+FP	I	GASD-rich protein (gi 212816630)	—	GASD-rich
N+M+FP	I	MGK-rich protein-1 (gi|223021924)	—	MGK-rich
N+M+FP	I	RKS-rich protein (gi|145893257)	—	RKS-rich
N+M	I	arginine kinase-like protein-1 (gi|164595782)	arginine kinase / C. novaehollandiae	ATP-gua_Ptrans
N+M	I	calponin-like proteins-4 (gi|145896743)	calponin-like protein / M.galloprovincialis	Calponin
N+M	I	calponin-like proteins-5 (gi|58308196)	calponin-like protein / M.galloprovincialis	Calponin
N+M	I	LGDK-rich protein (gi|212830099)	—	LGDK-rich
N+M	I	SR-rich protein (gi|58306383)	—	SR-rich
N+FP	I	calponin-like proteins-6 (gi|212814945)	calponin-like protein / M.galloprovincialis	Calponin
N+FP	I	calponin-like proteins-7 (gi|223026183)	calponin-like protein / M.galloprovincialis	Calponin
N+FP	I	Cathepsin-like protein-1 (gi|238641522)	Cathepsin L / C. gigas	Inhibitor_I29; Pept_C1
N+FP	I	monocarboxylate transporter-like protein (gi|212821329)	monocarboxylate transporter 1 / C. gigas	—
N+FP	I	Perlucin-like protein-2 (gi|238644365)	Perlucin-like protein / M. galloprovincialis	CLECT
N+FP	I	RKA-rich protein (gi 223022517)	—	RKA-rich
N	I	C1Q domain containning protein-1 (gi|238643096)	MgC1Q / M. galloprovincialis	C1q
N	I	calponin-like proteins-8 (gi|223026111)	calponin-like protein / M.galloprovincialis	Calponin
N	I	chitinase-like protein-1 (gi|58307961)	acidic mammalian chitinase/ S. boliviensis.	Glyco_18
N	I	chitinase-like protein-2 (gi|58308904)	acidic mammalian chitinase-like / P. sinensis	Glyco_18
N	I	chitinase-like protein-3 (gi|58308122)	chitotriosidase-1 / U.maritimus	Glyco_18
N	I	chitinase-like protein-4 (gi|380851837)	chitotriosidase-1 / U.maritimus	Glyco_hydro_18
N	I	chitinase-like protein-5 (gi|58306567)	chitotriosidase-1-like / M. davidii	—
N	I	collagen-like protein-4 (gi|58306536)	collagen alpha-1(XII) chain-like / A. californica	VWA
N	I	collagen-like protein-5 (gi|58308539)	collagen alpha-4(VI) chain-like / A. californica	VWA
N	I	dioxygenase-like protein (gi|212819041)	procollagen-proline dioxygenase/ M. galloprovincialis	Thioredoxin_6
N	I	EP protein-2 (gi|238643094)	EP protein / M. edulis	C1q
N	I	Fibronectin-like protein-2 (gi|212831833)	Fibronectin-like protein / M. californianus	FN3
N	I	filament-like protein-2 (gi|212812257)	intermediate filament protein / C. gigas	Filament
N	I	Transgelin-like protein-1(gi|223027721)	Transgelin-2 / C. gigas	Calponin homology
N+M	I	Transgelin-like protein-2 (gi|223026852)	Transgelin-2 / C. gigas	Calponin homology
M+FP	I	C1Q domain containning protein-2 (gi|238645227)	MgC1Q / M. galloprovincialis	C1q
M+FP	I	MGK-rich protein-2 (gi|223025178)	—	MGK-rich
M+FP	I	MGK-rich protein-3 (gi|223022206)	—	MGK-rich
M+FP	I	Transgelin-like protein-7 (gi|58307802)	Transgelin-2 / C. gigas	Calponin homology
M	I	Adenylate kinase-like protein (gi|145898955)	Adenylate kinase isoenzyme 1 / C. gigas	AAA
M	I	aldolase-like protein-1 (gi|223028643)	Fructose-bisphosphate aldolase / C. gigas	Glycolytic
M	I	aldolase-like protein-2 (gi|212817532)	Fructose-bisphosphate aldolase / C. gigas	Glycolytic
M	I	arginine kinase-like protein-2 (gi|237638644)	Arginine kinase/C. gigas	ATP-gua_Ptrans
M	I	arginine kinase-like protein-3 (gi|212814586)	Arginine kinase/C. gigas	ATP-gua_Ptrans
M	I	arginine kinase-like protein-4 (gi|223022743)	arginine kinase-like isoform/A. californica	ATP-gua_Ptrans
M	I	arginine kinase-like protein-5 (gi|212815663)	arginine kinase-like isoform/A. californica	ATP-gua_Ptrans
M	I	calponin-like proteins-10 (gi|223026112)	calponin-like protein / M.galloprovincialis	Calponin
M	I	calponin-like proteins-11 (gi|223026116)	calponin-like protein / M.galloprovincialis	Calponin
M	I	calponin-like proteins-12 (gi|223026117)	calponin-like protein / M.galloprovincialis	Calponin
M	I	calponin-like proteins-13 (gi|223026186)	calponin-like protein / M.galloprovincialis	Calponin
M	I	calponin-like proteins-14 (gi|239585859)	calponin-like protein / M.galloprovincialis	Calponin
M	I	calponin-like proteins-15 (gi|58306781)	calponin-like protein / M.galloprovincialis	Calponin
M	I	calponin-like proteins-9 (gi|223026062)	calponin-like protein / M.galloprovincialis	Calponin
M	I	collagen-like protein-6 (gi|58305806)	Collagen alpha-5(VI) chain / C. gigas	VWA
M	I	collagen-like protein-7 (gi|58307379)	Collagen alpha-5(VI) chain / C. gigas	VWA
M	I	collagen-like protein-8 (gi|238643251)	Collagen alpha-5(VI) chain / C. gigas	VWA
M	I	collagen-like protein-9 (gi|58306751)	collagen alpha-4(VI) chain/ A. californica	VWA
M	I	Filamin-like protein-1 (gi|238644079)	Filamin-A / C. gigas	Filamin
M	I	Filamin-like protein-2 (gi|223020794)	Filamin-C / C. gigas	Calponin homology
M	I	Filamin-like protein-3 (gi|145896099)	Filamin-C / C. gigas	Filamin-type immunoglobulin s
M	I	LKD-rich protein-1 (gi|223028013)	—	LKD-rich
M	I	LKD-rich protein-2 (gi|145889304)	—	LKD-rich
M	I	Nacrein-like protein (gi|223025603)	Nacrein-like 3 protein / P. vulgata	TSPN
M	I	SGC-rich protein (gi|58307208)	—	SGC-rich
M	I	Transgelin-like proteins-3(gi|58307171)	Transgelin-2 / C. gigas	Calponin
M	I	Transgelin-like proteins-4(gi|223026853)	Transgelin-2 / C. gigas	Calponin
M	I	Transgelin-like proteins-5(gi|223021963)	Transgelin-2 / C. gigas	Calponin
M	I	Transgelin-like proteins-6(gi|145898022)	Transgelin-2 / C. gigas	Calponin homology
FP	I	Alveoline-like proteins-1 (gi|212812802)	Alveoline-like protein / P. margaritifera	Val-rich
FP	I	Alveoline-like proteins-2 (gi|212818315)	Alveoline-like protein / P. margaritifera	Val-rich
FP	I	Alveoline-like proteins-3 (gi|212821058)	Alveoline-like protein / P. margaritifera	Val-rich
FP	I	Alveoline-like proteins-4 (gi|212821271)	Alveoline-like protein / P. margaritifera	Val-rich
FP	I	Alveoline-like proteins-5 (gi|212828530)	Alveoline-like protein / P. margaritifera	Val-rich
FP	I	Alveoline-like proteins-6 (gi|212830390)	Alveoline-like protein / P. margaritifera	Actin; Val-rich
FP	I	Alveoline-like proteins-7 (gi|212831071)	Alveoline-like protein / P. margaritifera	Val-rich
FP	I	calponin-like proteins-16 (gi|212816333)	calponin-like protein / M. galloprovincialis	Calponin
FP	I	Cathepsin-like proteins-2 (gi|238643267)	Cathepsin L / C. gigas	Pept_C1
FP	I	Cathepsin-like proteins-3 (gi|238643266)	Cathepsin L / C. gigas	Pept_C1
FP	I	Cathepsin-like proteins-4 (gi|238643268)	Cathepsin L / C. gigas	Pept_C1
FP	I	Cathepsin-like proteins-5 (gi|145895570)	Cathepsin L / C. gigas	Inhibitor_I29 and Pept_C1
FP	I	chitin synthase-like protein (gi|212820708)	chitin synthase / M. galloprovincialis	Low complexity region
FP	I	Fibronectin-like protein-3 (gi|212830512)	Fibronectin-like protein / M. californianus	FN3 (SM000060)
FP	I	filament-like protein-3 (gi|212816824)	intermediate filament protein / C. gigas	Filament
FP	I	GFS-rich protein (gi|223023579)	—	GFS-rich
FP	I	Gigasin-like protein (gi|145890922)	Gigasin-2 / C. gigas	EGF-like
FP	I	GYDK-rich protein (gi|223027775)	—	GYDK-rich
FP	I	MGK-rich protein-4 (gi|223024344)	—	MGK-rich
N	U	GYS-rich protein (gi|212817227)	—	GYS-rich
M	U	Soluble calponin-like protein-1 (gi|223023515)	calponin-like protein / M. galloprovincialis	Calponin homology
M	U	soluble calponin-like protein-2 (gi|58308196)	calponin-like protein / M. galloprovincialis	Calponin
M	U	LGDK-rich protein (gi|212830099)	—	LGDK-rich
M	U	Whirlin-like protein (gi|145900307)	Whirlin / C. gigas	PDZ
FP	U	FGV-rich protein (gi|212812207)	—	Transmembrane region; FGV-rich
N+M+FP	U+I	collagen-like protein-1 (gi|58307710)	precollagen-D / M. galloprovincialis	Collagen
N+M+FP	U+I	SRAV-rich protein (gi|58307533)	—	SRAV-rich
N+M	U+I	NSPI-like protein (gi|238643545)	NSPI2 / P. maxima	KU

“A”, “U”, and “I” represents acid-soluble, urea-soluble, and insoluble matrix, respectively.

A set of 16 proteins were identified from the USMs of three shell layers, including nine, seven, and six yielded by nacre, myostracum, and fibrous prism, respectively (Table 1 and S2 Table). Comparing to the ASMs, nine proteins were additional. For nacre, only two additional proteins, a GYS-rich protein and a SRAV-rich protein, were identified from the USM. For myostracum, five additional proteins were identified from the USM and four of them exhibited homology with SMPs previously detected in other mollusk shell, such as Calponin-like proteins [24, 25], KU domain-containing protein [26], and Whirlin-like protein [27]. For fibrous prism, only one additional protein was identified as FGV-rich protein with abundant Phe (23.9%), Gly (17.8%), and Vla (17.4%).

### Protein identification of insoluble matrices from different shell layers

The shotgun proteomic approach allowed us to identify 43, 53, 43 proteins from nacre, myostracum, and fibrous prism, respectively, yielded a set of 97 proteins from insoluble matrices (Table 1 and S3–S5 Tables). Of the 43 proteins identified from the insoluble matrix of nacre, the most abundant were a series of calponin-like proteins (8 matched ESTs). Additionally, two proteins were identified as calponin homology (CH) domain containing proteins. Six proteins were identified as homologues of reported *Mytilus* SMPs, including MUSP, Perlucin-like protein, Shell Matrix Protein, and Fibronectin-like protein. Five proteins were identified as Chitinase-like proteins with significant sequence identity (> 50%) to chitinase from other species. Four Collagen-like proteins were identified, including homologues of precollagen D and proteins containing von Willebrand factor type A (VWA) domain. Two proteins with filament domain were identified with high sequence identity (>50%) to filament proteins of other species. One proteinase inhibitor-like protein was identified with homology (32% sequence identity) to nacre serine protease inhibitor 2 (NSPI2) of *P*. *maxima* shell [26]. The insoluble matrix of nacre also contained A-rich, G-rich, M-rich, L-rich, R-rich, and S-rich proteins (Table 1 and S3 Table).

For myostracum, 53 proteins were identified from the insoluble matrix, of which, 19 proteins contained calponin domain or CH domain and seven were Collagen-like proteins, including three collagen domain-containing and four VWA domain-containing proteins. Five proteins were identified as arginine kinase-like protein with ATP-guanido phosphotransferases domain and 67–78% sequence identity to arginine kinase of other species. Three proteins were identified as filament domain-containing protein and showed sequence identity of 65–88% with filament protein of other species. One protein was identified as Nacrein-like protein with sequence identity of 42% to the Nacrein-like protein from *P*. *vulgate* shell. The other proteins included adenylate kinase-like protein, C1q domain containing protein, glycolytic domain containing protein, protease inhibitor-like protein, and various proteins with unusual amino acid composition, including S-rich, L-rich, M-rich, A-rich, and G-rich proteins (Table 1 and S4 Table).

For fibrous prism, 43 proteins were identified and the most abundant were proteins with calponin domain and/or CH domain, and Alveoline-like proteins with homology to Alveoline of pearl oyster *P*. *margaritifera* shell [28]. Five proteins were identified as Cathepsin-like proteins containing cathepsin propeptide inhibitor domain and/or cysteine protease domain. Moreover, proteins with filament domain, Collagen-like proteins, protein with C1q domain, Gigasin-like protein, and chitinase-like protein were also identified. In addition, 11 proteins were identified as uncharacterized protein, including four M-rich proteins, three G-rich proteins, two R-rich proteins, one A-rich protein, and one S-rich protein (Table 1 and S5 Table).

## Discussion

**Fig 4:**
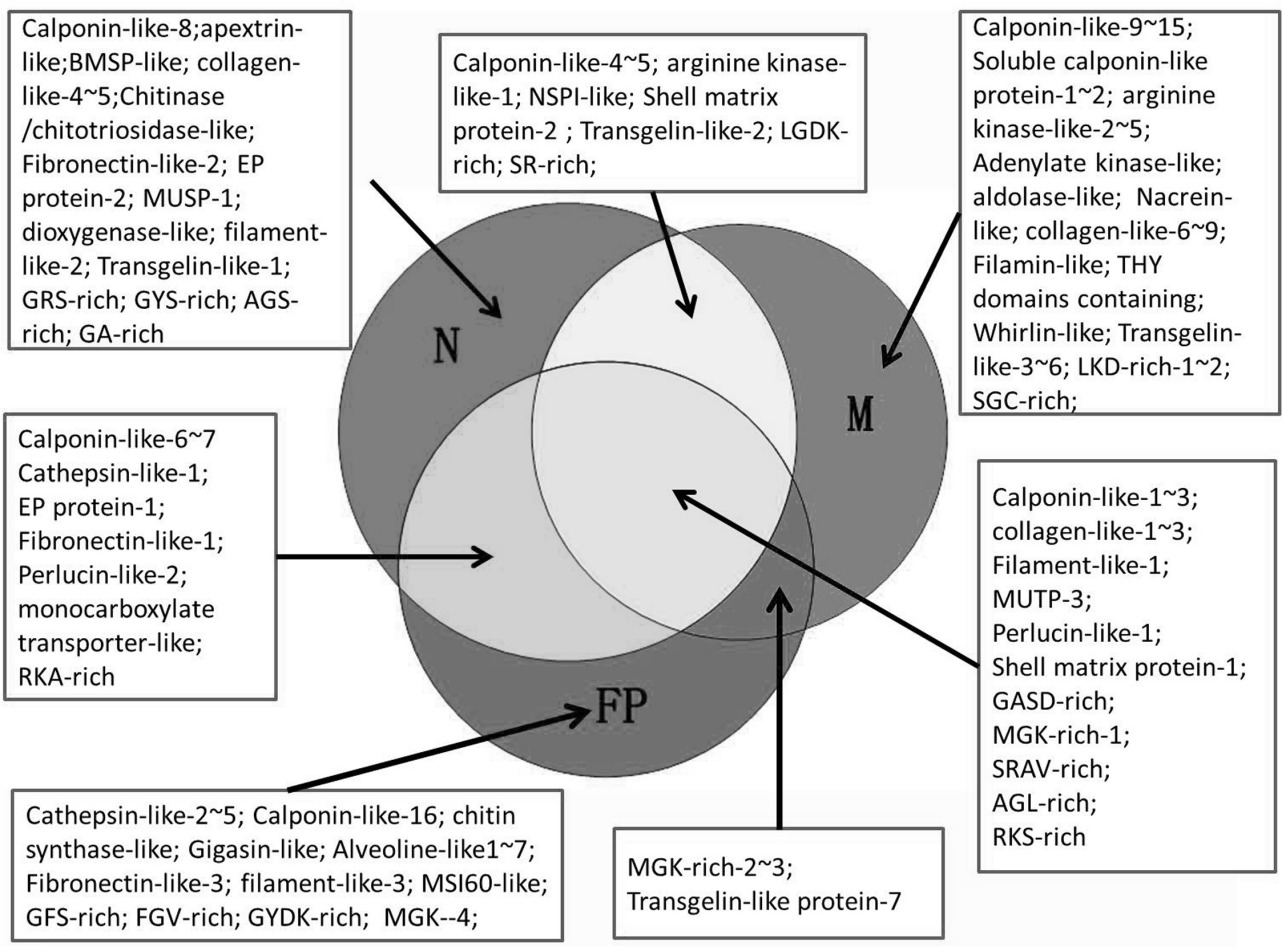
The distribution pattern of identified proteins in three shell layers of *M*. *galloprovincialis*. The proteomic data comprises identifications occurring in both soluble fraction and insoluble fraction from all three layers. N, M, and FP represent nacre, myostracum, and fibrous prism, respectively.

The mollusk shell is constructed by different calcium carbonate layers and among the most studied of them are the nacro-prismatic shells of Cambrian origin, such as bivalves, gastropods and cephalopods [29]. As shown in Fig 1, the microstructure of *M*. *galloprovincialis* shell consists of nacre, fibrous prisms, and myostracum. Nacre and fibrous prism occupied the most area of the shell sectional structure, forming a typical pattern of nacro-prismatic model. Besides that, myostracum is also an important shell layer because it is strongly attached with the adductor muscle at the adductor muscle scar, indicating a function involving in shell-muscle adhesion for this layer. In the present study, we have investigated the SMPs associated with three shell layers of *M*. *galloprovincialis* in order to search for characteristic biomineral protein signatures of various shell layers. To extract the strongly mineral linked shell proteins, we used two-step strategy (acetic acid and urea, respectively) to dissolve organic matrix of each shell layer. As a strong denaturing agent, urea has been successfully used for dissolving hydrophobic and cross-linked proteins from mussel byssus [30]. We noticed that the acetic acid-insoluble matrix can partially be dissolved in urea and additional proteins were thus identified, indicating urea can be used as an effective reagent for extracting shell proteins. By combining proteomic analysis with EST database interrogations, a whole set of 113 proteins were identified from *M*. *galloprovincialis* shell. Of this set, 15 proteins are shared by all the three layers, including three Calponin-like proteins, three Collagen-like proteins, one Filament-like protein, MUSP-3, one Perlucin-like protein, one Shell Matrix Protein, and five uncharacterized proteins with unusual amino acid composition (Table 1). Of these common proteins, Perlucin is a shell protein that could accelerate the precipitation of calcium carbonate [31], and the Shell Matrix Protein also contains a carbohydrate binding domain. These results suggest that these common shell proteins may play a key role in the formation of the whole shell of *M*. *galloprovincialis* although most of these proteins are novel proteins with unknown function. Further, of the whole 113 protein identified from *M*. *galloprovincialis* shell, 23 proteins are exclusive to nacre, 34 to myostracum, and 21 to fibrous prism. In addition, eight proteins are shared by nacre/myostracum, eight by nacre/fibrous prism, and four by myostracum/fibrous prism (Fig 4 and Table 1). The mosaic pattern of distribution of these proteins in three shell layers revealed that (i) different shell layers contained different protein sets, (ii) a possible filiation may exist among three shell layers, and (iii) the myostracum contained more unique proteins than those from nacre and fibrous prism, which may result from the role of myostracum in shell-muscle attachment. Moreover, many novel proteins were identified from *M*. *galloprovincialis* shell, dramatically increasing the SMP data of *Mytilus*. Among these novel shell proteins are myostracum-related proteins, protein inhibitor-like proteins, EGF domain-containing protein, alveoline-like proteins, and various low-complexity domain-containing proteins.

### Myostracum-related proteins

The proteins identified from myostracum is more in number than those from nacre and fibrous prism. Abundant of calponin-like proteins, transgelin-like proteins, collagen-like proteins, filamin-like proteins, and enzyme-like proteins were detected exclusively to myostracum (Table 1). Calponin domain and/or calponin-homology domain were detected in some of these proteins, including calponin-like proteins, transgelin-like proteins, and a filamin-like protein. The potential actin-binding function of calponin domain [32, 33] confers these proteins with possible roles in calponin-actin interaction, which may be involved in myostracum-muscle attachment. In addition, a protein containing thymosin beta actin-binding motif (THY domain) was also identified from myostracum, providing another candidate for actin-binding. Besides of these possible actin-binding proteins, abundant of collagen-like proteins were detected in myostracum. Earlier work revealed that collagenase can significantly reduce the adherence of muscle-shell of *C virginica* [34]. Therefore, the collagen-like proteins from myostracum may also participate in shell-muscle attachment.

### Protease inhibitor-like, Gigasin-like and Alveoline-like proteins

**Fig 5:**
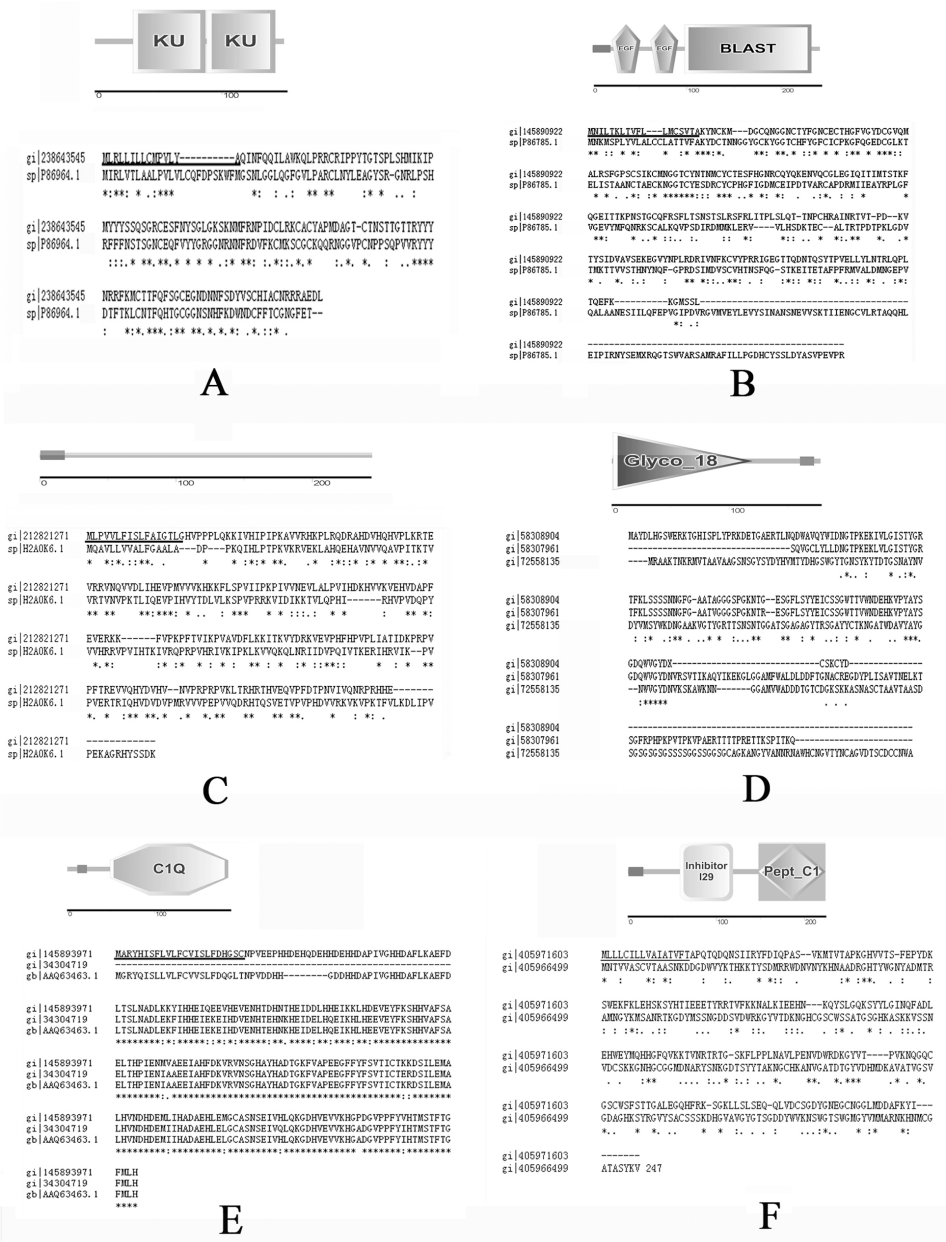
Domain organization and sequence alignment of representative novel proteins of *M*. *galloprovincialis* shell with their homologous from other shell. A: protease inhibitor-like protein of *M*. *galloprovincialis* (gi|238643545) and NSPI2 of *P*. *maxima* (sp|P86964.1); B: EGF-domain containing protein of *M*. *galloprovincialis* (gi|145890922) and Gigasin-2 of *C*. *gigas* (sp|P86785.1); C: alveoline-like protein of *M*. *galloprovincialis* (gi|212821271) and alveoline-like protein of *P*. *margaritifera* (sp|H2A0K6.1); D: chitinase-like proteins of *M*. *galloprovincialis* (gi|58308904 and gi|58307961) and acidic mammalian chitinase isoform X4 of *Saimiri boliviensis* (gi|72558135)*;* E: EP proteins of *M*. *galloprovincialis* (gi|145893971 and gi|34304719) and EP protein of *M*. *edulis* (gb|AAQ63463.1); F: Cathepsin-like protein of *M*. *galloprovincialis* (gi|238641522) and Cathepsin of *C*. *gigas* (gi|405966499). Signal peptides are underlined; “*”represents identical amino acids; “:” and “.” represent similar amino acids; “-”represents the gaps inserted in the sequence.

A potential protease inhibitor was identified from both of the nacre and myostracum. This 149-AA long sequence, with a 15-AA long signal peptide and KU domain in its sequence, exhibited 32% sequence identity with NSPI2, a KU domain-containing protein from the oyster *P*. *maxima* shell (Fig 5A) [28]. Protease inhibitors have also been identified in other mollusks shell [16, 35] and the protease inhibitor domain has been detected in other SMP, such as lustrin A, an insoluble protein identified from the nacre of abalone *H*. *rufescens* [36]. These results suggested a protecting system that precludes extracellular proteolysis during the shell formation. An EGF domain-containing protein, with 241-AA long sequence including an 18-AA long signal peptide, was identified from the insoluble matrix of fibrous prism. This protein showed 29% sequence identity with gigasin-2, an EGF domain-containing protein of *C*. *gigas* shell [18] (Fig 5B). The EGF domain has already been found in several reported SMPs from other Mollusca, such as perlustrin from *H*. *laevigata* [37] and SMPs from *L*. *gigantea* [38]. Abundant of val-rich proteins were identified from the insoluble matrix of fibrous prism. These proteins, characterized by abundant Val, Pro, and Lys residues and showed 30~40% of sequence identity with alveoline-like protein, a prismatic SMP of *P*. *margaritifera* [28] (Fig 5C).

### Other shell proteins of M. galloprovincialis

Chitin metabolic-related proteins were identified in this study, including 5 chitinase/chitotriosidase-like proteins with Glyco_18 domain from N layer (Fig 5D) and a chitin synthase-like protein from FP layer. Chitin is a major non-protein component of mollusc shells [39–41] and involved in constitution a framework binding silk-like proteins and acidic proteins [42]. The identification of chitin metabolic-related proteins in mussel shell suggests a possible roll of these proteins in shell construction or repair by modifying the chitin framework. Two histidine-rich proteins were identified with high homology to *M*. *edulis* EP protein (Fig 5E). Both of the two sequences contain a C1Q domain in the C-terminus and abundant His (14.3%). The function of EP protein in shell formation is unknown. However, an earlier work identified a histidine-rich calcium-binding protein of the sarcoplasmic reticulum of the rabbit muscle with 13% of His [43], suggesting that the positively charged His residue may also play a functional role in the binding of calcium ions.

A set of cathepsin-like proteins were identified mainly from fibrous prism. These proteins showed high sequence identity (70~80%) with cathepsin L, a protease previously identified from *C*.*gigas* shell [27] (Fig 5F). Together with the protease inhibitor-like identified in the nacre and myostracum, a protein degradation/protection system has been found in mussel shell and may play a role in regulating the catabolic of shell protein during shell formation and growing.

### Uncharacterized *M*. *galloprovincialis* shell proteins

Many identified proteins contain unusual amino acid composition, or short tandem repeats, or blocks of identical amino acids. Most of these proteins exhibited neither homology nor domains except for low complexity regions (LCRs). We observed that LCRs are different among the proteins of three layers. For example, GRS-rich, AGS-rich, and GYS-rich types are only presented in the proteins of nacre; SGC-rich and LKD-rich in the proteins of myostracum; FGV-rich, GFS-rich, and GYDK-rich in the proteins of fibrous prism. Some of these primary sequence features are also found in other SMPs that have been proposed to adopt a specific structure when binding to the calcium carbonate crystal surface [26, 44].

In addition, we also observed some intracellular proteins were presented in *M*. *galloprovincialis* shell, especially in insoluble matrix, such as actin/paramyosin, ubiquitin/polyubiquitin, and tubulin (data not shown). Proteins of previously known intracellular location were also found in other mollusk shell proteome [27, 28, 38]. In the present study, before LC-MS/MS analysis, the shell samples were processed by sodium hydroxide bleaching to eliminate potential organic contaminants on the shell, thus, it is puzzling and hard to explain the presence of these intracellular proteins in the shell, however, we cannot exclude the possibility that these intracellular protein may be transported into extrapallial fluid via special pathway and may bind with the shell or SMPs. As true intra-crystalline components, although probably without any function, intracellular proteins may not be removed even by rigorous sodium hydroxide cleaning. Furthermore, the insoluble matrices contained more of these intracellular components, one may conclude that many of them were already structurally modified and aggregated before incorporation into the shell.

## Conclusions

*Mytilus* shell is a good model of nacro-prismatic structure for studying the shell formation and SMPs responsible for different shell microstructures. A parallel proteomics analysis was performed for three layers, nacre, myostracum, and fibrous prism, which form the shell of *M*. *galloprovincialis*. A set of 54, 61, and 48 proteins were identified from nacre, myostracum, and fibrous prism respectively. For each shell layer, about a half of identified proteins were unique and the others are shared by two or all of the three layers, which indicated a possible evolutionary relationship among the three layers. In addition, the myostracum contained the largest protein set, highlighting the important function of myostracum in attachment with adductor muscle. Moreover, many novel shell proteins were identified, including the proteins with possible or established link to biomineralization and some uncharacterized proteins with unusual amino acid composition. These data are useful for understanding the roles of SMPs associated to the formation of different shell layers (nacre *vs*. myostracum *vs*. fibrous prism) or different morphology of calcium carbonate (aragonite *vs*. calcite), and the identified protein set of myostracum provides candidates for further exploring the molecular mechanism of adductor muscle-shell attachment.

## Supporting Information

S1 Fig HPLC elution profiles of acid-soluble matrices (A~C) and urea-soluble matrices (D~F) from nacre (N), myostracum (M), and fibrous prism (FP), respectively. I~IV represent the fraction collected for LC-MS/MS analysis.
(TIF)

S1 Table Protein identification from acid-soluble matrices of nacre, myostracum, and fibrous prism. (DOCX)

S2 Table Protein identification from urea-soluble matrices of nacre, myostracum, and fibrous prism. (DOCX)

S3 Table Protein identification from insoluble matrix of nacre. (DOCX)

S4 Table Protein identification from insoluble matrix of myostracum. (DOCX)

S5 Table Protein identification from insoluble matrix of fibrous prism. (DOCX)
