# Correction: Layer-by-Layer Proteomic Analysis of *Mytilus galloprovincialis* Shell

**DOI:** 10.1371/journal.pone.0137487

**Published:** 2015-09-03

**Authors:** Peng Gao, Zhi Liao, Xin-xing Wang, Lin-fei Bao, Mei-hua Fan, Xiao-min Li, Chang-wen Wu, Shu-wei Xia

There is an error in affiliation 1 for authors Peng Gao and Shu-wei Xia. Affiliation 1 should be: College of Chemistry and Chemical Engineering, Ocean University of China, Qingdao, China.
