# Supplementary figures and images for: Layer-by-Layer Proteomic Analysis of Mytilus galloprovincialis Shell
Source: PLoS One. 2015 Jul 28;10(7):e0133913. doi: 10.1371/journal.pone.0133913 (PMC4517812; doi:10.1371/journal.pone.0133913)

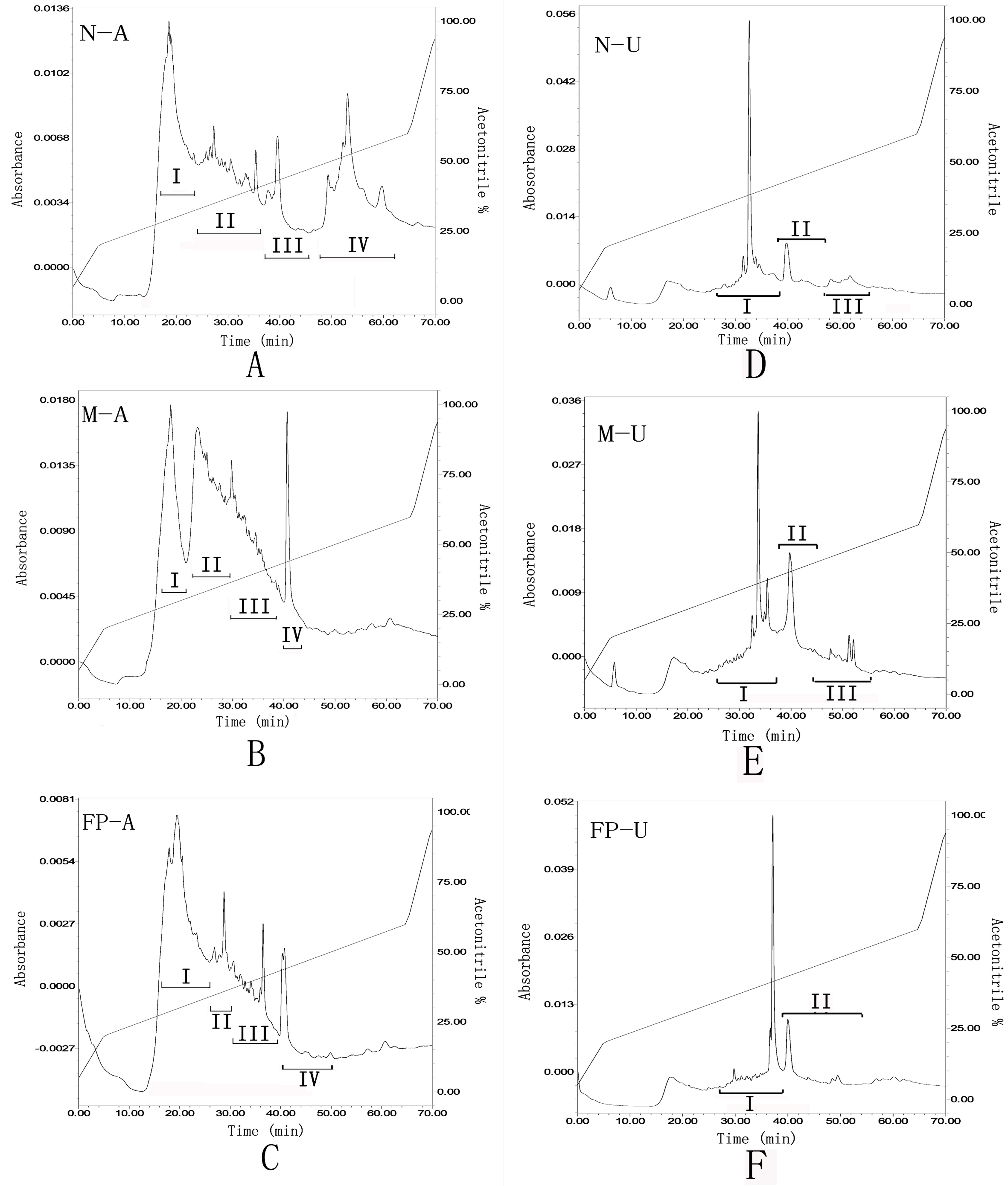

Supplement: S1 Fig — I~IV represent the fraction collected for LC-MS/MS analysis. (TIF) [file pone.0133913.s001.tif]
