# Supplementary material for: Layer-by-Layer Proteomic Analysis of Mytilus galloprovincialis Shell
Source: PLoS One. 2015 Jul 28;10(7):e0133913. doi: 10.1371/journal.pone.0133913 (PMC4517812; doi:10.1371/journal.pone.0133913)
Supplement: S1 Table — (DOCX) [file pone.0133913.s002.docx]

**S1 Table**

| **Fraction** | **Matched EST** | **Organism of matched EST** | **Homology [Organism] ID;sequence identity%** | **Domain (ID) or features** | **Protein score** | **Matched peptides** | **Peptide score** | **EST-derived seuqence (the signal peptides are underlined; "?" denotes undetermined amino acids, "*" denotes the stop codon)** |
| --- | --- | --- | --- | --- | --- | --- | --- | --- |
| **FP-A-I** | gi\|58308563 | *M. galloprovincialis* | — | poly-Ala;Low complexity region; Ala (41.2%), Gly ((21.8%), Leu (8.8%) | 656 | AAAAAGASAAAGGSGGTLR | 101.55 | ?FFFFFGYGYGGALDIDLGDLEELLGGLDTIDLEDAAVLSALGLGGGSGLGGGSAAAAAAAAAAAAGGLGGGSAAAAAAAAAAAAGGLGGGSAAAAAAAAAAAGGAGGIGGSSAAAAAAAAAAAGRRAAAAAGASAAAGGSGGTLRQRLISRIIARRQSAASAAAAAAASAF* |
|  |  |  |  |  |  | QSAASAAAAAAASAF | 29.19 |  |
|  | gi\|212814500 | *M. californianus* | MSI60-like protein  [M. californianus] sp\|P86857.1\| 100% | SCOP domain d1gkub1 | 110 | LYAYDYYK | 38.38 | ?SAAASAARRARLLNLLFARSSAAAAASAAASAGAGSGSGGFGLGSRFLGGGRGGSRAAASAVASAGASARGGGGGGGGSSSAAAAAAAAAAAARNANLRGWLVASGVGAGAGAGAGAGAGAGAGAGAGAGGGSGGGGGGGSGSGGSGGSGGSGGSGGNDGNDGNDGSSSRGVKLYAYDYYKDDKKGSND* |
|  | gi\|58306883 | *M. galloprovincialis* | Perlucin-like protein [M. galloprovincialis] sp\|P86854.1\|; 100% | C-type lectin (CTL) or carbohydrate-recognition domain (CRD); SM000034 | 89 | ITDSEENSWVVDMITK | 89.29 | MGKLTVVGILTLFIFYIVAASGKCTAPVNCPAGWKKYKTNCYFFSPDGKNWHDAAKQCQTMGGYLVKITDSEENSWVVDMITKSVKHKYGYWMGMADLKNEGDWRWVNDSSAVSYSNWHRGQPNNANNEDCGHFWSAVNYEWNDIVCNTDQMGYIC* |
| **FP-A-II** | gi\|58308563 | *M. galloprovincialis* | — | poly-Ala;Low complexity region; Ala (41.2%), Gly ((21.8%), Leu (8.8%) | 539 | AAAAAGASAAAGGSGGTLR | 91.73 | ?FFFFFGYGYGGALDIDLGDLEELLGGLDTIDLEDAAVLSALGLGGGSGLGGGSAAAAAAAAAAAAGGLGGGSAAAAAAAAAAAAGGLGGGSAAAAAAAAAAAGGAGGIGGSSAAAAAAAAAAAGRRAAAAAGASAAAGGSGGTLRQRLISRIIARRQSAASAAAAAAASAF? |
|  |  |  |  |  |  | LISRIIAR | 29.15 |  |
| **FP-A-III** | gi\|212815602 | *M. californianus* | Fibronectin-like protein [M. californianus] sp\|P86861.1\|; 100% | Fibronectin type 3 domain (SM000060) | 120 | QDMFTTAENQLR | 74.54 | MFSFGIILLTVVSFTNAQWRQDMFTTAENQLRSIVQNGQTLLDFIYQERQKHGGGNMTGGSLMSHNVAYSSFINDVETRLADMEQATQELVRIMRTCPDAPLAPPPPTNVIVESTTIDNVSSIVVKWDPPFNPPENMQYKVYFVPVDQNGMQTAGEVVFRICDSTQTIASITDLTPRSRYRIRVGAVAGAVAEGASMPLNVKTP? |
| **FP-A-IV** | gi\|58306883 | *M. galloprovincialis* | Perlucin-like protein [M. galloprovincialis] sp\|P86854.1\|; 100% | C-type lectin (CTL) or carbohydrate-recognition domain (CRD); SM000034 | 339 | ITDSEENSWVVDMITK | 126.37 | MGKLTVVGILTLFIFYIVAASGKCTAPVNCPAGWKKYKTNCYFFSPDGKNWHDAAKQCQTMGGYLVKITDSEENSWVVDMITKSVKHKYGYWMGMADLKNEGDWRWVNDSSAVSYSNWHRGQPNNANNEDCGHFWSAVNYEWNDIVCNTDQMGYIC* |
|  | gi\|145893971 | *M. californianus* | EP protein precursor [M. edulis] gb\|AAQ63463.1\| 84% | C1Q domain,SM000110 | 139 | AEFDLTSLNADLK | 79.78 | MARYHISFLVLFCVISLFDHGSCNPVEEPHHDEHQDEHHDEHHDAPIVGHHDAFLKAEFDLTSLNADLKKYIHHEIQEEVHEVENHTDHNTHEIDDLHHEIKKLHDEVEYFKSHHVAFSAELTHPIENMVAEEIAHFDKVRVNSGHAYHADTGKFVAPEEGFFYFSVTICTKKDSILEMALHVNDHDEMLIHADAEHLEMGCASNSEIVHLQKGDHVEVVKHGPDGVPPFYVHTMSTFTGFMLH* |
|  |  |  |  |  |  | KLHDEVEYFK | 42.21 |  |
|  |  |  |  |  |  | LHDEVEYFK | 56.46 |  |
|  | gi\|58308563 | *M. galloprovincialis* | — | poly-Ala containing;Low complexity region; Ala (41.2%), Gly ((21.8%), Leu (8.8%) | 98 | AAAAAGASAAAGGSGGTLR | 98.05 | ?FFFFFGYGYGGALDIDLGDLEELLGGLDTIDLEDAAVLSALGLGGGSGLGGGSAAAAAAAAAAAAGGLGGGSAAAAAAAAAAAAGGLGGGSAAAAAAAAAAAGGAGGIGGSSAAAAAAAAAAAGRRAAAAAGASAAAGGSGGTLRQRLISRIIARRQSAASAAAAAAASAF? |
|  |  |  |  |  |  |  |  |  |
| **M-A-I** | gi\|58306883 | *M. galloprovincialis* | Perlucin-like protein [M. galloprovincialis] sp\|P86854.1\|; 100% | C-type lectin (CTL) or carbohydrate-recognition domain (CRD); SM000034 | 205 | ITDSEENSWVVDMITK | 126.22 | MGKLTVVGILTLFIFYIVAASGKCTAPVNCPAGWKKYKTNCYFFSPDGKNWHDAAKQCQTMGGYLVKITDSEENSWVVDMITKSVKHKYGYWMGMADLKNEGDWRWVNDSSAVSYSNWHRGQPNNANNEDCGHFWSAVNYEWNDIVCNTDQMGYIC* |
| **M-A-II** | gi\|58308563 | *M. galloprovincialis* | — | poly-Ala containing;Low complexity region; Ala (41.2%), Gly ((21.8%), Leu (8.8%) | 614 | AAAAAGASAAAGGSGGTLR | 97.46 | ?FFFFFGYGYGGALDIDLGDLEELLGGLDTIDLEDAAVLSALGLGGGSGLGGGSAAAAAAAAAAAAGGLGGGSAAAAAAAAAAAAGGLGGGSAAAAAAAAAAAGGAGGIGGSSAAAAAAAAAAAGRRAAAAAGASAAAGGSGGTLRQRLISRIIARRQSAASAAAAAAASAF? |
|  |  |  |  |  |  | LISRIIAR | 20.45 |  |
|  | gi\|145887968 | *M. californianus* | Shell matrix protein [M. californianus] sp\|P86860.1\|; 100% | Laminin_G_3 domain PF13385; (carbohydrate binding domains) | 130 | GGLAFDYSHISLR | 70.84 | ?YLAPQYGGLRIRACPSGTIYSENQCRYKSNMNGNGGLRGSARKQFRQCSAEFKINFDDGFKDISKGGLAFDYSHISLRRGKGVFVGNSKLYIWGFQSRFLGKTFAIRMKVKIKRGAGKYRPEPIISNCGPNGDSSVEIVVHRGKVIFKAKTSDNPEAVFITEDYDDDKWTDLTYYYDGNHFGGSCNGRPFRQRTGGNLEIRDNPMTIGLCTGQNGFHGEIDELEIYTACIPKDM* |
|  |  |  |  |  |  | TGGNLEIR | 71.35 |  |
|  | gi\|212814580 | *M. californianus* | MUSP-3 [M. californianus]sp\|P86859.1\|; 100% | — | 103 | VDNGQGIAFR | 67.68 | ?RVHYYLHSCKGRTCYNSERHKIYKMLKGIILIVTIQLVNANFFGVFGKPLYNPFNKDKYMIDFITTFNKLMNMKQPQFPHPKSYPGFPPLFPGIKGKKSVFKTIDFTDMAPGSKKTFRVDNGQGIAFRSKSGNAGGMSFSSGTGGGKGFAFGGTLGGGSNGEFVMSQSGPGLKGGKVTYSKGVPKFAKGLFGMLPFFK* |
|  | gi\|145887813 | *M. californianus* | Shell matrix protein [M. californianus] sp\|P86860.1\|; 100% | — | 93 | YGYLAPQYGGLR | 50.11 | ?KGYQYLPVVLKVIAMTTIKNVSEHQAGICVTMSVKLPAILTRQLAQTSCPSLPDPMNRYGYLAPQYGGLRIRACPSGTIYSENQCRYKSNMNGNGGLRGSARKQFRQCSAEFKINFDDGFKDISKGGLAFDYSHISLRRGKGVFVGNSKLYIWGFQSRFLGKTFAIRMKVKIKRGAGKYRPEPIISNCGPNGDSSVEIVVHRGKVIFKAKH? |
|  |  |  |  |  |  | GGLAFDYSHISLR | 70.84 |  |
| **M-A-III** | gi\|58308563 | *M. galloprovincialis* | — | poly-Ala;Low complexity region; Ala (41.2%), Gly ((21.8%), Leu (8.8%) | 1484 | AAAAAGASAAAGGSGGTLR | 108.62 | ?FFFFFGYGYGGALDIDLGDLEELLGGLDTIDLEDAAVLSALGLGGGSGLGGGSAAAAAAAAAAAAGGLGGGSAAAAAAAAAAAAGGLGGGSAAAAAAAAAAAGGAGGIGGSSAAAAAAAAAAAGRRAAAAAGASAAAGGSGGTLRQRLISRIIARRQSAASAAAAAAASAF? |
|  | gi\|238649869 | *M. galloprovincialis* | — | 5 Thymosin beta actin-binding motif(THY domain);SM00152 | 105 | AALPSADPQLLGAISK | 64.59 | MATKAALPSADPQLLGAISKATPESLHHVQTDVKNPLPSKEAIAQEKTEQELMSGIEGFDAGKLKPTETQEKNPLPDPSAITAEKAERYRKQSIEDFNKKKLKRANTEEKNTLPDADAIEQEKRECEMRHSIGDFNKSKLRHSQTEVKNPLPSTEAINLEKQEVEKIQEIEGFKKDSLKHTEPTVKNVLPDQDTLDAE? |
| **M-A-IV** | gi\|58306883 | *M. galloprovincialis* | Perlucin-like protein [M. galloprovincialis] sp\|P86854.1\|; 100% | C-type lectin (CTL) or carbohydrate-recognition domain (CRD); SM000034 | 469 | ITDSEENSWVVDMITK | 129.03 | ?RLKVVNCKQQIFTMGKLTVVGILTLFIFYIVAASGKCTAPVNCPAGWKKYKTNCYFFSPDGKNWHDAAKQCQTMGGYLVKITDSEENSWVVDMITKSVKHKYGYWMGMADLKNEGDWRWVNDSSAVSYSNWHRGQPNNANNEDCGHFWSAVNYEWNDIVCNTDQMGYIC* |
|  |  |  |  |  |  |  |  |  |
|  | gi\|212814580 | *M. californianus* | MUSP-3 [M. californianus]sp\|P86859.1\|; 100% | — | 141 | VDNGQGIAFR | 79.79 | ?RVHYYLHSCKGRTCYNSERHKIYKMLKGIILIVTIQLVNANFFGVFGKPLYNPFNKDKYMIDFITTFNKLMNMKQPQFPHPKSYPGFPPLFPGIKGKKSVFKTIDFTDMAPGSKKTFRVDNGQGIAFRSKSGNAGGMSFSSGTGGGKGFAFGGTLGGGSNGEFVMSQSGPGLKGGKVTYSKGVPKFAKGLFGMLPFFK* |
|  |  |  |  |  |  |  |  |  |
|  | gi\|58308563 | *M. galloprovincialis* | — | poly-Ala; Low complexity region; Ala (41.2%), Gly ((21.8%), Leu (8.8%) | 133 | AAAAAGASAAAGGSGGTLR | 87.2 | ?FFFFFGYGYGGALDIDLGDLEELLGGLDTIDLEDAAVLSALGLGGGSGLGGGSAAAAAAAAAAAAGGLGGGSAAAAAAAAAAAAGGLGGGSAAAAAAAAAAAGGAGGIGGSSAAAAAAAAAAAGRRAAAAAGASAAAGGSGGTLRQRLISRIIARRQSAASAAAAAAASAF? |
|  |  |  |  |  |  |  |  |  |
|  | gi\|58307858 | *M. californianus* | distal byssal thread collagen [synthetic construct] gb\|AFM30918.1\|; 91% | Internal repeats; Low complexity region | 123 | GSVGDQGAQGDQGATGADGK | 109.88 | ?TRTQGPTGSEGPVGAPGPKGSVGDQGAQGDQGATGADGKPGDRGPDGETGPQGPAGPKGQVGDQGKPGAKGETGDQGARGEAGKAGEQGPGGIQGPKGPVGGQGPAGPAGPLGPQGPMGERGPQGPTGSEGPVGAPGPKGSVGDQGAQGDQGATGADGKKGEPGERGQQGAAGP? |
|  |  |  |  |  |  | AGEQGPGGIQGPK | 38.72 |  |
|  |  |  |  |  |  |  |  |  |
| **N-A-I** | gi\|58308563 | *M. galloprovincialis* | — | poly-Ala; Low complexity region; Ala (41.2%), Gly ((21.8%), Leu (8.8%) | 397 | AAAAAGASAAAGGSGGTLR | 80.52 | ?FFFFFGYGYGGALDIDLGDLEELLGGLDTIDLEDAAVLSALGLGGGSGLGGGSAAAAAAAAAAAAGGLGGGSAAAAAAAAAAAAGGLGGGSAAAAAAAAAAAGGAGGIGGSSAAAAAAAAAAAGRRAAAAAGASAAAGGSGGTLRQRLISRIIARRQSAASAAAAAAASAF? |
|  |  |  |  |  |  |  |  |  |
|  | gi\|212814580 | *M. californianus* | MUSP-3 [M. californianus]sp\|P86859.1\|; 100% | — | 96 | VDNGQGIAFR | 82.85 | ?RVHYYLHSCKGRTCYNSERHKIYKMLKGIILIVTIQLVNANFFGVFGKPLYNPFNKDKYMIDFITTFNKLMNMKQPQFPHPKSYPGFPPLFPGIKGKKSVFKTIDFTDMAPGSKKTFRVDNGQGIAFRSKSGNAGGMSFSSGTGGGKGFAFGGTLGGGSNGEFVMSQSGPGLKGGKVTYSKGVPKFAKGLFGMLPFFK* |
|  |  |  |  |  |  |  |  |  |
|  | gi\|145887968 | *M. californianus* | Shell matrix protein [M. californianus] sp\|P86860.1\|; 100% | Laminin_G_3 domain PF13385 | 92 | TGGNLEIR | 59.07 | ?YLAPQYGGLRIRACPSGTIYSENQCRYKSNMNGNGGLRGSARKQFRQCSAEFKINFDDGFKDISKGGLAFDYSHISLRRGKGVFVGNSKLYIWGFQSRFLGKTFAIRMKVKIKRGAGKYRPEPIISNCGPNGDSSVEIVVHRGKVIFKAKTSDNPEAVFITEDYDDDKWTDLTYYYDGNHFGGSCNGRPFRQRTGGNLEIRDNPMTIGLCTGQNGFHGEIDELEIYTACIPKDM* |
|  |  |  |  |  |  |  |  |  |
| **N-A-II** | gi\|58308563 | *M. galloprovincialis* | — | poly-Ala; Low complexity region; Ala (41.2%), Gly ((21.8%), Leu (8.8%) | 1051 | AAAAAGASAAAGGSGGTLR | 112.61 | ?FFFFFGYGYGGALDIDLGDLEELLGGLDTIDLEDAAVLSALGLGGGSGLGGGSAAAAAAAAAAAAGGLGGGSAAAAAAAAAAAAGGLGGGSAAAAAAAAAAAGGAGGIGGSSAAAAAAAAAAAGRRAAAAAGASAAAGGSGGTLRQRLISRIIARRQSAASAAAAAAASAF? |
|  |  |  |  |  |  | QSAASAAAAAAASAF | 25.6 |  |
|  | gi\|145887968 | *M. californianus* | Shell matrix protein [M. californianus] P86860.1; 100% | Laminin_G_3 domain PF13385; (carbohydrate binding domains) | 276 | LYIWGFQSR | 30.3 | ?YLAPQYGGLRIRACPSGTIYSENQCRYKSNMNGNGGLRGSARKQFRQCSAEFKINFDDGFKDISKGGLAFDYSHISLRRGKGVFVGNSKLYIWGFQSRFLGKTFAIRMKVKIKRGAGKYRPEPIISNCGPNGDSSVEIVVHRGKVIFKAKTSDNPEAVFITEDYDDDKWTDLTYYYDGNHFGGSCNGRPFRQRTGGNLEIRDNPMTIGLCTGQNGFHGEIDELEIYTACIPKDM* |
|  |  |  |  |  |  | TGGNLEIR | 67.82 |  |
|  |  |  |  |  |  | TSDNPEAVFITEDYDDDK | 96.91 |  |
|  |  |  |  |  |  | GGLAFDYSHISLR | 73.44 |  |
|  | gi\|223026932 | *M. galloprovincialis* | — | Low complexity region; Gly (22.8%), Arg (9.5%), Ser (8.2%) | 198 | GLGSGGGAGSGGGVDGER | 123.26 | MGMLTLLLVSFVGSAYCTVKSYQDYAPGYGGGSEGGYNVEYGSGDGRGLGSGGGAGSGGGVDGERRRQGNEGKGDDREWCTCRSGQCIKGVFRLDKQCILPQLFPWKLDTCCKALPGRVKRRSYSGAVGSGTSGGVRYIQRPGLRRRYGYWASGNPFG* |
|  | gi\|212816250 | *M. californianus* | — | Transmembrane region; Ala (43.7%), Gly (22.8%), Ser (10.2%) | 108 | AAAAASASAAASGSSGGTLR | 108.15 | ?RGGAELDEVIASLSGGGGGDGFGLGLGGAVDIDLGDLEELLGVDLSDLDEATLLALLGGGGAGGSAAAAAAAAAAAGGLGGGSAAAAAAAAAAAAGGLGGSSAAAAAAAAAAAAGGLGGSSAAAAAAAAAAAAGGLGGSSAAAAAAAAAAAAGGFGGSSAAAAAAAAAAAGGVGGVGGSSAAAAAAAAAAAGRRAAAAASASAAASGSSGGTLRQRLISKIIARRQAAASAAAAASASASGGGGAXGGAXGASA? |
|  | gi\|154348940 | *M. galloprovincialis* | apextrin-like protein [M. galloprovincialis] gb\|AEK10750.1\|; 71% | internal repeats | 94 | WGVLPDGEYGR | 29.7 | MVNMFILFLMIASACAVGWPDESYSLPKPKSGCPTGWAMGFRNQDNEDTHNINSVTPPDPDHHFDGYFGRNTIMCYCTKTTYSGYGSWPSGNYCIARYGGSCPLGFTCNGSIYWDDEDDSNANSKWGVLPDGEYGRNTKIYYCCRSDRSAYSYIDLPAREPFYLYKYTSTCQRVRGMNVTEESVKMDDEDSMNNSSDDGCHPKKTDTTVVHYCYYS* |
|  |  |  |  |  |  | SAYSYIDLPAR | 58.55 |  |
|  | gi\|212814599 | *M. californianus* | BMSP [M. galloprovincialis] dbj\|BAK86420.1\|;34% | Chitin-binding domain type 2 (SM000494) | 93 | LQATNQAIAGGSIR | 64.47 | ?EDNTHTIRKGKEHDLSSGAASVSIGSDSSKTTKIAEAVKALTALQKLQATNQAIAGGSIRLKPTLPDTTYKLPVNGGSLNTLSEKLSQQINLKLKTNDVEVLSRKRLLSAVLGKQPLLTKVAPSAPVAATASPNLEALCLLYLGAFIDSIGYAPFPGKCNKLVQCFYLGGKLQTVARDCPAGMFWDQKQLLCRPPDDVICLEDQCLIHGTRHYRRDGGCNCFYKCIEGISVPSCCPKGYRYDDDKECV? |
|  |  |  |  |  |  |  |  |  |
|  | gi\|145887813 | *M. californianus* | Shell matrix protein [M. californianus] sp\|P86860.1\|; 100% | — | 90 | YGYLAPQYGGLR | 21.62 | MTTIKNVSEHQAGICVTMSVKLPAILTRQLAQTSCPSLPDPMNRYGYLAPQYGGLRIRACPSGTIYSENQCRYKSNMNGNGGLRGSARKQFRQCSAEFKINFDDGFKDISKGGLAFDYSHISLRRGKGVFVGNSKLYIWGFQSRFLGKTFAIRMKVKIKRGAGKYRPEPIISNCGPNGDSSVEIVVHRGKVIFKAKH? |
|  |  |  |  |  |  | GGLAFDYSHISLR | 73.44 |  |
|  |  |  |  |  |  | LYIWGFQSR | 30.3 |  |
| **N-A-III** | gi\|58306883 | *M. galloprovincialis* | Perlucin-like protein [M. galloprovincialis] sp\|P86854.1\|; 100% | C-type lectin (CTL) or carbohydrate-recognition domain (CRD); SM000034 | 456 | ITDSEENSWVVDMITK | 126.78 | ?RLKVVNCKQQIFTMGKLTVVGILTLFIFYIVAASGKCTAPVNCPAGWKKYKTNCYFFSPDGKNWHDAAKQCQTMGGYLVKITDSEENSWVVDMITKSVKHKYGYWMGMADLKNEGDWRWVNDSSAVSYSNWHRGQPNNANNEDCGHFWSAVNYEWNDIVCNTDQMGYIC* |
|  |  |  |  |  |  |  |  |  |
|  |  |  |  |  |  |  |  |  |
|  | gi\|58308563 | *M. galloprovincialis* | — | poly-Ala; Low complexity region; Ala (41.2%), Gly ((21.8%), Leu (8.8%) | 194 | AAAAAGASAAAGGSGGTLR | 95.67 | ?FFFFFGYGYGGALDIDLGDLEELLGGLDTIDLEDAAVLSALGLGGGSGLGGGSAAAAAAAAAAAAGGLGGGSAAAAAAAAAAAAGGLGGGSAAAAAAAAAAAGGAGGIGGSSAAAAAAAAAAAGRRAAAAAGASAAAGGSGGTLRQRLISRIIARRQSAASAAAAAAASAF? |
|  | gi\|212815602 | *M. californianus* | Fibronectin-like protein  [M. californianus] sp\|P86861.1\|; 100% | Fibronectin type 3 domain; SM000060 | 178 | QDMFTTAENQLR | 60.55 | MFSFGIILLTVVSFTNAQWRQDMFTTAENQLRSIVQNGQTLLDFIYQERQKHGGGNMTGGSLMSHNVAYSSFINDVETRLADMEQATQELVRIMRTCPDAPLAPPPPTNVIVESTTIDNVSSIVVKWDPPFNPPENMQYKVYFVPVDQNGMQTAGEVVFRICDSTQTIASITDLTPRSRYRIRVGAVAGAVAEGASMPLNVKTP? |
|  |  |  |  |  |  | LADMEQATQELVR | 76.84 |  |
|  |  |  |  |  |  |  |  |  |
|  | gi\|223021659 | *M. galloprovincialis* | MUSP-1  [M. galloprovincialis] sp\|P86853.1\|; 100% | — | 175 | LLAGYPTIK | 73.32 | MISKYCLFVIVLGTTGTALVLTNDSNKLQNVKAVIAIQDKVLHFHDHTTDCVGELMCIFAALPESERNQTLSIPLGLLTTIATDKGRDRYSSIYAEAKKLLAGYPTIKHALNAAENGHSTKDKNVCASMYSKCPFEPDDLLDTINDLEDITTLFSKNVFGKVIADAIEYNYTQVGMTQTHS? |
|  |  |  |  |  |  | YSSIYAEAK | 32.4 |  |
|  |  |  |  |  |  | KLLAGYPTIK | 63.08 |  |
|  |  |  |  |  |  | DRYSSIYAEAK | 49.81 |  |
| **N-A-IV** | gi\|58306883 | *M. galloprovincialis* | Perlucin-like protein;[M. galloprovincialis];sp\|P86854.1\|; 100% | C-type lectin (CTL) or carbohydrate-recognition domain (CRD); SM000034 | 358 | ITDSEENSWVVDMITK | 92.3 | ?RLKVVNCKQQIFTMGKLTVVGILTLFIFYIVAASGKCTAPVNCPAGWKKYKTNCYFFSPDGKNWHDAAKQCQTMGGYLVKITDSEENSWVVDMITKSVKHKYGYWMGMADLKNEGDWRWVNDSSAVSYSNWHRGQPNNANNEDCGHFWSAVNYEWNDIVCNTDQMGYIC* |
|  |  |  |  |  |  |  |  |  |
|  |  |  |  |  |  |  |  |  |
|  | gi\|223021659 | *M. galloprovincialis* | MUSP-1 [M. galloprovincialis] sp\|P86853.1\|A;100% | — | 358 | LQNVKAVIAIQDK | 53.45 | MISKYCLFVIVLGTTGTALVLTNDSNKLQNVKAVIAIQDKVLHFHDHTTDCVGELMCIFAALPESERNQTLSIPLGLLTTIATDKGRDRYSSIYAEAKKLLAGYPTIKHALNAAENGHSTKDKNVCASMYSKCPFEPDDLLDTINDLEDITTLFSKNVFGKVIADAIEYNYTQVGMTQTHS? |
|  |  |  |  |  |  | AVIAIQDK | 48 |  |
|  |  |  |  |  |  | DRYSSIYAEAK | 40.9 |  |
|  |  |  |  |  |  | YSSIYAEAK | 43.01 |  |
|  |  |  |  |  |  | YSSIYAEAKK | 47.95 |  |
|  |  |  |  |  |  | KLLAGYPTIK | 49.54 |  |
|  |  |  |  |  |  | LLAGYPTIK | 68.91 |  |
|  | gi\|58308563 | *M. galloprovincialis* | — | poly-Ala; Low complexity region; Ala (41.2%), Gly ((21.8%), Leu (8.8%) | 239 | AAAAAGASAAAGGSGGTLR | 119.16 | ?FFFFFGYGYGGALDIDLGDLEELLGGLDTIDLEDAAVLSALGLGGGSGLGGGSAAAAAAAAAAAAGGLGGGSAAAAAAAAAAAAGGLGGGSAAAAAAAAAAAGGAGGIGGSSAAAAAAAAAAAGRRAAAAAGASAAAGGSGGTLRQRLISRIIARRQSAASAAAAAAASAF? |
|  | gi\|237638533 | *M. coruscus* | — | Low complexity region; Gly (33.4%), Ala (30.8%), Leu (6.2%) | 196 | AAAAANAGLGGGSATAAAR | 143.85 | ?RKQLDHEEKTGPDGNQGQRGGTRSTRSYHPCHEDLRTAGVESPVEAFDAGAGPGGAGPGGAGAGAFAGAGPGGAGAGAFGGAGPFGGAGPGGLGGAGPGGLGGAGPGGLGGAGPGGLGGAGPGGLGAGGLGGLGAGLGGLGGLGGLGAGAGGAGGLGAGLGGLGGGAGAAAAAQAAAAANAAGLGGGSAAAAARAAAAANAGLGGGSATAAARAAAAAAANSGLGAGAARAAASAAARATAAGAGRGTAAAAASAAAQAHAATKAQGGSHAHAAAAAHAAASSVIHGGGHGGHGGHGGDYHKPGY* |
|  | gi\|145893971 | *M. californianus* | EP protein precursor [M. edulis] gb\|AAQ63463.1\| 84% | C1Q domain,SM000110 | 138 | AEFDLTSLNADLK | 78.57 | MARYHISFLVLFCVISLFDHGSCNPVEEPHHDEHQDEHHDEHHDAPIVGHHDAFLKAEFDLTSLNADLKKYIHHEIQEEVHEVENHTDHNTHEIDDLHHEIKKLHDEVEYFKSHHVAFSAELTHPIENMVAEEIAHFDKVRVNSGHAYHADTGKFVAPEEGFFYFSVTICTKKDSILEMALHVNDHDEMLIHADAEHLEMGCASNSEIVHLQKGDHVEVVKHGPDGVPPFYVHTMSTFTGFMLH* |
|  |  |  |  |  |  | AEFDLTSLNADLKK | 8.25 |  |
|  |  |  |  |  |  | LHDEVEYFK | 38.54 |  |
|  |  |  |  |  |  |  |  |  |
|  | gi\|212816291 | *M. californianus* | — | Low complexity region; Gly (22.8%), Arg (9.5%), Ser (8.2%) | 129 | YVGSGHVYVER | 68 | MKITICLLFALCSAVSAHGWGGGWGSRGGRGGRGGGWGGGWGGGWGGSRRYVGSGHVYVERQPWWGRYGGRGGGYGGYSGGYGGYGGYGGSSGGGGGGGGYGGYGGYGGYGGYGGYGGYSGYSGGYGGGYGRGGGSGSRKGY* |
|  |  |  |  |  |  |  |  |  |
