# Supplementary material for: Layer-by-Layer Proteomic Analysis of Mytilus galloprovincialis Shell
Source: PLoS One. 2015 Jul 28;10(7):e0133913. doi: 10.1371/journal.pone.0133913 (PMC4517812; doi:10.1371/journal.pone.0133913)
Supplement: S2 Table — (DOCX) [file pone.0133913.s003.docx]

**S2 Table**

| **Fraction** | **Matched EST** | **Organism of matched EST** | **Homology of matched EST[Organism];ID;sequence identity %** | **Domains (ID) or features** | **Protein score** | **Matched peptides** | **Peptide score** | **EST-derived seuqence (the signal peptides are underlined; "?" denotes undetermined amino acids, "*" denotes the stop codon)** |
| --- | --- | --- | --- | --- | --- | --- | --- | --- |
| **N-U-I** | gi\|58308563 | *M. galloprovincialis* | — | poly-Ala containing;Low complexity region; Ala (41.2%), Gly ((21.8%), Leu (8.8%) | 169 | AAAAAGASAAAGGSGGTLR | 86.07 | ?FFFFFGYGYGGALDIDLGDLEELLGGLDTIDLEDAAVLSALGLGGGSGLGGGSAAAAAAAAAAAAGGLGGGSAAAAAAAAAAAAGGLGGGSAAAAAAAAAAAGGAGGIGGSSAAAAAAAAAAAGRRAAAAAGASAAAGGSGGTLRQRLISRIIARRQSAASAAAAAAASAF? |
|  | gi\|212814580 | *M. californianus* | MUTP-3 [M. californianus]sp\|P86859.1\|;100% | — | 106 | VDNGQGIAFR | 69.2 | ?RVHYYLHSCKGRTCYNSERHKIYKMLKGIILIVTIQLVNANFFGVFGKPLYNPFNKDKYMIDFITTFNKLMNMKQPQFPHPKSYPGFPPLFPGIKGKKSVFKTIDFTDMAPGSKKTFRVDNGQGIAFRSKSGNAGGMSFSSGTGGGKGFAFGGTLGGGSNGEFVMSQSGPGLKGGKVTYSKGVPKFAKGLFGMLPFFK* |
| **N-U-II** | gi\|212814580 | *M. californianus* | MUTP-3 [M. californianus]sp\|P86859.1\|; 100% | — | 98 | VDNGQGIAFR | 66.82 | ?RVHYYLHSCKGRTCYNSERHKIYKMLKGIILIVTIQLVNANFFGVFGKPLYNPFNKDKYMIDFITTFNKLMNMKQPQFPHPKSYPGFPPLFPGIKGKKSVFKTIDFTDMAPGSKKTFRVDNGQGIAFRSKSGNAGGMSFSSGTGGGKGFAFGGTLGGGSNGEFVMSQSGPGLKGGKVTYSKGVPKFAKGLFGMLPFFK* |
|  | gi\|145887813 | *M. californianus* | Shell matrix protein [M. californianus] sp\|P86860.1\|; 100% | — | 66 | YGYLAPQYGGLR | 42.51 | ?KGYQYLPVVLKVIAMTTIKNVSEHQAGICVTMSVKLPAILTRQLAQTSCPSLPDPMNRYGYLAPQYGGLRIRACPSGTIYSENQCRYKSNMNGNGGLRGSARKQFRQCSAEFKINFDDGFKDISKGGLAFDYSHISLRRGKGVFVGNSKLYIWGFQSRFLGKTFAIRMKVKIKRGAGKYRPEPIISNCGPNGDSSVEIVVHRGKVIFKAKH? |
|  |  |  |  |  |  | GGLAFDYSHISLR | 38.57 |  |
|  |  |  |  |  |  | LYIWGFQSR | 37.82 |  |
| **N-U-III** | gi\|58306883 | *M. galloprovincialis* | Perlucin-like protein [M. galloprovincialis] sp\|P86854.1\|; 100% | C-type lectin (CTL) or carbohydrate-recognition domain (CRD); SM000034 | 247 | ITDSEENSWVVDMITK | 95.18 | MGKLTVVGILTLFIFYIVAASGKCTAPVNCPAGWKKYKTNCYFFSPDGKNWHDAAKQCQTMGGYLVKITDSEENSWVVDMITKSVKHKYGYWMGMADLKNEGDWRWVNDSSAVSYSNWHRGQPNNANNEDCGHFWSAVNYEWNDIVCNTDQMGYIC* |
|  | gi\|58308563 | *M. galloprovincialis* | — | poly-Ala containing;Low complexity region; Ala (41.2%), Gly ((21.8%), Leu (8.8%) | 223 | AAAAAGASAAAGGSGGTLR | 90.27 | ?FFFFFGYGYGGALDIDLGDLEELLGGLDTIDLEDAAVLSALGLGGGSGLGGGSAAAAAAAAAAAAGGLGGGSAAAAAAAAAAAAGGLGGGSAAAAAAAAAAAGGAGGIGGSSAAAAAAAAAAAGRRAAAAAGASAAAGGSGGTLRQRLISRIIARRQSAASAAAAAAASAF? |
|  | gi\|58307533 | *M. galloprovincialis* | — | No domains; Ser (14.2%), Arg (13.0%), Ala (8.0%), Val (8.0%) | 186 | YSSVPPGYFASTK | 27.54 | ?NKYLAGPSDRSSSSTNEMTVRRSRYSSVPPGYFASTKGHSALKRWSYAPQSRSALYEDTFIPEVIRPRSYYDTSREENDIRRGVNDELVYTSNLMDDTYDVAAKSRSRDQMLLRDAARALVDTETYVSPRSSVTSNRVRATSVVARPAPLTSRAVSCPPTSRRSNQPLYGGKSHWDEEG? |
|  |  |  |  |  |  | SALYEDTFIPEVIRPR | 56.49 |  |
|  |  |  |  |  |  | ALVDTETYVSPR | 86.93 |  |
|  | gi\|212815602 | *M. californianus* | Fibronectin-like protein [M. californianus] sp\|P86861.1\|; 100% | Fibronectin type 3 domain; SM000060 | 162 | QDMFTTAENQLR | 55.86 | MFSFGIILLTVVSFTNAQWRQDMFTTAENQLRSIVQNGQTLLDFIYQERQKHGGGNMTGGSLMSHNVAYSSFINDVETRLADMEQATQELVRIMRTCPDAPLAPPPPTNVIVESTTIDNVSSIVVKWDPPFNPPENMQYKVYFVPVDQNGMQTAGEVVFRICDSTQTIASITDLTPRSRYRIRVGAVAGAVAEGASMPLNVKTP? |
|  |  |  |  |  |  | SIVQNGQTLLDFIYQER | 118.35 |  |
|  | gi\|212817227 | *M. californianus* | — | Low complexity region; Gly (48.6%), Tyr (14.4%), Ser (8.2%) | 111 | YVGSGHVYVER | 72.68 | MKITICLLFVLCSAVSAHGWYGGWGSRGGRGGRGGRGGGWGVGWGGGWGGGRRYVGSGHVYVERQPWWGSYGGRGGGYGGGYGGYSGGYGGYGGYGGSSGGGGGYGGYGGYGGYGGYGGYGGYSGYSGGYGGGYGRGGGSGSRKGY* |
|  | gi\|145893971 | *M. californianus* | EP protein precursor [M. edulis] gb\|AAQ63463.1\|; 84% | C1Q domain,SM000110 | 95 | AEFDLTSLNADLK | 62.91 | MARYHISFLVLFCVISLFDHGSCNPVEEPHHDEHQDEHHDEHHDAPIVGHHDAFLKAEFDLTSLNADLKKYIHHEIQEEVHEVENHTDHNTHEIDDLHHEIKKLHDEVEYFKSHHVAFSAELTHPIENMVAEEIAHFDKVRVNSGHAYHADTGKFVAPEEGFFYFSVTICTKKDSILEMALHVNDHDEMLIHADAEHLEMGCASNSEIVHLQKGDHVEVVKHGPDGVPPFYVHTMSTFTGFMLH* |
|  |  |  |  |  |  | KLHDEVEYFK | 31.11 |  |
|  |  |  |  |  |  | LHDEVEYFK | 38.78 |  |
|  | gi\|223021659 | *M. galloprovincialis* | MUSP-1[M. galloprovincialis] sp\|P86853.1\|;100% | — | 86 | DRYSSIYAEAK | 53.63 | MISKYCLFVIVLGTTGTALVLTNDSNKLQNVKAVIAIQDKVLHFHDHTTDCVGELMCIFAALPESERNQTLSIPLGLLTTIATDKGRDRYSSIYAEAKKLLAGYPTIKHALNAAENGHSTKDKNVCASMYSKCPFEPDDLLDTINDLEDITTLFSKNVFGKVIADAIEYNYTQVGMTQTHS? |
|  |  |  |  |  |  | YSSIYAEAK | 41.11 |  |
|  |  |  |  |  |  | KLLAGYPTIK | 53.73 |  |
|  |  |  |  |  |  | LLAGYPTIK | 39.13 |  |
|  |  |  |  |  |  |  |  |  |
| **M-U-I** | gi\|145900307 | *M. californianus* | Whirlin [Crassostrea gigas] gb\|EKC33408.1\|61% | PDZ domain; SM000228 | 208 | DPSVQWGFR | 66.85 | MSEDQPVFVQLRRKDPSVQWGFRMNGGRDQGSILYIQKLSRNGVGDRSGLRPGDGILKINNVPATYLDHEQAKMEIIRSGNELDFVVQRDAIDVGQHKGAQTKPAPKQRSEVDEEPSMYKGYTNPNVQSRSFKILQQSLNYSEAPQE* |
|  |  |  |  |  |  | DQGSILYIQK | 46.6 |  |
|  |  |  |  |  |  | SGLRPGDGILK | 26.99 |  |
|  |  |  |  |  |  | INNVPATYLDHEQAK | 65.16 |  |
|  |  |  |  |  |  | SEVDEEPSMYK | 39.86 |  |
|  | gi\|58308563 | *M. galloprovincialis* | — | poly-Ala containing;Low complexity region; Ala (41.2%), Gly ((21.8%), Leu (8.8%) | 200 | AAAAAGASAAAGGSGGTLR | 92.61 | ?FFFFFGYGYGGALDIDLGDLEELLGGLDTIDLEDAAVLSALGLGGGSGLGGGSAAAAAAAAAAAAGGLGGGSAAAAAAAAAAAAGGLGGGSAAAAAAAAAAAGGAGGIGGSSAAAAAAAAAAAGRRAAAAAGASAAAGGSGGTLRQRLISRIIARRQSAASAAAAAAASAF? |
|  | gi\|58308196 | *M. galloprovincialis* | calponin-like protein [M. galloprovincialis] dbj\|BAB60813.1\| 94% | 3 Calponin domain; PF00402 | 158 | NLPMVLATISHVGTEAQR | 108.82 | ?KFLKAAHKYGVPNTSLFQTVELYEARNLPMVLATISHVGTEAQRHNYQGPTIGSKPTEKHRVQFSYEQLKQSHGTIGLQSGTNKFATQKGMRIGSVRHISDIKVEDLDREGNTLLTLQAGTNRFASQKGMTGFGAVRHIADIRADQFDKDGENIITLQAGTNKFASQ? |
|  |  |  |  |  |  | VQFSYEQLK | 25.12 |  |
|  |  |  |  |  |  | QSHGTIGLQSGTNK | 58.99 |  |
|  |  |  |  |  |  | EGNTLLTLQAGTNR | 35.54 |  |
|  | gi\|212830099 | *M. californianus* | — | Low complexity region; Leu (11.3%), Gly (9.0%), Glu (8.3%), Lys (8.3%) | 148 | YNPLSQNEVQYYLQELQELK | 89.84 | MNKNIISLGLCMCSILLLTGADRQKRSIFSVKRYNPLSQNEVQYYLQELQELKERLNGGHGGKSKREKENLKKEVRNRHPGEKSRHGEQACPGGIAGMAKRPEVLQVHGHHHHHLINVTNQLHTEIQTVPPIL* |
|  | gi\|223023515 | *M. galloprovincialis* | calponin-like protein [M. galloprovincialis] dbj\|BAB60813.1\|; 94% | Calponin homology domain (SM000033) | 117 | NLPMVLATISHVGTEAQR | 108.82 | ?MADRVKPMGMDRALISKMGAKYDSGLEYEVRGWIKALIGEDIGEGPSNVEKSLRDGVILCTLMKKVIEGTPSESLPAACSKTDLKSSASELPFKQMENIEKFLKAAHKYGVPNTSLFQTVELYEARNLPMVLATISHVGTEAQRLNFNGETIGSKPTVKHDVTFSYEQLKQSCGLIG? |
|  |  |  |  |  |  | HDVTFSYEQLK | 29.65 |  |
|  | gi\|238643545 | *M. galloprovincialis* | NSPI2; Flags: Precursor [Pinctada maxima]sp\|P86964.1\|; 32% | 2 KU domain; SM000131 | 106 | IPMYYYSSQSGR | 60.55 | MLRLLILLCMPVLYAQINFQQILAWKQLPRRCRIPPYTGTSPLSHMIKIPMYYYSSQSGRCESFNYSGLGKSKNMFRNPIDCLRKCACYAPMDAGTCTNSTTGTTRYYYNRRFKMCTTFQFSGCEGNDNNFSDYVSCHIACNRRRAEDL* |
| **M-U-II** | gi\|58308563 | *M. galloprovincialis* | — | — | 164 | AAAAAGASAAAGGSGGTLR | 98.92 | FFFFFGYGYGGALDIDLGDLEELLGGLDTIDLEDAAVLSALGLGGGSGLGGGSAAAAAAAAAAAAGGLGGGSAAAAAAAAAAAAGGLGGGSAAAAAAAAAAAGGAGGIGGSSAAAAAAAAAAAGRRAAAAAGASAAAGGSGGTLRQRLISRIIARRQSAASAAAAAAASAF? |
| **M-U-III** | gi\|58307533 | *M. galloprovincialis* | — | No domains; Ser (14.2%), Arg (13.0%), Ala (8.0%), Val (8.0%) | 144 | ALVDTETYVSPR | 86 | ?NKYLAGPSDRSSSSTNEMTVRRSRYSSVPPGYFASTKGHSALKRWSYAPQSRSALYEDTFIPEVIRPRSYYDTSREENDIRRGVNDELVYTSNLMDDTYDVAAKSRSRDQMLLRDAARALVDTETYVSPRSSVTSNRVRATSVVARPAPLTSRAVSCPPTSRRSNQPLYGGKSHWDEEG? |
|  |  |  |  |  |  | YSSVPPGYFASTK | 20.58 |  |
|  |  |  |  |  |  |  |  |  |
| **FP-U-I** | gi\|58308563 | *M. galloprovincialis* | — | poly-Ala containing;Low complexity region; Ala (41.2%), Gly ((21.8%), Leu (8.8%) | 283 | AAAAAGASAAAGGSGGTLR | 130.39 | ?FFFFFGYGYGGALDIDLGDLEELLGGLDTIDLEDAAVLSALGLGGGSGLGGGSAAAAAAAAAAAAGGLGGGSAAAAAAAAAAAAGGLGGGSAAAAAAAAAAAGGAGGIGGSSAAAAAAAAAAAGRRAAAAAGASAAAGGSGGTLRQRLISRIIARRQSAASAAAAAAASAF? |
|  | gi\|58306883 | *M. galloprovincialis* | Perlucin-like protein [M. galloprovincialis] sp\|P86854.1\|; 100% | C-type lectin (CTL) or carbohydrate-recognition domain (CRD); SM000034 | 212 | ITDSEENSWVVDMITK | 129.57 | MGKLTVVGILTLFIFYIVAASGKCTAPVNCPAGWKKYKTNCYFFSPDGKNWHDAAKQCQTMGGYLVKITDSEENSWVVDMITKSVKHKYGYWMGMADLKNEGDWRWVNDSSAVSYSNWHRGQPNNANNEDCGHFWSAVNYEWNDIVCNTDQMGYIC* |
| **FP-U-II** | gi\|58306883 | *M. galloprovincialis* | Perlucin-like protein [M. galloprovincialis] sp\|P86854.1\|; 100% | C-type lectin (CTL) or carbohydrate-recognition domain (CRD); SM000034 | 279 | ITDSEENSWVVDMITK | 101.31 | MGKLTVVGILTLFIFYIVAASGKCTAPVNCPAGWKKYKTNCYFFSPDGKNWHDAAKQCQTMGGYLVKITDSEENSWVVDMITKSVKHKYGYWMGMADLKNEGDWRWVNDSSAVSYSNWHRGQPNNANNEDCGHFWSAVNYEWNDIVCNTDQMGYIC* |
|  | gi\|58307710 | *M. galloprovincialis* | precollagen D [M. edulis] gb\|AAB96638.1\| 100% | 2 Collagen domain PF01391 | 150 | GSVGDQGAQGDQGATGADGK | 150.01 | ?FAPGPKGSVGDQGAQGDQGATGADGKKGEPGERGQQGAAGPVGRPGPRGDRGAKGIQGSRGRPGGMGRRGNRGSQGAVGPRGETGPDGNQGQRGEQGAPGVITLVIEDLRTAGVESPD* |
|  | gi\|212814580 | *M. californianus* | MUTP-3 [M. californianus]sp\|P86859.1\|; 100% | — | 116 | VDNGQGIAFR | 85.41 | ?RVHYYLHSCKGRTCYNSERHKIYKMLKGIILIVTIQLVNANFFGVFGKPLYNPFNKDKYMIDFITTFNKLMNMKQPQFPHPKSYPGFPPLFPGIKGKKSVFKTIDFTDMAPGSKKTFRVDNGQGIAFRSKSGNAGGMSFSSGTGGGKGFAFGGTLGGGSNGEFVMSQSGPGLKGGKVTYSKGVPKFAKGLFGMLPFFK* |
|  | gi\|58308563 | *M. galloprovincialis* | — | poly-Ala containing;Low complexity region; Ala (41.2%), Gly ((21.8%), Leu (8.8%) | 115 | AAAAAGASAAAGGSGGTLR | 78.29 | ?FFFFFGYGYGGALDIDLGDLEELLGGLDTIDLEDAAVLSALGLGGGSGLGGGSAAAAAAAAAAAAGGLGGGSAAAAAAAAAAAAGGLGGGSAAAAAAAAAAAGGAGGIGGSSAAAAAAAAAAAGRRAAAAAGASAAAGGSGGTLRQRLISRIIARRQSAASAAAAAAASAF? |
|  | gi\|212812207 | *M. californianus* | — | 9 Transmembrane region Phe (23.9%), Gly (17.8%), Val (17.4%) | 112 | YVGSGHVYVER | 71.85 | ?RGSRRYVGSGHVYVERQPWWGSYGGRGGGYGGGYGGYSGGYGGYGGYGGYGGCGGXGGXXGGGGGFVVFGGFFGCCCFCVVCVFLFXLCVCFLFFCGVCWLGCFCCFVCGVFWGFLGVVVFWFFLLLGVFFVLLCLCFLVVGVVFFFLCWFVFWWVCLCLFGFFCFVVFXVVVVLCFCFFFWFLCFVLLVFLWFXCWWLVFFXLXCFFVVVVFWFVGCVLFVFFFGLFVFLVLFLVXFFLFVVCFGVFLFFFLWXFCLCFFFVC? |
|  | gi\|145893971 | *M. californianus* | EP protein precursor [M. edulis] gb\|AAQ63463.1\|; 84% | C1Q domain,SM000110 | 90 | AEFDLTSLNADLK | 58.3 | MARYHISFLVLFCVISLFDHGSCNPVEEPHHDEHQDEHHDEHHDAPIVGHHDAFLKAEFDLTSLNADLKKYIHHEIQEEVHEVENHTDHNTHEIDDLHHEIKKLHDEVEYFKSHHVAFSAELTHPIENMVAEEIAHFDKVRVNSGHAYHADTGKFVAPEEGFFYFSVTICTKKDSILEMALHVNDHDEMLIHADAEHLEMGCASNSEIVHLQKGDHVEVVKHGPDGVPPFYVHTMSTFTGFMLH* |
|  |  |  |  |  |  | LHDEVEYFK | 32.49 |  |
