# Supplementary material for: Layer-by-Layer Proteomic Analysis of Mytilus galloprovincialis Shell
Source: PLoS One. 2015 Jul 28;10(7):e0133913. doi: 10.1371/journal.pone.0133913 (PMC4517812; doi:10.1371/journal.pone.0133913)
Supplement: S3 Table — (DOCX) [file pone.0133913.s004.docx]

**S3 Table**

| **Matched EST** | **Organism** | **Homologue ID** | **Homologue name / organism** | **Identity%** | **E-value** | **Protein score** | **Matched peptides** | **Matched Sequence (score)** | **Domains or features** | **EST-derived seuqence (the signal peptides are underlined; "?" denotes undetermined amino acids, "*" denotes the stop codon)** |
| --- | --- | --- | --- | --- | --- | --- | --- | --- | --- | --- |
| gi 212816630 | *M.californianus* | — | — | — | — | 1394 | 3 | SAAKIIIQLLTR(53); IIIQLLTR(66);IIILAADR(32) | Gly(27.8%);Ala(20.6%);Ser(20.6%);Asp(13.5%) | ?GGAGGGSGAAASAAAAAAAGGRSGLIRWLVARRAAARAAASAGAGAGGIAVGAGGAGGAGGAGGAGAGGAGGAGGSGGSGGSGDGGGDGDCGSSDSDSGSDSDSDNDTDSSDSDTGSDASNSDSGSDSDGDGDSDSSGTSESSSSDSGDTSNEDSDDGSDDDDDDSFRSAAKIIIQLLTRLLMSGGFAGAGSSASASASAAASAGGGAGGAGLGLGGGSGAAS? |
| gi\|58308196 | *M.galloprovincialis* | gi\|14422379\| | calponin-like protein / M. galloprovincialis | 100 | 9.00E-117 | 460 | 6 | EGNTLLTLQAGTNR(97);HNYQGPTIGSKPTEK(45);NLPMVLATISHVGTEAQR(90);YGVPNTSLFQTVELYEAR(95);ADQFDKDGENIITLQAGTNK(86);GMTGFGAVR(52) | Calponin domain(PF00402) | MVLATISHVGTEAQRHNYQGPTIGSKPTEKHRVQFSYEQLKQSHGTIGLQSGTNKFATQKGMRIGSVRHISDIKVEDLDREGNTLLTLQAGTNRFASQKGMTGFGAVRHIADIRADQFDKDGENIITLQAGTNKFASQ? |
| gi 58308563 | *M.galloprovincialis* | — | — | — | — |  | 3 | AAAAAGASAAAGGSGGTLR(136);LISRIIAR(30);RAAAAAGASAAAGGSGGTLR(106) | Low complexity region; Ala (41.2%), Gly ((21.8%), Leu (8.8%) | ?FFFFFGYGYGGALDIDLGDLEELLGGLDTIDLEDAAVLSALGLGGGSGLGGGSAAAAAAAAAAAAGGLGGGSAAAAAAAAAAAAGGLGGGSAAAAAAAAAAAGGAGGIGGSSAAAAAAAAAAAGRRAAAAAGASAAGGSGGTLRQRLISRIIARRQSAASAAAAAAASAF? |
| gi\|223023515 | *M.galloprovincialis* | gi\|14422379\| | calponin-like protein / M. galloprovincialis | 93.79 | 2.00E-114 | 365 | 4 | HDVTFSYEQLK(58);ALIGEDIGEGPSNVE(91);NLPMVLATISHVGTEAQR(90);YGVPNTSLFQTVELYEAR(95) | Calponin domain(PF00402) | MADRVKPMGMDRALISKMGAKYDSGLEYEVRGWIKALIGEDIGEGPSNVEKSLRDGVILCTLMKKVIEGTPSESLPAACSKTDLKSSASELPFKQMENIEKFLKAAHKYGVPNTSLFQTVELYEARNLPMVLATISHVGTEAQRLNFNGETIGSKPTVKHDVTFSYEQLKQSCGLIG? |
| gi\|223026184 | *M.galloprovincialis* | gi\|14422379\| | calponin-like protein / M. galloprovincialis | 98.19 | 4.00E-157 | 337 | 5 | TDLKPSSSELPFK(34);HNYQGPTIGSKPTEK(45);NLPMVLATISHVGTEAQR(90);YGVPNTSLFQTVELYEAR(95) | Calponin domain(PF00402) | MKKVIEGTPSESLPAACSKTDLKPSSSELPFKQMENIEKFLKAAHKYGVPNTSLFQTVELYEARNLPMVLATISHVGTEAQRHNYQGPTIGSKPTEKHIVQFSYEQLKQSHGTIGLQSGTNKFATQKGMRIGSVRHISDIKVEDLDKEGNTLLALQAGTNRFASQKGMTGFGAVRHIADIRADEFDKDGENIITLQAGTNKFAS? |
| gi\|58307858 | *M.galloprovincialis* | gi\|390979787\| | distal byssal thread collagen / synthetic construct | 90.64 | 2.00E-24 | 325 | 3 | GPVGGQGPAGPAGPLGPQGPMGER(100);GSVGDQGAQGDQGATGADGK(99);GPDGETGPQGPAGPK(69) | Internal repeats; Gly (33.9%), Pro (14.4%), Gln (10.3%) | ?TRTQGPTGSEGPVGAPGPKGSVGDQGAQGDQGATGADGKPGDRGPDGETGPQGPAGPKGQVGDQGKPGAKGETGDQGARGEAGKAGEQGPGGIQGPKGPVGGQGPAGPAGPLGPQGPMGERGPQGPTGSEGPVGAPGPKGSVGDQGAQGDQGATGADGKKGEPGERGQQGAAGP? |
| gi\|212823360 | *M.californianus* | gi\|14422379\| | calponin-like protein / M. galloprovincialis | 96.06 | 6.00E-140 | 313 | 3 | HNYQGPTIGSKPTEK(45);NLPMVLATISHVGTEAQ(90);YGVPNTSLFQTVELYEAR(95) | Calponin domain(PF00402) | MADRVKPMGMDRALISKMGAKYDSGLEYEVRGWIKQLIGEDIGEGPSNVEKSLRDGVILCNLMKKVIDGTPSESLPAACAKTDLKSSPSELPFKQMENIEKFLKAAHKYGVPNTSLFQTVELYEARNLPMVLATISHVGTEAQRHNYQGPTIGSKPTEKHQVQFSYEQLKQSHGTIGLQSGTNKFATQKGMRIGSIRHISDIK |
| gi\|223026183 | *M.galloprovincialis* | gi\|14422379\| | calponin-like protein / M. galloprovincialis | 96.11 | 2.00E-179 | 295 | 3 | EAQSVIPLQYGTNR(49);HNYQGPTIGSKPTEK(45);YGVPNTSLFQTVELYEAR(95) | Calponin domain(PF00402) | MVLATISHVGTEVQRHNYQGPTIGSKPTEKHRVQFSYEQPKQSHGTIGLQSGTNKFATQKGMRIGSVRHISDIKVEDLDREGNTPLTLQAGTNRFASQKGMTGFGSVRHIADIRADQMDKEGENIITLQAGTNKFASQKGMTGFGAVRHVSDIRADDCDPQTSLHIGLQAGSNQFASQKGMTSMGAVRHICDIRADDLDREAQSVIPLQYGTNRGSNQKGMTSFGS |
| gi\|145896743 | *M.californianus* | gi\|14422379\| | calponin-like protein / M. galloprovincialis | 82.02 | 1.00E-155 | 277 | 2 | NLPMVLATISHVGTEAQR(90);YGVPNTSLFQTVELYEAR(95) | Calponin domain(PF00402) | MADRVKPMGMDRALISKMGAKYDSGLEYEVRGWIKQLIGEDIGEGPSNVEKSLRDGVILCNLMKKVIDGTPSESLPAACAKTDLKSSPSELPFKQMENIEKFLKAAHKYGVPNTSLFQTVELYEARNLPMVLATISHVGTEAQRLNFNGETIGSKPTVKHDVNFSYEQLKQSCGLIGLQSGTNKFASQKGMRIGAVRHIADIRAE. |
| gi\|212814945 | *M.californianus* | gi\|14422379\| | calponin-like protein / M. galloprovincialis | 91.26 | 8.00E-115 | 277 | 2 | NLPMVLATISHVGTEAQR(90);YGVPNTSLFQTVELYEAR(95) | Calponin domain(PF00402) | MADRVKPMGMDRALISKMGAKYDSGLEYEVRGWIKQLIGEDIGEGPSNVEKSLRDGAILCNLMKKVIDGTPSESLPAACAKTDLKSSPSELPFKQMENIEKFLKAAHKYGVPNTSLFQTVELYEARNLPMVLATISHVGTEAQRLNFNGETIGSKPTVKHDVNFSYEQLKQSCGLIGLQSGTN |
| gi\|223027721 | *M.galloprovincialis* | gi\|405967947\| | Transgelin-2 / C. gigas | 59.76 | 5.00E-61 | 272 | 5 | SPVNFQK(38);LINILLK(40);GIQDYGVDR(47);ESEFQSGDLWEVR(36);VMSPFVAMTNIENFNK(71) | Calponin homology domain (SM000033) | MSGRASKSGIGLKVEKKLEENYDREEAAGTPTHVVNWVNAILGSEHDPIPGTDWKSICNHLRDGVALCKLINILLKKDGKSPVNFQKKVMSPFVAMTNIENFNKGIQDYGVDRESEFQSGDLWEVRKGPFLNVINCISSLGFVANKKGVTPKYTGEIRKYLDNE. |
| gi\|58307336 | *M.galloprovincialis* | gi\|21105303\| | precollagen-D / M. galloprovincialis | 98.95 | 6.00E-20 | 266 | 3 | GDEGPVGPK(30);QGPVGGQGPAGPR(40);AGEQGPGGIQGP(32)GPDGETGPQGPAGPK(69) | Collagen domain (PF01391); Internal repeat | ?SPDPRTTGPDGAMGPQGPCGDRGAPGVPGKQGPVGGQGPAGPRGPRGDEGPVGPKGEPGAKGADGKPGDRGPDGETGPQGPAGPKGQVGDQGKPGAKGETGDQGARGEAGKAGEQGPGGIQGPKGPVGGQGPAGPAGPLGPQGPMGERGPQGRTPAGTPGPPGNPGEPGQGGAPGAPG? |
| gi\|212831833 | *M.californianus* | gi\|322518586\| | Fibronectin-like protein / M. californianus | 100 | 1.00E-141 | 262 | 3 | LADMEQATQELVR(71);VGAVAGAVAEGASMPLNVK(57);SIVQNGQTLLDFIYQER(95) | Fibronectin type 3 domain (SM000060) | MFSFGIILLTVVSFTNAQWRQDMFTTAENQLRSIVQNGQTLLDFIYQERQKHGGGNMTGGSLMSHNVAYSSFINDVETRLADMEQATQELVRIMRTCPDAPLAPPPPTNVIVESTTIDNVSSIVVKWDPPFNPPENMQYKVYFVPVDQNGMQTAGEVVFRICDSTQTIASITDLTPRSRYRIRVGAVAGAVAEGASMPLNVKTPDIIPSRVRNVMVKSSTANTI? |
| gi\|58308539 | *M.galloprovincialis* | gi\|524882698\| | collagen alpha-4(VI) chain-like / A. californica | 38 | 4.00E-19 | 254 | 3 | EIAEILYDR(49);ADFDSALLELR(65);ELDFVGEVITAFDLGK(92) | von Willebrand factor type A (VWA) domain (SM000327) | MLFLSLFSLLLIARVTARSPPAPPEPPVYRRCLKKIADVFFVVDTSSSLDITPNVIKELDFVGEVITAFDLGKDQVRTGMMTFATNTELLFKLDDFKTKKEIAEILYDRKNLVKYRWKGGNTNIGKALRLLMDGGLSTSHGSRADVPQIAVIITDGNSNDRADFDSALLELRKKNLIVFA? |
| gi\|212819041 | *M.californianus* | gi\|390979785\| | procollagen-proline dioxygenase beta subunit / M. galloprovincialis | 97.21 | 7.00E-142 | 252 | 2 | NNFEGEVTADAVNK(116);AAQGIDDIPFGITSNTDIFKEYEMESDGVALFK(76) | Thioredoxin_6 domain(PF13848) | MIEKDEVVVLGFFKDLKSESAKEYEKAAQGIDDIPFGITSNTDIFKEYEMESDGVALFKKFDEGRNNFEGEVTADAVNKFISANRLPLIIEFTQESAQKIFGGEIKNHILLFLEKKADASAKILEGYRKAAVGFKGKVLFITLDTSDEDNARILEFFGLKKEETPAARLITLGEDMTKYKPDSDDLSEDAVTSFVQSFLDGKLKAHLMS? |
| gi\|223026852 | *M.galloprovincialis* | gi\|405967947\| | Transgelin-2 / C. gigas | 62.73 | 2.00E-62 | 233 | 3 | LINILLK(40);ESEFQSGDLWEVR(36);VMSPFVAMTNIENFNK(71) | Calponin homology domain (SM000033) | MSGSGRANKSGLGYEVEKKMEANYDREEAAGTPTHVVNWVNAILGSEHDPIPGTDWKSICNHLRDGVALCKLINILLKKDGKSPINFQKKVMSPFVAMTNIENFNKGIQDYGVDKESEFQSGDLWEVRKGPFLNVINCISSLGFVANKKGATPKYTGEIRKYLDNE* |
| gi\|145887968 | *M.californianus* | gi\|322966920\| | Shell matrix protein / M. californianus | 100 | 2.00E-171 | 229 | 5 | TGGNLEIR(73);INFDDGFK(55);LYIWGFQSR(52);INFDDGFKDISK(79);GGLAFDYSHISLR(56) | Laminin_G_3 domain (PF13385) | MNGNGGLRGSARKQFRQCSAEFKINFDDGFKDISKGGLAFDYSHISLRRGKGVFVGNSKLYIWGFQSRFLGKTFAIRMKVKIKRGAGKYRPEPIISNCGPNGDSSVEIVVHRGKVIFKAKTSDNPEAVFITEDYDDDKWTDLTYYYDGNHFGGSCNGRPFRQRTGGNLEIRDNPMTIGLCTGQNGFHGEIDELEIYTACIPKDM* |
| gi\|58308904 | *M.galloprovincialis* | gi\|558208493\| | acidic mammalian chitinase-like / Pelodiscus sinensis | 56.32 | 5.00E-54 | 226 | 4 | IVLGISTYGR(57);EFENEAQSTG(58);TLNQDWAVQYWIDNGTPK(85);IIADLDFINLMAYDLHGSWER(40) | Glyco_18 domain (SM000636) | MAYDLHGSWERKTGHISPLYPRKDETGAERTLNQDWAVQYWIDNGTPKEKIVLGISTYGRTFKLSSSSNNGFGAATAGGGSPGKNTGESGFLSYYEICSSGWTTVWNDEHKVPYAYSGDQWVGYDXCSKCYD* |
| gi\|58306536 | *M.galloprovincialis* | gi\|524882669\| | collagen alpha-1(XII) chain-like / A. californica | 40.19 | 1.00E-12 | 223 | 2 | EIAEILYDR(49)ELDFVGEVITAFDLGK(92) | von Willebrand factor type A (VWA) domain (SM000327) | MFSLLLIHPVDLDITPNVIKELDFVGEVITAFDLGKDQVRTGMMTFATNTELLFKLDDFKTKKEIAEILYDRKNLVKYPWKGGNTNIGKALTLLMDGGLSTSHGSRADVPQIAVIITDGNSNDRADFDS? |
| gi\|37650124 | *M.galloprovincialis* | gi\|405963175\| | 60 kDa neurofilament protein / C. gigas | 87.97 | 2.00E-72 | 207 | 7 | VHEQELK(31);SELSQAIR(36);ELAALAYR(44);GDMESYYNLK(63);DTTEENREFWK(39);DIQSEYDNKVDQLR(59);IDLNNETLNHLDAENR(62) | Filament domain (PF00038) | MEFLKKVHEQELKELAALAYRDTTEENREFWKSELSQAIRDIQSEYDNKVDQLRGDMESYYNLKVQEFRTGATKQNMEVTHVKEENKKLVK? |
| gi\|58308122 | *M.galloprovincialis* | gi\|670984547\| | chitotriosidase-1 / Ursus maritimus | 57.3 | 4.00E-56 | 205 | 4 | LVLGISTYGR(57);VPYAYSGDQWVGYDNV(94);TLNQDWAVQYWIDNGTPK(85);IIADLDFINLMAYDLHGSWER(40) | Glyco_18 domain (SM000636) | MAYDLHGSWERKTGHISPLYPRKDETGAERTLNQDWAVQYWIDNGTPKEKLVLGISTYGRTFKLSSSSNNGFGAATAGGGSPGKNTGESGFLSYYEICSSGWTTVWNDEHKVPYAYSGDQWVGYDNVRSVTIKAQYIKEKGLGGAMFWALD |
| gi\|238643094 | *M.galloprovincialis* | gi\|34304719\| | EP protein precursor / M. edulis | 98.91 | 3.00E-116 | 204 | 3 | HLHEEVEYFK(52);VNSGHAYHADTGK(33);SHHVAFSAELTHPIENIAAEEIAHFDK(74) | Complement component C1q domain (SM000110) | ?LTSLNADLEKFIHHEIEKEIHDVENHTEHNKHEIDELHQEIKHLHEEVEYFKSHHVAFSAELTHPIENIAAEEIAHFDKVRVNSGHAYHADTGKFVAPEEGFFYFSVTICTERDSILEMALHVNDHDEMIIHADAEHLELGCASNSEIVQLQKGDHVEVVKHGADGVPPFYIHTMSTFTGFMLH? |
| gi\|238643096 | *M.galloprovincialis* | gi\|325504311\| | putative C1q domain containing protein MgC1q6 / M. galloprovincialis | 100 | 4.00E-142 | 204 | 3 | HLHEEVEYFK(52);VNSGHAYHADTGK(33);SHHVAFSAELTHPIENIAAEEIAHFDK(74) | Complement component C1q domain (SM000110) | MGRYQISLLVLFCVVSLFDQGLTNPVDDHHDAPIVGHHDAFLKAEFDLTSLNADLEKFIHHEIEKEIHDVENHTEHNKHEIDELHQEIKHLHEEVEYFKSHHVAFSAELTHPIENIAAEEIAHFDKVRVNSGHAYHADTGKFVAPEEGFFYFSVTICTKRDSILEMALHVNDHDEMIIHADAEHLELGCASNSEIVHLQKGDHVEVVKHGADGVPPFYIHTMSTFTGFM? |
| gi\|145887813 | *M.californianus* | gi\|322966920\| | Shell matrix protein / M. californianus | 100 | 4.00E-150 | 200 | 4 | INFDDGFK(55);LYIWGFQSR(52);INFDDGFKDISK(79);GGLAFDYSHISLR(62) | No domains; Gly (11.2%), Lys (8.6%), Ser (8.6%) | MTTIKNVSEHQAGICVTMSVKLPAILTRQLAQTSCPSLPDPMNRYGYLAPQYGGLRIRACPSGTIYSENQCRYKSNMNGNGGLRGSARKQFRQCSAEFKINFDDGFKDISKGGLAFDYSHISLRRGKGVFVGNSKLYIWGFQSRFLGKTFAIRMKVKIKRGAGKYRPEPIISNCGPNGDSSVEIVVHRGKVIFKAKH? |
| gi\|223021924 | *M.galloprovincialis* | — | — | — | — | 191 | 4 | GMGMMEGGMGGGK(42);GMGMPMMMPEMGGK(41);MMMMMPEMGGK(49),GMGMPMMMMMPEMGGK(35) | Low complexity region; Met (42.3%), Gly (28.2%),Lys (10.6%) | ?WKKMMMMMPEMGGKGMGMMMPEMGGKGMGMMMPEMGGKGMGMMMPEMGGKGMGVMMPEMGGKGMGMMMPEMGGKGMGMMMPEMGGKGMGMMEKGMGMPMMMPEMGGKGMGMMEKMMMMMPEMGGKGMGMMKKGMGMPIMMPE? |
| gi\|58306567 | *M.galloprovincialis* | gi\|584064561\| | chitotriosidase-1-like / Myotis davidii | 53.37 | 8.00E-55 | 184 | 3 | IVLGISTYGR(57);VPYAYSGDQWVGYDNVR(94);TLNQDWAVQYWIDNGTPK(85) | Low complexity region; Gly (13.0%), Ser (9.0%), Thr (7.9%) | YDLHGSWERKTGHISPLYPRKDETGAERTLNQDWAVQYWIDNGTPKEKIVLGISTYGRTFKLSSSSNNGFGAATAGGGSPGKNTGESGFLSYYEICSSGWTTVWNDEHKVPYAYSGDQWVGYDNVRSVTIKAQYIKEKGLGGAMFWALDLDDFTGNACHEGDYPLISAVTNELKTSG |
| gi\|223026111 | *M.galloprovincialis* | gi\|14422379\| | calponin-like protein / M. galloprovincialis | 99.04 | 2.00E-148 | 179 | 4 | VSDLAEDMKR(65);EGNTLLTLQAGTNR(97);EAQSVIPLQYGTNR(49);GMTGFGAVR(52) | Calponin domain(PF00402) | MTGFGAVRHIADIRADEFDKDGENIITLQAGTNKFASQKGMTGFGAVRHVSDIRADDFDPQTASHIGLQAGSNQFASQKGMTSMGAVRHICDIRADDLDREAQSVIPLQYGTNRGSSQKGMTSFGSQRHIADIKVSDLAEDMKRQDLNMTPKEYQEFRQQMEATEQKTDEPQYE. |
| gi\|223021659 | *M.galloprovincialis* | gi\|322518387\| | MUSP-1 / M. galloprovincialis | 100 | 9.00E-120 | 153 | 5 | AVIAIQDK(44);LLAGYPTIK(43);YSSIYAEAK(56);DRYSSIYAEAK(51);VIADAIEYNYTQVGMTQTHS(68) | No domains | MISKYCLFVIVLGTTGTALVLTNDSNKLQNVKAVIAIQDKVLHFHDHTTDCVGELMCIFAALPESERNQTLSIPLGLLTTIATDKGRDRYSSIYAEAKKLLAGYPTIKHALNAAENGHSTKDKNVCASMYSKCPFEPDDLLDTINDLEDITTLFSKNVFGKVIADAIEYNYTQVGMTQTHS? |
| gi\|145893257 | *M.californianus* | — | — | — | — | 152 | 2 | AQSLIDEAEQR(68);KAQSLIDEAEQR(95) | No domains;Arg (10.6%), Lys (9.6%), Ser (9.6%) | ?LKKRRQVPSSRIPVIPMNPDLQGMLKRRRRSNITWNNIRKAQSLIDEAEQRANMGKRTCKPYADHDPCCFTGGNQSSQGLNSYFKKKDFIESSF* |
| gi\|58307533 | *M.galloprovincialis* | — | — | — | — | 148 | 4 | SSSSTNEMTVR(44);ALVDTETYVSPR(47);SALYEDTFIPEVIRPR(44);GVNDELVYTSNLMDDTYDVAAK(77) | No domains; Ser (14.2%), Arg (13.0%), Ala (8.0%), Val (8.0%) | MTVRRSRYSSVPPGYFASTKGHSALKRWSYAPQSRSALYEDTFIPEVIRPRSYYDTSREENDIRRGVNDELVYTSNLMDDTYDVAAKSRSRDQMLLRDAARALVDTETYVSPRSSVTSNRVRATSVVARPAPLTSRAVSCPPTSRRSNQPLYGGKSHWDEEG? |
| gi\|238641522 | *M.galloprovincialis* | gi\|405971603\| | Cathepsin L / C. gigas | 53.15 | 7.00E-75 | 147 | 3 | YFDIQPASVK(53);GHVVTSFEPYDK(47);FLPPLNAVLPENVDWR(57) | Cathepsin propeptide inhibitor domain (SM000848);Papain family cysteine protease (Pept_C1) domain(SM000645) | MLLLCILLVAIATVFTAPQTQDQNSIIRYFDIQPASVKMTVTAPKGHVVTSFEPYDKSWEKFKLEHSKSYHTIEEETYRRTVFKKNALKIEEHNKQYSLGQKSYYLGINQFADLEHWEYMQHHGFQVKKTVNRTRTGSKFLPPLNAVLPENVDWRDKGYVTPVKNQGQCGSCWSFSTTGALEGQHFRKSGKLLSLSEQQLVDCSGDYGNEGCNGGLMDDAFKYI? |
| gi\|58307710 | *M.galloprovincialis* | gi\|21105303\| | precollagen-D / M. galloprovincialis | 91.3 | 7.00E-15 | 139 | 2 | NAMQSQLDEMR(55);LTGELEDLGIDVER(94) | Collagen domain (PF01391) | ?FAPGPKGSVGDQGAQGDQGATGADGKKGEPGERGQQGAAGPVGRPGPRGDRGAKGIQGSRGRPGGMGRRGNRGSQGAVGPRGETGPDGNQGQRGEQGAPGVITLVIEDLRTAGVESPD* |
| gi\|238644365 | *M.galloprovincialis* | gi\|322966877\| | Perlucin-like protein / M. galloprovincialis | 81.58 | 3.00E-88 | 138 | 3 | NEGDWR(42);NWHDAAK(33);ITDSEENSWVVDMITK(46) | C-type lectin (CTL) or carbohydrate-recognition domain (CRD), CLECT domain, SM000034 | MFLSAVLVLCCISMVDSTCSTNPCDISTEKSMIASMVQCMLQSIENKIKASGECTAPVNCPAGWKKYKTNCYFFSPDGKNWHDAAKQCQTMGGYLAKITDSEENSWVVDMITKSVKHKHGYWMGMTDFKNEGDWRWVNDSSPVSYSNWRRGNPDNASNEDCGHFWSAANYEWNDAICSIDQMGYICECSDASNCRPSKG. |
| gi 223022517 | *M.galloprovincialis* | — | — | — | — | 126 | 4 | LKADSITADR(36);APNEVSDQPR(42);NAVQSMGEADGTGR(67);ELHANHINTENMR(56) | Low complexity region; Arg (10.8%), Lys (9.5%), Ala (9.1%) | MMKELFISIIAFQIISFIAEANAQKLDTLPKYYEEKLLRKIKHSLSPEEITIIEKFNNLKDFYPDGERHHVLRKRNAVQSMGEADGTGRRRRRRRRKQKPSAAIDGAINTSPDKAPNEVSDQPRRQQGRRRRPKPSDQTSIKSSNDRPTNPTPNEPRNAISDKPKRRQGKPRPSAVIQEAPVLSEANADSNDGLLKAKELHANHINTENMRANKLKADSITADRVKAQDVVV? |
| gi\|58307961 | *M.galloprovincialis* | gi\|725581350\| | acidic mammalian chitinase isoform X4 / Saimiri boliviensis boliviensis | 52.2 | 5.00E-47 | 125 | 2 | LVLGISTYGR(57);VPYAYSGDQWVGYDNV(94) | Glyco_18 domain (SM000636) | ?SQVGCLYLLDNGTPKEKLVLGISTYGRTFKLSSSSNNGFGAATVGGGSPGKNTRESGFLSYYEICSSGWTTVWNDEHKVPYAYSGDQWVGYDNVRSVTIKAQYIKEKGLGGAMFWALDLDDFTGNACREGDYPLISAVTNELKTSGFRPHPKPVTPKVPAERTTTTPRETTKSPITKQ? |
| gi\|58306383 | *M.galloprovincialis* | — | — | — | — | 124 | 2 | SALYEDTFIPEVIRPR(44);GVNDELVYTSNLMDDTYDVAAK(77) | No domains; Ser (15.8%), Arg (12.9%) | ?RSTSSTNEMTVRRSRYSSVPPGYFASTKGHSALKRWSYAPQSRSALYEDTFIPEVIRPRSYYDTSREENDIRRGVNDELVYTSNLMDDTYDVAAKSRSRNP? |
| gi\|164595782 | *M edulis* | gi\|301341836\| | arginine kinase / C. novaehollandiae | 78.88 | 1.00E-84 | 120 | 2 | LNLQPR(44);LGLTEYEAIQEMR(109) | ATP:guanido phosphotransferases (ATP-gua_Ptrans) domain;PF00217 | ?KKLTFAKKDGYGYLTFCPTNLGTTCRASVHIRIPKLSKLPEFKEFCEKLNLQPRGIHGEHTESVGGVFDISNKRRLGLTEYEAIQEMRKGVEEIIKKEKSL* |
| gi\|212830099 | *M.californianus* | — | — | — | — | 118 | 1 | YNPLSQNEVQYYLQELQELK(108) | Low complexity region; Leu (11.3%), Gly (9.0%), Glu (8.3%), Lys (8.3%) | MNKNIISLGLCMCSILLLTGADRQKRSIFSVKRYNPLSQNEVQYYLQELQELKERLNGGHGGKSKREKENLKKEVRNRHPGEKSRHGEQACPGGIAGMAKRPEVLQVHGHHHHHLINVTNQLHTEIQTVPPIL* |
| gi\|238643545 | *M.galloprovincialis* | gi\|353558673\| | Nacre serine protease inhibitor 2 / P. maxima | 31.58 | 2.00E-11 | 115 | 2 | IPMYYYSSQSGR(49); IPPYTGTSPLSHMIK(57) | BPTI/Kunitz family of serine protease inhibitors (KU) domain;SM000131 | MLRLLILLCMPVLYAQINFQQILAWKQLPRRCRIPPYTGTSPLSHMIKIPMYYYSSQSGRCESFNYSGLGKSKNMFRNPIDCLRKCACYAPMDAGTCTNSTTGTTRYYYNRRFKMCTTFQFSGCEGNDNNFSDYVSCHIACNRRRAEDL* |
| gi\|380851837 | *M edulis* | gi\|670984547\| | chitotriosidase-1 / U. maritimus | 53.64 | 2.00E-43 | 114 | 2 | LVLGISTYGR(57);TLNQDWAVQYWIDNGTPK(85) | Glyco_hydro_18 domain (PF00704) | MAYDFHGKDDRKTGHISPLYPRKEETGAERTLNQDWAVQYWIDNGTPKEKLVLGISTYGRTFKLSSSSNNGFGAATVGGGSAGKNTRESGFLSYYEICSLRMDNSME* |
| gi\|212821329 | *M.californianus* | gi\|405976801\| | Sodium-coupled monocarboxylate transporter 1 / C. gigas | 45.86 | 2.00E-34 | 110 | 2 | ELDATNLQTLN(67);IVGDELSTAHAVMK(70) | Low complexity region; Val (9.9%), Ser (9.4%) | MNIVYFTLILPFSMVSGIPTTPPNLKNNSLQSSLKELDATNLQTLNRETRKALLKIVGDELSTAHAVMKSIEPEVNDCQQKVDQKYYDCVQCVDMKCQNRYKECNGSIQISAPNSGGVSVSNSVNDRGRPTTTICTVMWGQSKACTTVMNPEGAFATSIKSMGDTIHVHLRSVVNDFGGSAGMFVNQMHSVVGDMNTSRVANEAVERMLNEL? |
| gi\|212812257 | *M.californianus* | gi\|405950795\| | Non-neuronal cytoplasmic intermediate filament protein / C. gigas | 59.27 | 2.00E-82 | 88 | 2 | IETLEDENAR(37);LNDQLGNNEGELANLR(80) | Filament domain (PF00038) | MSQEKVEIRRNIKTQPTIGTRSTVINRTSHGGGSIMPGGGSRSVSMRMSMGGSAPSFAQGTVSSMSHKNVANVLDTRAKEKTEMNVLNERFASYIEKVRFVEAQNKALLAEIDRLKKQKNFDASEIKELYEQEIADSRKIIDDLSDEKAKFDATLVSLQDQLEDERRDRINAEKTVDDLSNKIDRLNDQLGNNEGELANLRLRIETLEDENARLKKDKRTLQDDIGRIRADLDEETCKRIQAEMKLQT? |
| gi\|212814580 | *M.californianus* | gi\|322518389\| | MUSP-3; / M. californianus | 100 | 3.00E-89 | 83 | 2 | VDNGQGIAFR(65);TFRVDNGQGIAFR(53) | — | MLKGIILIVTIQLVNANFFGVFGKPLYNPFNKDKYMIDFITTFNKLMNMKQPQFPHPKSYPGFPPLFPGIKGKKSVFKTIDFTDMAPGSKKTFRVDNGQGIAFRSKSGNAGGMSFSSGTGGGKGFAFGGTLGGGSNGEFVMSQSGPGLKGGKVTYSKGVPKFAKGLFGMLPFFK* |
