# Supplementary material for: Layer-by-Layer Proteomic Analysis of Mytilus galloprovincialis Shell
Source: PLoS One. 2015 Jul 28;10(7):e0133913. doi: 10.1371/journal.pone.0133913 (PMC4517812; doi:10.1371/journal.pone.0133913)
Supplement: S4 Table — (DOCX) [file pone.0133913.s005.docx]

**S4 Table**

| **Matched EST** | **Organism** | **Homologue ID** | **Homologue name / Organism** | **Identity%** | **E-value** | **Protein score** | **Matched peptides** | **Sequence of matched peptides (score)** | **Domains (ID) or features** | **EST-derived seuqence (the signal peptides are underlined; "?" denotes undetermined amino acids, "*" denotes the stop codon)** |
| --- | --- | --- | --- | --- | --- | --- | --- | --- | --- | --- |
| gi\|212816630 | *M.californianus* | — | — | — | — | 779 | 2 | IIIQLLTR(56); SAAKIIIQLLTR(64) | Low complexity region;Gly(27.8%);Ala(20.6%);Ser(20.6%); Asp(13.5%) | ?GGAGGGSGAAASAAAAAAAGGRSGLIRWLVARRAAARAAASAGAGAGGIAVGAGGAGGAGGAGGAGAGGAGGAGGSGGSGGSGDGGGDGDCGSSDSDSGSDSDSDNDTDSSDSDTGSDASNSDSGSDSDGDGDSDSSGTSESSSSDSGDTSNEDSDDGSDDDDDDSFRSAAKIIIQLLTRLLMSGGFAGAGSSASASASAAASAGGGAGGAGLGLGGGSGAAS? |
| gi\|58308196 | *M.galloprovincialis* | gi\|14422379\| | calponin-like protein / M. galloprovincialis | 100 | 9.00E-117 | 549 | 8 | GMTGFGAVR(53); HISDIKVEDLDR(36); EGNTLLTLQAGTNR(79); HNYQGPTIGSKPTEK(52); NLPMVLATISHVGTEAQR(82); YGVPNTSLFQTVELYEAR(83); ADQFDKDGENIITLQAGTNK(94); VEDLDREGNTLLTLQAGTNR(40) | Calponin domain(PF00402) | ?KFLKAAHKYGVPNTSLFQTVELYEARNLPMVLATISHVGTEAQRHNYQGPTIGSKPTEKHRVQFSYEQLKQSHGTIGLQSGTNKFATQKGMRIGSVRHISDIKVEDLDREGNTLLTLQAGTNRFASQKGMTGFGAVRHIADIRADQFDKDGENIITLQAGTNKFASQ? |
| gi\|223026062 | *M.galloprovincialis* | gi\|14422379\| | calponin-like protein / M. galloprovincialis | 98.56 | 2.00E-146 | 411 | 9 | GMTGFGAVR(59); GMTSMGAVR(52); GMTSFGSQR(55); VSDLAEDMK(56); VSDLAEDMKR(74); EGNTLLTLQAGTNR(79); EAQSVIPLQYGTNR(68); ADEFDKDGENIITLQAGTNK(53); ADDFDPQTASHIGLQAGSNQFASQK(84) | Calponin domain(PF00402) | ?VRHISDIKVEDIDKEGNTLLTLQAGTNRFASQKGMTGFGAVRHIADIRADEFDKDGENIITLQAGTNKFASQKGMTGFGAVRHVSDIRADDFDPQTASHIGLQAGSNQFASQKGMTSMGAVRHICDIRADDLDREAQSVIPLQYGTNRGSSQKGMTSFGSQRHIADIKVSDLAEDMKRQDLNMTPKEYQEFRQQMEATEQKTDEPQYE* |
| gi\|223023515 | *M.galloprovincialis* | gi\|14422379\| | calponin-like protein / M. galloprovincialis | 93.79 | 2.00E-114 | 373 | 5 | YDSGLEYEVR(63); HDVTFSYEQLK(62); ALIGEDIGEGPSNVEK(108); NLPMVLATISHVGTEAQR(82); YGVPNTSLFQTVELYEAR(83) | Calponin domain(PF00402) | MADRVKPMGMDRALISKMGAKYDSGLEYEVRGWIKALIGEDIGEGPSNVEKSLRDGVILCTLMKKVIEGTPSESLPAACSKTDLKSSASELPFKQMENIEKFLKAAHKYGVPNTSLFQTVELYEARNLPMVLATISHVGTEAQRLNFNGETIGSKPTVKHDVTFSYEQLKQSCGLIG? |
| gi\|223026184 | *M.galloprovincialis* | gi\|14422379\| | calponin-like protein / M. galloprovincialis | 98.19 | 4.00E-157 | 352 | 6 | GMTGFGAVR(59); TDLKPSSSELPFK(40); HNYQGPTIGSKPTEK(52); NLPMVLATISHVGTEAQR(82); YGVPNTSLFQTVELYEAR(83); ADEFDKDGENIITLQAGTNK(53) | Calponin domain(PF00402) | MKKVIEGTPSESLPAACSKTDLKPSSSELPFKQMENIEKFLKAAHKYGVPNTSLFQTVELYEARNLPMVLATISHVGTEAQRHNYQGPTIGSKPTEKHIVQFSYEQLKQSHGTIGLQSGTNKFATQKGMRIGSVRHISDIKVEDLDKEGNTLLALQAGTNRFASQKGMTGFGAVRHIADIRADEFDKDGENIITLQAGTNKFAS? |
| gi\|223026117 | *M.galloprovincialis* | gi\|14422379\| | calponin-like protein / M. galloprovincialis | 99.35 | 2.00E-105 | 334 | 7 | GMTGFGAVR(59); GMTSFGSQR(55); VSDLAEDMK(56); VSDLAEDMKR(74); EAQSVIPLQYGTNR(68); GMTGFGAVRHIADIR(63); ADDFDPQTASHIGLQAGSNQFASQK(84) | Calponin domain(PF00402) | MTGFGAVRHVSDIRADDFDPQTASHIGLQAGSNQFASQKGMTSMGAVRHICDIRADDLDREAQSVIPLQYGTNRGSSQKGMTSFGSQRHIADIKVSDLAEDMKRQDLNMTPKEYQEFRQQMEATEQKTDEPQYE* |
| gi\|212823360 | *M.californianus* | gi\|14422379\| | calponin-like protein / M. galloprovincialis | 96.06 | 6.00E-140 | 315 | 4 | YDSGLEYEVR(63); HNYQGPTIGSKPTEK(52); NLPMVLATISHVGTEAQR(82); YGVPNTSLFQTVELYEAR(83) | Calponin domain(PF00402) | MADRVKPMGMDRALISKMGAKYDSGLEYEVRGWIKQLIGEDIGEGPSNVEKSLRDGVILCNLMKKVIDGTPSESLPAACAKTDLKSSPSELPFKQMENIEKFLKAAHKYGVPNTSLFQTVELYEARNLPMVLATISHVGTEAQRHNYQGPTIGSKPTEKHQVQFSYEQLKQSHGTIGLQSGTNKFATQKGMRIGSIRHISDIK? |
| gi\|58307533 | *M.galloprovincialis* | — | — | — | — | 298 | 8 | SSVTSNR(36); WSYAPQS(39); SRDQMLLR(37); SSSSTNEMTVR(61); DQMLLRDAAR(58); ALVDTETYVSPR(72); SALYEDTFIPEVIRPR(48); GVNDELVYTSNLMDDTYDVAAK(107) | No domains; Ser (14.2%), Arg (13.0%), Val (8.0%), Ala (8.0%) | MTVRRSRYSSVPPGYFASTKGHSALKRWSYAPQSRSALYEDTFIPEVIRPRSYYDTSREENDIRRGVNDELVYTSNLMDDTYDVAAKSRSRDQMLLRDAARALVDTETYVSPRSSVTSNRVRATSVVARPAPLTSRAVSCPPTSRRSNQPLYGGKSHWDEEG? |
| gi\|58306751 | *M.galloprovincialis* | gi\|524882698\| | collagen alpha-4(VI) chain-like / Aplysia californica | 39.85 | 4.00E-18 | 297 | 2 | LLMDGGLSTSHGSR(100); ELDFVGEVITAFDLGK(79) | von Willebrand factor type A (VWA) domain (SM000327) | MNAMLFLSLFSLLLIARVTARSPPAPPEPPVYRRCLKKIADVFFVVDTSSSLDITPNVIKELDFVGEVITAFDLGKDQVRTGMMTFATNTELLFKLDDFKTKKEIAEILYDRKNLVKYRWKGGNTNIGKALRLLMDGGLSTSHGSRADVPQIAVIITDGNSNDRADFDSALLELR? |
| gi\|145896743 | *M.californianus* | gi\|14422379\| | calponin-like protein / M. galloprovincialis | 82.02 | 1.00E-155 | 293 | 4 | GMTSMGAVR(52); YDSGLEYEVR(63); NLPMVLATISHVGTEAQR(82); YGVPNTSLFQTVELYEAR(83) | Calponin domain(PF00402) | MADRVKPMGMDRALISKMGAKYDSGLEYEVRGWIKQLIGEDIGEGPSNVEKSLRDGVILCNLMKKVIDGTPSESLPAACAKTDLKSSPSELPFKQMENIEKFLKAAHKYGVPNTSLFQTVELYEARNLPMVLATISHVGTEAQRLNFNGETIGSKPTVKHDVNFSYEQLKQSCGLIGLQSGTNKFASQKGMRIGAVRHIADIRAE* |
| gi\|37650124 | *M.galloprovincialis* | gi\|405963175\| | 60 kDa neurofilament protein / C. gigas | 87.97 | 2.00E-72 | 281 | 10 | VTLSANFQGAEESK(71); KPELPEGYLGPQEK(65); AVAVIAEGEWHHFEDR(92); FAELGLETLSHPEPEITAR(61) | Filament domain (PF00038) | MEFLKKVHEQELKELAALAYRDTTEENREFWKSELSQAIRDIQSEYDNKVDQLRGDMESYYNLKVQEFRTGATKQNMEVTHVKEENKKLVK? |
| gi\|223028013 | *M.galloprovincialis* | — | — | — | — | 251 | 4 | VTLSANFQGAEESK(71); VTLSANFQGAEESK(65); AVAVIAEGEWHHFEDR(92); FAELGLETLSHPEPEITAR(61) | No domains; Leu (10.3%), Lys (9.4%), Glu (8.1%) | MDKKPELPEGYLGPQEKFAELGLETLSHPEPEITARDNHLEIKIKAKTHVKFTTQLINCRNDKDLSKYVFAQTRESVVHFLVHMPESDYYKLQLYCLPAADPSKSLPNVYNYLIHCTRALQPVYPFPKQYAQWKDGCFIEEPRVLHTNSKLTNINWQVKVPNAKAVAVIAEGEWHHFEDRGHDMWTAKFDLDKYRGKNSKVTLSANFQGAEESKYSTLLEYLL* |
| gi\|58307802 | *M.galloprovincialis* | gi\|405967947\| | Transgelin-2 / C. gigas | 63.35 | 1.00E-64 | 243 | 8 | SPVNFQK(43); LINILLK(44); SGLGYEVEK(53); GIQDYGVDR(53); ESEFQSGDLWEVR(59); VMSPFVAMTNIENFNK(72); EEAAGTPTHVVNWVNAILGSEHDPIPGTDWK(58); MEANYDREEAAGTPTHVVNWVNAILGSEHDPIPGTDWK(42) | Calponin homology domain (SM000033) | MSGSGRANKSGLGYEVEKKMEANYDREEAAGTPTHVVNWVNAILGSEHDPIPGTDWKSICNHLRDGVALCKLINILLKKDGKSPVNFQKKVMSPFVAMTNIENFNKGIQDYGVDRESEFQSGDLWEVRKGPFLNVINCISSLGFVANKKGVTPKYTGEIRKYLDNE* |
| gi\|58307858 | *M.galloprovincialis* | gi\|390979787\| | distal byssal thread collagen / synthetic construct | 90.64 | 2.00E-24 | 238 | 3 | GPDGETGPQGPAGPK(68); GPDGETGPQGPAGPK(87); GPVGGQGPAGPAGPLGPQGPMGER(89) | Collagen domain (PF01391) | ?TRTQGPTGSEGPVGAPGPKGSVGDQGAQGDQGATGADGKPGDRGPDGETGPQGPAGPKGQVGDQGKPGAKGETGDQGARGEAGKAGEQGPGGIQGPKGPVGGQGPAGPAGPLGPQGPMGERGPQGPTGSEGPVGAPGPKGSVGDQGAQGDQGATGADGKKGEPGERGQQGAAGPTRTQGPTGSEGPVGAPGPKGSVGDQGAQGDQGATGADGKPGDRGPDGETGPQGPAGPKGQVGDQGKPGAKGETGDQGARGEAGKAGEQGPGGIQGPKGPVGGQGPAGPAGPLGPQGPMGERGPQGPTGSEGPVGAPGPKGSVGDQGAQGDQGATGADGKKGEPGERGQQGAAGP? |
| gi\|58308563 | *M.galloprovincialis* | — | — | — | — | 231 | 1 | AAAAAGASAAAGGSGGTLR(145) | Low complexity region; Ala (41.2%), Gly ((21.8%), Leu (8.8%) | ?FFFFFGYGYGGALDIDLGDLEELLGGLDTIDLEDAAVLSALGLGGGSGLGGGSAAAAAAAAAAAAGGLGGGSAAAAAAAAAAAAGGLGGGSAAAAAAAAAAAGGAGGIGGSSAAAAAAAAAAAGRRAAAAAGASAAGGSGGTLRQRLISRIIARRQSAASAAAAAAASAF? |
| gi\|58307171 | *M.galloprovincialis* | gi\|405967947\| | Transgelin-2 / C. gigas | 55.37 | 2.00E-62 | 226 | 7 | SPVNFQK(43);LINILLK(44);GIQDYGVDR(53);ESEFQSGDLWEVR(59);VMSPFVAMTNIENFNK(72);EEAAGTPTHVVNWVNAILGSEHDPIPGTDW(58);LEENYDREEAAGTPTHVVNWVNAILGSEHDPIPGTDWK(39) | Calponin domain(PF00402) | MSGRASKSGIGLKVEKKLEENYDREEAAGTPTHVVNWVNAILGSEHDPIPGTDWKSICNHLRDGVALCKLINILLKKDGKSPVNFQKKVMSPFVAMTNIENFNKGIQDYGVDRESEFQSGDLWEVRKGPFLNVINCISSLGFVANKKGVTPKYTGEIRKYLDNE* |
| gi\|223026852 | *M.galloprovincialis* | gi\|405967947\| | Transgelin-2 / C. gigas | 62.73 | 2.00E-62 | 224 | 7 | LINILLK(44); SGLGYEVEK(53); ESEFQSGDLWEVR(59); VMSPFVAMTNIENFNK(72); GIQDYGVDKESEFQSGDLWEVR(34); EEAAGTPTHVVNWVNAILGSEHDPIPGTDWK(58); MEANYDREEAAGTPTHVVNWVNAILGSEHDPIPGTDWK(42) | Calponin domain(PF00402) | MSGSGRANKSGLGYEVEKKMEANYDREEAAGTPTHVVNWVNAILGSEHDPIPGTDWKSICNHLRDGVALCKLINILLKKDGKSPINFQKKVMSPFVAMTNIENFNKGIQDYGVDKESEFQSGDLWEVRKGPFLNVINCISSLGFVANKKGATPKYTGEIRKYLDNE* |
| gi\|58306383 | *M.galloprovincialis* | — | — | — | — | 213 | 4 | WSYAPQSR（39);STSSTNEMTVR(53);SALYEDTFIPEVIRPR(48);GVNDELVYTSNLMDDTYDVAAK(107) | No domains; Ser (15.8%), Arg (12.9%) | ?RSTSSTNEMTVRRSRYSSVPPGYFASTKGHSALKRWSYAPQSRSALYEDTFIPEVIRPRSYYDTSREENDIRRGVNDELVYTSNLMDDTYDVAAKSRSRNP? |
| gi\|223026853 | *M.galloprovincialis* | gi\|405967947\| | Transgelin-2 / C. gigas | 54.8 | 1.00E-60 | 207 | 6 | LINILLK(44);ESEFQSGDLWEVR(59);VMSPFVAMTNIENFNK(72);GIQDYGVDKESEFQSGDLWEVR(34);EEAAGTPTHVVNWVNAILGSEHDPIPGTDWK(58);LEENYDREEAAGTPTHVVNWVNAILGSEHDPIPGTDWK(39) | Calponin domain(PF00402) | MSGRASKSGIGLKVEKKLEENYDREEAAGTPTHVVNWVNAILGSEHDPIPGTDWKSICNHLRDGVALCKLINILLKKDGKSPINFQKKVMSPFVAMTNIENFNKGIQDYGVDKESEFQSGDLWEVRKGPFLNVINCISSLGFVANKKGATPKYTGEIRKYLDNE. |
| gi\|164595782 | *M. edulis* | gi\|301341836\| | arginine kinase / C. novaehollandiae | 78.88 | 1.00E-84 | 198 | 4 | KGVEEIIK(43);KGVEEIIK(85);TFLVWVNEEDHLR(50);GIHGEHTESVGGVFDISNK(105) | ATP:guanido phosphotransferases (ATP-gua_Ptrans) domain;PF00217 | ?KKLTFAKKDGYGYLTFCPTNLGTTCRASVHIRIPKLSKLPEFKEFCEKLNLQPRGIHGEHTESVGGVFDISNKRRLGLTEYEAIQEMRKGVEEIIKKEKSL* |
| gi\|223021963 | *M.galloprovincialis* | gi\|405967947\| | Transgelin-2 / C. gigas | 59.76 | 7.00E-62 | 196 | 5 | LINILLK(44); GIQDYGVDR(53); GIQDYGVDR(59); VMSPFVAMTNIENFNK(72); EEAAGTPTHVVNWVNAILGSEHDPIPGTDWK(58) | Calponin domain(PF00402) | MSFRASKSGLGYEVQRKLELNYDREEAAGTPTHVVNWVNAILGSEHDPIPGTDWKSICNHLRDGVALCKLINILLKKDGKSPINFQKKVMSPFVAMTNIENFNKGIQDYGVDRESEFQSGDLWEVRKGPFLNVINCIPSLGFVANKKGATPKYTGEIRKYLDNE* |
| gi\|145896099 | *M.californianus* | gi\|405962873\| | Filamin-C / C. gigas | 79.38 | 3.00E-167 | 196 | 3 | VTEPGEYLVSIK(49); EAGAGGLSIAVEGPSK(76); VVAPSGTEEEAIIQEIDDGQYAVR(96) | Filamin-type immunoglobulin domains (SM000557) | MHIGSQCELSLKIPGTSPFDMTASVTNPSGITELCDIVSLDDNHYSIKFVPKEMGVHTVSVKHKDMHIPGSPFEFTVGPIAGGGSHKVHAAGPGLERGEVDQPCDFNIYTREAGAGGLSIAVEGPSKAELDFDDRKDGSCGVTYRVTEPGEYLVSIKFNDERIPDSPFKVDICPSIGDARKMSVSALQQKGLQVGKPAAFVVNFNDAQKGKLKAKVVAPSGTEEEAIIQEIDDGQYAVRFIPRENGGHNVHVFFNDCEIPESPFRIMVXKVDCDPGMVHASXDGLXTGHTGSPAKFLGHTVNAGTGALGVTVGGSSKGQIRMWLKRMRDIDFHTPQQLLKTTKYY? |
| gi\|223025178 | *M.galloprovincialis* | — | — | — | — | 193 | 2 | MMMMMPEMGGK(43); GMGMPMMMPEMGGK(55) | Met (44.1%), Gly (27.6%) | MMMMPEMGGKGMGMMEKGMGMPMMMPEMGGKGMGMMEKMMMMMPEMGGKGMGMMEKGMGMPMMMPETGGKGMGMMEGGMGGKDMGMMMMPKMEMMGGKGMGMMEMPMSMPMMKGGMPMPMMEGGMGG |
| gi\|223026112 | *M.galloprovincialis* | gi\|14422379\| | calponin-like protein / M. galloprovincialis | 100 | 1.00E-50 | 183 | 4 | GMTSFGSQR(55); VSDLAEDMK(56); VSDLAEDMKR(74); EAQSVIPLQYGTNR(68) | Calponin domain(PF00402) | ICDIRADDLDREAQSVIPLQYGTNRGSSQKGMTSFGSQRHIADIKVSDLAEDMKRQDLNMTPKEYQEFRQQMEATEQKTDEPQYE. |
| gi\|223026186 | *M.galloprovincialis* | gi\|14422379\| | calponin-like protein / M. galloprovincialis | 96.47 | 4.00E-48 | 183 | 4 | GMTSFGSQR(55); VSDLAEDMK(56); VSDLAEDMKR(74); EAQSVIPLQYGTNR(68) | Calponin domain(PF00402) | ICDIRADDLDREAQSVIPLQYGTNRGSSQKGMTSFGSQRHIADIKVSDLAEDMKRQDLNMTPKEYQAFRQQMEATEKKTDEPEYE. |
| gi\|58307336 | *M.galloprovincialis* | gi\|21105303\| | precollagen-D / M. galloprovincialis | 98.95 | 6.00E-20 | 180 | 2 | GPDGETGPQGPAGPK(68);GPVGGQGPAGPAGPLGPQGPMGER(89) | Collagen domain (PF01391) | SPDPRTTGPDGAMGPQGPCGDRGAPGVPGKQGPVGGQGPAGPRGPRGDEGPVGPKGEPGAKGADGKPGDRGPDGETGPQGPAGPKGQVGDQGKPGAKGETGDQGARGEAGKAGEQGPGGIQGPKGPVGGQGPAGPAGPLGPQGPMGERGPQGRTPAGTPGPPGNPGEPGQGGAPGAPG |
| gi\|145887968 | *M.californianus* | gi\|322966920\| | Shell matrix protein / M. californianus | 100 | 2.00E-171 | 177 | 5 | TGGNLEIR(60); TGGNLEIR(43); LYIWGFQSR(48); INFDDGFKDISK(64); GGLAFDYSHISLR(83) | Laminin_G_3 domain (PF13385) | MNGNGGLRGSARKQFRQCSAEFKINFDDGFKDISKGGLAFDYSHISLRRGKGVFVGNSKLYIWGFQSRFLGKTFAIRMKVKIKRGAGKYRPEPIISNCGPNGDSSVEIVVHRGKVIFKAKTSDNPEAVFITEDYDDDKWTDLTYYYDGNHFGGSCNGRPFRQRTGGNLEIRDNPMTIGLCTGQNGFHGEIDELEIYTACIPKDM* |
| gi\|145887813 | *M.californianus* | gi\|322966920\| | Shell matrix protein / M. californianus | 100 | 4.00E-150 | 174 | 5 | INFDDGFK(43); LYIWGFQSR(48); YGYLAPQYGGLR(49); INFDDGFKDISK(64); GGLAFDYSHISLR(83) | No domains | MTTIKNVSEHQAGICVTMSVKLPAILTRQLAQTSCPSLPDPMNRYGYLAPQYGGLRIRACPSGTIYSENQCRYKSNMNGNGGLRGSARKQFRQCSAEFKINFDDGFKDISKGGLAFDYSHISLRRGKGVFVGNSKLYIWGFQSRFLGKTFAIRMKVKIKRGAGKYRPEPIISNCGPNGDSSVEIVVHRGKVIFKAKH? |
| gi\|58305806 | *M.galloprovincialis* | gi\|405975735\| | Collagen alpha-5(VI) chain / C. gigas | 40.45 | 7.00E-14 | 162 | 2 | IAIIITDGKPTDINATQR(41); HAFLIEDFDSLSSFEAK(88) | von Willebrand factor type A (VWA) domain (SM000327) | SVGGIQRFLKIAIIITDGKPTDINATQRRVKEAKQQGIIMFAIGVGEWRNKDEINLLASDPVDKHAFLIEDFDSLSSFEAKFAKKTCTAAIQAISMPPEGFY* |
| gi\|58307379 | *M.galloprovincialis* | gi\|524892586\| | Collagen alpha-5(VI) chain / C. gigas | 31.97 | 3.00E-15 | 162 | 2 | IAIIITDGKPTDINATQR(41); HAFLIEDFDSLSSFEAK(88) | von Willebrand factor type A (VWA) domain (SM000327) | MKAISKVQYLDTNENMQFTYTGKALQMLIQKGFLKQNGGRGGKVPKIAIIITDGKPTDINATQRRVKEAKQQGIIMFAIGVGEWRNKDEINLLASDPVDKHAFLIEDFDSLSSFEAKFAKKTCTAAIQAISMPPEGF? |
| gi\|223022743 | *M.galloprovincialis* | gi\|524885601\| | arginine kinase-like isoform X1 / Aplysia californica | 67.95 | 4.00E-108 | 156 | 3 | TFLVWVNEEDHLR(50); TFLVWVNEEDHLR(57); LDFGDIDPSGEMIVSTR(73) | ATP:guanido phosphotransferases (ATP-gua_Ptrans) domain;PF00217 | MSDLAELWKKLSGAKYPDECKSLLKQCLTQELFDQLKDKKTLLNGTLADCIRSGAKNLDSGVGLYVCDPEAYTTFKPLFDAVIKMYHKVDTINHPKPDFGDISKLDFGDIDPSGEMIVSTRVRVGRSHDGYSFPPCSNKEARIDMLKKTEEACATLPGELAGKMYRLEGMSKTDEQQLIDDHFLFKNDDRMLGDAGGYADWPIGRGIFHNPKKTFLVWVNEEDHLRFISMQMGGNL? |
| gi\|238643251 | *M.galloprovincialis* | gi\|524892586\| | Collagen alpha-5(VI) chain / C. gigas | 30 | 4.00E-26 | 156 | 2 | QIERSLK(37);ELNFVGDVIDAFDVGSDQVR(99) | von Willebrand factor type A (VWA) domain (SM000327) | MMTFSNDPEMLFQLDDFKTKEEIAKVLMEMNANDWKGGNTFMDKALRLLMKEGLSTSHGSRYGVPQIAVIITDGRATDRKEFEKAVNELRQTNYLVFAIGVGPKRDPVELKKIASDSSRVYEVENVQSLQAIRQELVVKLCEQGDQPAPPVVTCEGAQADVIFVADSSRSIGSAAFNELKKFAVDVVKRFTVSPSDIQVGLIIFGNDTNFEFTLGSYR? |
| gi\|223026116 | *M.galloprovincialis* | gi\|14422379\| | calponin-like protein / M. galloprovincialis | 98.63 | 2.00E-97 | 149 | 4 | GMTGFGAVR(59);HISDIKVEDLDR(36);EGNTLLTLQAGTNR(79);VEDLDREGNTLLTLQAGTNR(40) | Calponin domain(PF00402) | ?RVQFSYEQLKQSHGTIGLQSGTNKFATQKGMRIGSVRHISDIKVEDLDREGNTLLTLQAGTNRFASQKGMTGFGAVRHIADIRADQFDKEGENIITLQAGTNKFASQKGMTGFGAVRHVSDIRADDFDPQTSSHIGLQAGSNQFAS? |
| gi\|145893257 | *M.californianus* | — | — | — | — | 149 | 2 | AQSLIDEAEQR(62);KAQSLIDEAEQR(85) | Arg (10.6%), Lys (9.6%), Ser (9.6%) | LKKRRQVPSSRIPVIPMNPDLQGMLKRRRRSNITWNNIRKAQSLIDEAEQRANMGKRTCKPYADHDPCCFTGGNQSSQGLNSYFKKKDFIESSF |
| gi\|223021924 | *M.galloprovincialis* | — | — | — | — | 143 | 6 | MMMMMPEMGGK(43);GMGMPMMMPEMGGK(36); | Low complexity region; Met (42.3%), Gly (28.2%),Lys (10.6%) | ?WKKMMMMMPEMGGKGMGMMMPEMGGKGMGMMMPEMGGKGMGMMMPEMGGKGMGVMMPEMGGKGMGMMMPEMGGKGMGMMMPEMGGKGMGMMEKGMGMPMMMPEMGGKGMGMMEKMMMMMPEMGGKGMGMMKKGMGMPIMMPE? |
| gi\|58306781 | *M.galloprovincialis* | gi\|405975242\| | Calponin-2 / C. gigas | 60.28 | 2.00E-45 | 133 | 4 | GMTSMGAVR(52); EINLQSGTNK(37); TTDLAEEWAEK(82); EGSAVLRPDMGYSGGDSQK(60) | Calponin domain(PF00402) | MTSMGAVRHICDIRADQYDPESNKEINLQSGTNKFDSQCGMRGFGAIRHISDVKVNELDREGSAVLRPDMGYSGGDSQKGMTCFGAQRHVTNIKTTDLAEEWAEKYGKPTTPRVQQQEEIPEEDNE* |
| gi\|223020794 | *M.galloprovincialis* | gi\|405962873\| | Filamin-C / C. gigas | 86.15 | 1.00E-95 | 125 | 2 | TIADLENDFSDGLR(50); IVNIDSSDIVDSNLK(99) | Calponin homology domain (SM000033) | MAESEAQYYDEDEDEEMPLTERDLADDAQWKLIQKNTFTRWANEHLKTVNKTIADLENDFSDGLRLVALIEVLSGKKFKHVNRRPNFRTQKLENVTRVLEFLERDEGIRIVNIDSSDIVDSNLKLILGLIWTLILHYSISMPMWEGEEPGPQEGGPTPKQRLLNWVQSKVPDIPIKNFNNDWNNGKAIGALVDAV? |
| gi\|212830099 | *M.californianus* | — | — | — | — | 125 | 1 | YNPLSQNEVQYYLQELQELK(121) |  | MNKNIISLGLCMCSILLLTGADRQKRSIFSVKRYNPLSQNEVQYYLQELQELKERLNGGHGGKSKREKENLKKEVRNRHPGEKSRHGEQACPGGIAGMAKRPEVLQVHGHHHHHLINVTNQLHTEIQTVPPIL* |
| gi\|237638644 | *M. coruscus* | gi\|405958789\| | Arginine kinase / C. gigas | 74.16 | 5.00E-106 | 124 | 3 | KGVEEIIK(43);LGLTEYEAIQEMR(85);TFLVWVNEEDHLR(50) | ATP:guanido phosphotransferases (ATP-gua_Ptrans) domain;PF00217 | MLKKTEEACSQLPGELAGKMYHLQGMSKADEQQLIDDHFLFKNDDRFLGDAGGYADWPVGRGIFHNPKKTFLVWVNEEDHLRFISMQMGGNLGEVYKRLVNGIQELEKKLTFAKKDGYGYLTFCPTNLGTTCRASVHIKIPKLSKLPEFKDFCEKLNLQPRGIHGEHTESVGGVYDISNKRRLGLTEYEAIQEMRKGVEEIIKKEKSL* |
| gi\|238644079 | *M.californianus* | gi\|405975785\| | Filamin-A / C. gigas | 65.25 | 3.00E-100 | 120 | 2 | KGTLGDININIDGPTK(64);KGTLGDININIDGPTK(74) | Filamin domain (PF00630);Filamin-type immunoglobulin domains (SM000557) | MPTAQGLAPEIKDCDDGSIIVQYKPSKSGTHEVQMSYEGSATEGSPFSCVVDEIGGAYVTAFGAGLVGGMSGQTQSFTITAKKGTLGDININIDGPTKTDYKRQDSGDRCDVKFMPMTPGAYNIDIKYKGKVIKGSPFVSKVSGEGRKRSQISLGNASEYALNVMEPDIVDLVGSIKGPKGNFEPCILKKSKDGHLCISSFSPKVAGDYKVQVYRDEKNIKGSPFPVTV? |
| gi\|223028643 | *M.galloprovincialis* | gi\|405964948\| | Fructose-bisphosphate aldolase / C. gigas | 90.95 | 9.00E-125 | 115 | 3 | LLQDQGIIPGIK(46);GILAADESTGSIGK(74);FAPINVENTEENR(54) | Glycolytic domain (PF00274) | VFPQNLSPEQEDELRKIANAIVAPGKGILAADESTGSIGKRFAPINVENTEENRRRYRELLFTCDKSLAQNISGVIMFHETFYQKNKAGVPFPKLLQDQGIIPGIKVDKGVVPLAGTDNECTTQGLDGLSERCAQYKKDGAQFAKWRCVLKIQQYTPSYQAMLENANVLARYASICQQNGLVPIVEPEVLPDGEHDLATAQKVTEQVLAF? |
| gi\|212817532 | *M.galloprovincialis* | gi\|405964948\| | Fructose-bisphosphate aldolase / C. gigas | 86.85 | 9.00E-144 | 115 | 3 | LLQDQGIIPGIK(46); GILAADESTGSIGK(74); GILAADESTGSIGK(54) | Glycolytic domain (PF00274) | MPSFPQNLSPEQEDELRKIANAIVAPGKGILAADESTGSIGKRFAPINVENTEENRRRYRELLFTCDKSLAQNISGVIMFHETFYQKTKAGVAFPKLLQDQGIIPGIKVDKGVVPLAGTDNECTTQGLDGLSERCAQYKKDGAQFAKWRCVLKIQQYTPSYQAMLENANVLARYASICQQNGLVPIVEPEVLPDGEHDLATAQKVTEQVLAFTYKALADHHVFLEGTLLKPNMVT? |
| gi\|212814586 | *M.californianus* | gi\|405958789\| | Arginine kinase / C. gigas | 78.38 | 9.00E-52 | 115 | 2 | KGVEEIIK(43); GIHGEHTESVGGVFDISNK(105) | — | ?RLVNGIQELEKKLTFAKKDGYGFLTFCPTNLGTTCRACVHIKIPNLSKLPEFKDFCEKLNLQPRGIHGEHTESVGGVFDISNKRCLGLCEYEAIQEMRKGVEEIIKKEKSL* |
| gi\|58307710 | *M.galloprovincialis* | gi\|21105303\| | precollagen-D / M. galloprovincialis | 91.3 | 7.00E-15 | 110 | 2 | GEQGAPGVITLVIEDLR(38); GSVGDQGAQGDQGATGADGK(87) | Collagen domain (PF01391) | ?FAPGPKGSVGDQGAQGDQGATGADGKKGEPGERGQQGAAGPVGRPGPRGDRGAKGIQGSRGRPGGMGRRGNRGSQGAVGPRGETGPDGNQGQRGEQGAPGVITLVIEDLRTAGVESPD* |
| gi\|223022206 | *M.galloprovincialis* | — | — | — | — | 109 | 2 | MMMMMPEMGGK(43); GMGMPMMMPEMGGK(36) | Met (44.1%), Gly (26.6%) | ?GMPMMMPEMGGKGMGMMEKMMMMMPEMGGKGMGMIEKGMGMPMMMPEMGGKGMGMM? |
| gi\|239585859 | *M. coruscus* | gi\|405975242\| | Calponin-2 / C. gigas | 53.19 | 1.00E-68 | 109 | 4 | GMTSMGAVR(52); EINLQSGTNK(37); ESEGQISLQSGTNK(72); EGSAVLRPDMGYAGGDSQK(42) | Calponin domain(PF00402) | ?CQFSYEQLKQSCGLIGLQSGTNKFASQKGMRIGAVRHIADIRADKFDKESEGQISLQSGTNKFASQKGMTSMGAVRHICDIRADEYDPESNREINLQSGTNKFDSQAGMRGFGAIRHIADVKVNELDREGSAVLRPDMGYAGGDSQKGMTCFGAQRHVTNIKTTDLAEEYAEKYGKPATQRLAQTEEVPEDNE* |
| gi\|145889304 | *M.californianus* | — | — | — | — | 106 | 2 | VTLSANFQGAEESK(71); KPELPEGYLGPQEK(65); AVAVIAEGEWHHFEDR(92); FAELGLETLSHPEPEITAR(61) | low complexity; Leu (10.3%), Lys (9.0%), Glu (8.5%) | MDKKPELPEGYLGPQEKFSELGLETLSHPEPEITARDNHLEIKIKAKTHVKFTTQLINCRNDEDLSKFVFAQTRENVVHFLVHVPESDYYKLQLYCLPAADPSKSLPNVYNYLINCTRALQPVYPFPKQYAQWKDGCFIEEPRVLHTNSKLTNINWQVKVPYAKAVAVVSEGEWHHFEHRGHGMCTAKFDLHKYRGKNTKVTLSANFQGAEESKYSTLLEYLL* |
| gi\|223025603 | *M.galloprovincialis* | gi\|403310251\| | Nacrein-like 3 protein / P. vulgata | 42.37 | 2.00E-18 | 105 | 3 | INDFAIGITVK(49); NIYETVIAFGVR(47); GVTYDVGLEGFPAFK(68) | Thrombospondin N-terminal -like domain, TSPN domain (SM000210) | MIKMWHFQVIFPLVCIFVQVYTQSETEVDLLAAIGLPDPSKGVTYDVGLEGFPAFKLDKSSYIRKAAETYFTDRIGKINDFAIGITVKVFSKDGILFAVKNIYETVIAFGVRITATGNGKHNVILYYKEDHQYGQMSVTIGNF? |
| gi\|212815663 | *M.californianus* | gi\|524885601\| | arginine kinase-like isoform X1 / A. californica | 69.51 | 1.00E-115 | 103 | 2 | TFLVWVNEEDHLR(50); TDEQQLIDDHFLFK(57) | ATP:guanido phosphotransferases (ATP-gua_Ptrans) domain;PF00217 | MSDLAELWKRLNGAKYPDECKSLLKQCLTQEMFDQLKNKKTSLNGTLADCIRSGAKNLDSGVGLYACDPEAYTTFKPLFDAVIKMYHKVDTINHPKPEFGDISKLDFGDLDPSGDMIVSTRVRVGRSHDGYSFPPCSNKEARVDMLKKTEEACAQLPGELAGKMYKLEGMSKTDEQQLIDDHFLFKNDDRFLADAGGYADWPIGRGIFHNPKKTFLVWVNEEDHLRFISMQMGGNLGEVYKRLVN? |
| gi\|145898022 | *M.californianus* | gi\|405967947\| | Transgelin-2 / C. gigas | 59.29 | 3.00E-50 | 100 | 3 | SPITFQK(34); SGLGYEVEK(53);RNLIIFSR(40) | Calponin homology domain (SM000033) | ?SEFLDSRCLEAVVQINPAWDMKWKRRWKRTTTVEEAAGTPTHVFNWVNAILGSEHDHIPGTDWKSICNHLRDGVALCKLVNILLKKDGKSPITFQKKVMSPFVAMTNIENFNKGCEQYGLEKEFQFQSGDLWEVRKGPFLNVINCLHNLGFCSNSKKVIPAYTGQIVKYLDNE* |
| gi\|238645227 | *M.galloprovincialis* | gi\|325504479\| | putative C1q domain containing protein MgC1q90 / M. galloprovincialis | 100 | 9.00E-132 | 98 | 2 | DGFIDLNDLR(52); EMYSSLGAVPQETVMK(68) | Complement component C1q domain (SM000110) | ?ISQCSCHCSVKNSVVPTQKTQSRMPSQCSTMQVICQTSPPTLTDVRKDLLDLDSYVDNKLRAIDTKIQQTACYNQGHTIVTFLARLVTPTHGTIAAKATLKFEKTTENVGNGYGNKTGIFTAPVKGLYHFTASARQSRSGYLHLGLYRNDEEMAVSVGVNYNSLTIGATFTLQYGDHVLVKNIWTQSSGIVGAGQSYFSGHLVHVM* |
| gi\|58307208 | *M.galloprovincialis* | — | — | — | — | 92 | 1 | SGFESGQGSGAVIENVR(92) | Internal repeats, Ser (14.3%), Gly (9.1%), Cys (9.1%) | MDYIWIKLIAVCVCSLVVSVDLSAAAVCKGCWVLGQFKEGNTEFEYKNGNCLEMTGCYCSCDGKFFCRKERNICDRTVESGRDFYSSSRRTSYQSSSSSRSGFESGQGSGAVIENVRNSACDRKCIVDGAEIDGGTYFSHKNQCISYSSCYXWL* |
| gi\|145898955 | *M.californianus* | gi\|405972863\| | Adenylate kinase isoenzyme 1 / C. gigas | 71.35 | 3.00E-92 | 92 | 2 | VLFVIGGPGSGK(61); GELVPLDEVLALLR(36) | ATPases Associated with diverse cellular Activities (AAA)domain;PF13671 | MGCIASKQKEKSTGGLKDAKVLFVIGGPGSGKGTQCAKIVEKYGFCHLSSGDLLRAEVQSESDRGKRLNEIMEKGELVPLDEVLALLRDAMEKKVSEGVKCFLIDGYPRELEQGERFEKEVAGCTGVLYFEVSDDTMTQRLLERGKTSGRVDDNEETIKKRLETFHNQTKPLIDHYKDRSNIIKAEGTVDEIFTEVQKYMDSKKW* |
