# Supplementary material for: Layer-by-Layer Proteomic Analysis of Mytilus galloprovincialis Shell
Source: PLoS One. 2015 Jul 28;10(7):e0133913. doi: 10.1371/journal.pone.0133913 (PMC4517812; doi:10.1371/journal.pone.0133913)
Supplement: S5 Table — (DOCX) [file pone.0133913.s006.docx]

**S5 Table**

| **Matched EST** | **Organism** | **Homologue ID** | **Homologue name / organism** | **Identity%** | **E-value** | **Protein score** | **Matched peptides** | **Sequence of matched peptides (score)** | **Domains or features** | **EST-derived seuqence (the signal peptides are underlined; "?" denotes undetermined amino acids, "*" denotes the stop codon)** |
| --- | --- | --- | --- | --- | --- | --- | --- | --- | --- | --- |
| gi\|212816630 | *M.californianus* | — | — | — | — | 1230 | 2 | IIIQLLTR(63); SAAKIIIQLLTR(57) | Low complexity region;Gly(27.8%);Ala(20.6%);Ser(20.6%); Asp(13.5%) | ?GGAGGGSGAAASAAAAAAAGGRSGLIRWLVARRAAARAAASAGAGAGGIAVGAGGAGGAGGAGGAGAGGAGGAGGSGGSGGSGDGGGDGDCGSSDSDSGSDSDSDNDTDSSDSDTGSDASNSDSGSDSDGDGDSDSSGTSESSSSDSGDTSNEDSDDGSDDDDDDSFRSAAKIIIQLLTRLLMSGGFAGAGSSASASASAAASAGGGAGGAGLGLGGGSGAAS? |
| gi\|58308563 | *M.galloprovincialis* | — | — | — | — | 546 | 4 | LISRIIAR(35); AAAAAGASAAAGGSGGTLR(146); RAAAAAGASAAAGGSGGTLR(104); AAAAAGASAAAGGSGGTLRQR(43) | Low complexity region; Ala (41.2%), Gly ((21.8%), Leu (8.8%) | ?FFFFFGYGYGGALDIDLGDLEELLGGLDTIDLEDAAVLSALGLGGGSGLGGGSAAAAAAAAAAAAGGLGGGSAAAAAAAAAAAAGGLGGGSAAAAAAAAAAAGGAGGIGGSSAAAAAAAAAAAGRRAAAAAGASAAGGSGGTLRQRLISRIIARRQSAASAAAAAAASAF? |
| gi\|212820708 | *M.californianus* | gi\|146152115\| | chitin synthase / M. galloprovincialis | 93.51 | 2.00E-68 | 413 | 1 | LLLNIFSR(54) | Low complexity region | MPQWGPFLHLMSSTRIVWLKTVQTEEDFVRFVVNEAQRLQRMEPAPDYDDLPPDYEDDDTTTTTYNMPDEQYDELPFLPPTPDGTIKSEPKARKRHSGPSDPNIPLLQQIFEDRLENIHRKWKQGTLAFRNSDKRRFNRFDDNRFSQKDDIMREKMFKRSFKKNSTDENIHTAIKIDTI* |
| gi\|58307858 | *M.galloprovincialis* | gi\|390979787\| | distal byssal thread collagen / synthetic construct | 90.64 | 2.00E-24 | 361 | 4 | AGEQGPGGIQGPK(45); GPDGETGPQGPAGPK(60); GSVGDQGAQGDQGATGADGK(103); GPVGGQGPAGPAGPLGPQGPMGER(111) | Internal repeats | ?TRTQGPTGSEGPVGAPGPKGSVGDQGAQGDQGATGADGKPGDRGPDGETGPQGPAGPKGQVGDQGKPGAKGETGDQGARGEAGKAGEQGPGGIQGPKGPVGGQGPAGPAGPLGPQGPMGERGPQGPTGSEGPVGAPGPKGSVGDQGAQGDQGATGADGKKGEPGERGQQGAAGP? |
| gi\|223022517 | *M.galloprovincialis* | — | — | — | — | 331 | 6 | ADSITADR(64); LKADSITADR(49); APNEVSDQPR(81); NAVQSMGEADGTGR(93); ELHANHINTENMR(61); SSNDRPTNPTPNEPR(37) | Low complexity region;Arg (10.8%), Lys (9.5%), Ala (9.1%) | MMKELFISIIAFQIISFIAEANAQKLDTLPKYYEEKLLRKIKHSLSPEEITIIEKFNNLKDFYPDGERHHVLRKRNAVQSMGEADGTGRRRRRRRRKQKPSAAIDGAINTSPDKAPNEVSDQPRRQQGRRRRPKPSDQTSIKSSNDRPTNPTPNEPRNAISDKPKRRQGKPRPSAVIQEAPVLSEANADSNDGLLKAKELHANHINTENMRANKLKADSITADRVKAQDVVV? |
| gi\|58307336 | *M.galloprovincialis* | gi\|21105303\| | precollagen-D / M. galloprovincialis | 98.95 | 6.00E-20 | 298 | 3 | AGEQGPGGIQGPK(45); GPDGETGPQGPAGPK(60); GPVGGQGPAGPAGPLGPQGPMGER(111) | Collagen domain (PF01391) | ?SPDPRTTGPDGAMGPQGPCGDRGAPGVPGKQGPVGGQGPAGPRGPRGDEGPVGPKGEPGAKGADGKPGDRGPDGETGPQGPAGPKGQVGDQGKPGAKGETGDQGARGEAGKAGEQGPGGIQGPKGPVGGQGPAGPAGPLGPQGPMGERGPQGRTPAGTPGPPGNPGEPGQGGAPGAPG? |
| gi\|58306883 | *M.galloprovincialis* | gi\|322966877\| | Perlucin-like protein/ M. galloprovincialis | 100 | 7.00E-112 | 245 | 3 | NEGDWR(48); NWHDAAK(35); ITDSEENSWVVDMITK(96) | C-type lectin (CTL) or carbohydrate-recognition domain (CRD)CLECT domain;SM000034 | MGKLTVVGILTLFIFYIVAASGKCTAPVNCPAGWKKYKTNCYFFSPDGKNWHDAAKQCQTMGGYLVKITDSEENSWVVDMITKSVKHKYGYWMGMADLKNEGDWRWVNDSSAVSYSNWHRGQPNNANNEDCGHFWSAVNYEWNDIVCNTDQMGYIC* |
| gi\|238644365 | *M.galloprovincialis* | gi\|322966877\| | Perlucin-like protein/ M. galloprovincialis | 81.58 | 3.00E-88 | 245 | 3 | NEGDWR(48); NWHDAAK(45); ITDSEENSWVVDMITK(96) | C-type lectin (CTL) or carbohydrate-recognition domain (CRD)CLECT domain;SM000034 | MFLSAVLVLCCISMVDSTCSTNPCDISTEKSMIASMVQCMLQSIENKIKASGECTAPVNCPAGWKKYKTNCYFFSPDGKNWHDAAKQCQTMGGYLAKITDSEENSWVVDMITKSVKHKHGYWMGMTDFKNEGDWRWVNDSSPVSYSNWRRGNPDNASNEDCGHFWSAANYEWNDAICSIDQMGYICECSDASNCRPSKG* |
| gi\|223023515 | *M.galloprovincialis* | gi\|14422379\| | calponin-like protein / M. galloprovincialis | 93.79 | 2.00E-114 | 241 | 4 | HDVTFSYEQLK(41); ALIGEDIGEGPSNVEK(70); NLPMVLATISHVGTEAQR(62); YGVPNTSLFQTVELYEAR(96) | Calponin domain(PF00402) | MADRVKPMGMDRALISKMGAKYDSGLEYEVRGWIKALIGEDIGEGPSNVEKSLRDGVILCTLMKKVIEGTPSESLPAACSKTDLKSSASELPFKQMENIEKFLKAAHKYGVPNTSLFQTVELYEARNLPMVLATISHVGTEAQRLNFNGETIGSKPTVKHDVTFSYEQLKQSCGLIG? |
| gi\|212830512 | *M.californianus* | gi\|322518586\| | Fibronectin-like protein / M. californianus | 100 | 6.00E-137 | 240 | 3 | TPDIIPSR(37); LADMEQATQELVR(82); SIVQNGQTLLDFIYQER(94) | Fibronectin type 3 domain (SM000060) | MFSFGIILLTVVSFTNAQWRQDMFTTAENQLRSIVQNGQTLLDFIYQERQKHGGGNMTGGSLMSHNVAYSSFINDVETRLADMEQATQELVRIMRTCPDAPLAPPPPTNVIVESTTIDNVSSIVVKWDPPFNPPENMQYKVYFVPVDQNGMQTAGEVVFRICDSTQTIASITDLTPRSRYRIRVGAVAGAVAEGASMPLNVKTPDIIPSRVRNVMVK? |
| gi\|212830390 | *M.californianus* | gi\|391359391\| | Valine-rich protein;Alveoline-like protein;/ P. margaritifera | 35.78 | 2.00E-06 | 217 | 2 | IVHIPIPK(34); VNQVVDLIHEVPMVVVK（76) | Actin domain (PF00022) | MESAGIHETTYNSIMKCDVDIRKDLYANTVLSGGTTMFPGIADRMQKEITALAPSTMKIXIIAPPERKYSVWIGGSILASL? |
| gi\|212812802 | *M.californianus* | gi\|391359391\| | Valine-rich protein (Alveoline-like protein) / P. margaritifera | 35.78 | 2.00E-06 | 217 | 2 | IVHIPIPK(34); VNQVVDLIHEVPMVVVK(76) | Val (19.7%), Pro (12.7%), Lys (8.6%) | MLPVVLFISLFAIGTLGHAPPPLQKKIVHIPIPKAVVRHKPLRQDRAHDVHQHVPLKRTEVRRVNQVVDLIHEVPMVVVKHKKFLSPVIIPKPIVVNEVLAPPVIHDNHVVKVEHVDAPFEVERKKFVPKPFTVIKPVAVDFLKKITKVYDRKVEVPHFHPVPLIATIDKPRPVPFTREVVQHYDVHVNVPRPRPVKLTRHRTHVEQVPFDTPNVIVQNRPRHHEVREFVPKVSQGFNGRVFAG? |
| gi\|212831071 | *M.californianus* | gi\|391359391\| | Valine-rich protein / P. margaritifera | 34.25 | 0.013 | 217 | 2 | IVHIPIPK(34); VNQVVDLIHEVPMVVVK(77) | no domain; Val (16.8%), Pro (10.3%), Lys (9.3%) | ?LADXXARRIXXLGKXXCXXHTAXEXENXCXVVLFISLFAIGTLGHAPPPLQKKIVHIPIPKAVVRHKPLRQDRAHDVHQHVPLKRTEVRRVNQVVDLIHEVPMVVVKHKKFLSPVIIPKPIVVNEVLALPVIHDNHVVKVEHVDAPFEVERKKFVPKPFTVIKPVAVDFLKKITKVYDRKVEVPHFHPVPLIATIDKPRPVPFTREVVHIMTFT? |
| gi\|212821271 | *M.californianus* | gi\|391359391\| | Valine-rich protein/ P. margaritifera | 34.43 | 0.002 | 217 | 2 | IVHIPIPK(34); VNQVVDLIHEVPMVVVK(77) | Low complexity region | MLPVVLFISLFAIGTLGHVPPPLQKKIVHIPIPKAVVRHKPLRQDRAHDVHQHVPLKRTEVRRVNQVVDLIHEVPMVVVKHKKFLSPVIIPKPIVVNEVLALPVIHDKHVVKVEHVDAPFEVERKKFVPKPFTVIKPVAVDFLKKITKVYDRKVEVPHFHPVPLIATIDKPRPVPFTREVVQHYDVHVNVPRPRPVKLTRHRTHVEQVPFDTPNVIVQNRPRHHE? |
| gi\|212821058 | *M.californianus* | gi\|391359391\| | Valine-rich protein/ P. margaritifera | 33.7 | 0.009 | 217 | 2 | IVHIPIPK(34); VNQVVDLIHEVPMVVVK(77) | Low complexity region | MLPVVLFISLFAIGTLGHAPPPLQKKIVHIPIPKAVVRHKPLRQDRAHDIHQHVPLKRTEVRRVNQVVDLIHEVPMVVVKHKKFLSPVIIPKPIVVNEVLALPVIHDNHVVKVEHVDAPFEVERKKFVPKPFTVIKPVAVDFLKKITKVYDRKVEVPHFHPVPLITTIDKPRPVPFTREVVQ? |
| gi\|212818315 | *M.californianus* | gi\|391359391\| | Valine-rich protein/ P. margaritifera | 36 | 4.5 | 217 | 2 | IVHIPIPK(34); VNQVVDLIHEVPMVVVK(77) | Low complexity region | MLPVVLFISLFAIGTLGHAPPPLQKKIVHIPIPKAVVRHKPLRQDRAHDVHQHVPLKRTEVRRVNQVVDLIHEVPMVVVKHKKFLSPVIIPKPIVVNEVLALPVIHDNHVVKVEHVDAPFEVERKKFVPKTFTVIKPVAVDFLKEITKVLRQKKLRSHTFHPVPIDCPPLNKPRPITLSPEKSCFNHYDRSRLMVSKTQEPSSNLTKNPETSCLKQG? |
| gi\|212828530 | *M.californianus* | gi\|391359391\| | Valine-rich protein/ P. margaritifera | 35.78 | 1.00E-06 | 217 | 2 | IVHIPIPK(34); VNQVVDLIHEVPMVVVK(77) | Low complexity region | MLPVVLFISLFAIGTLGHAPPPLQKKIVHIPIPKAVVRHKPLRQDRAHDVHQHVPLKRTEVRRVNQVVDLIHEVPMVVVKHKKFLSPVIIPKPIVVNEVLALPVIHDNHVVKVEHVDAPFEVERKKFVPKPFAVIKPVAVDFLKKITKVYDRKVEVPHFHPVPLIATIDKPRPVPFTREVVQHYDVHVNVPRPRPVKLTRHRTHVEQVPFDTPNVIVQNRPRHHEVREF? |
| gi\|223026184 | *M.galloprovincialis* | gi\|14422379\| | calponin-like protein / M. galloprovincialis | 98.19 | 4.00E-157 | 215 | 4 | GMTGFGAVR(35);TDLKPSSSELPFK(44); HNYQGPTIGSKPTEK(37); YGVPNTSLFQTVELYEAR(96) | Calponin domain(PF00402) | MKKVIEGTPSESLPAACSKTDLKPSSSELPFKQMENIEKFLKAAHKYGVPNTSLFQTVELYEARNLPMVLATISHVGTEAQRHNYQGPTIGSKPTEKHIVQFSYEQLKQSHGTIGLQSGTNKFATQKGMRIGSVRHISDIKVEDLDKEGNTLLALQAGTNRFASQKGMTGFGAVRHIADIRADEFDKDGENIITLQAGTNKFAS? |
| gi\|223025178 | *M.galloprovincialis* | — | — | — | — | 200 | 2 | MMMMMPEMGGK(35); GMGMPMMMPETGGK(60) | Met (44.1%), Gly (27.6%) | ?MMMMPEMGGKGMGMMEKGMGMPMMMPEMGGKGMGMMEKMMMMMPEMGGKGMGMMEKGMGMPMMMPETGGKGMGMMEGGMGGKDMGMMMMPKMEMMGGKGMGMMEMPMSMPMMKGGMPMPMMEGGMGG? |
| gi\|212823360 | *M.californianus* | gi\|14422379\| | calponin-like protein / M. galloprovincialis | 96.06 | 6.00E-140 | 199 | 3 | HNYQGPTIGSKPTEK(37); NLPMVLATISHVGTEAQR(62); YGVPNTSLFQTVELYEAR(96) | Calponin homology domain ();Calponin domain(PF00402) | MADRVKPMGMDRALISKMGAKYDSGLEYEVRGWIKQLIGEDIGEGPSNVEKSLRDGVILCNLMKKVIDGTPSESLPAACAKTDLKSSPSELPFKQMENIEKFLKAAHKYGVPNTSLFQTVELYEARNLPMVLATISHVGTEAQRHNYQGPTIGSKPTEKHQVQFSYEQLKQSHGTIGLQSGTNKFATQKGMRIGSIRHISDIK? |
| gi\|145893257 | *M.californianus* | — | — | — | — | 186 | 2 | AQSLIDEAEQR(69); KAQSLIDEAEQR(98) | No domains; Arg (10.6%), Lys (9.6%), Ser (9.6%) | ?LKKRRQVPSSRIPVIPMNPDLQGMLKRRRRSNITWNNIRKAQSLIDEAEQRANMGKRTCKPYADHDPCCFTGGNQSSQGLNSYFKKKDFIESSF* |
| gi\|212816333 | *M.californianus* | gi\|14422379\| | calponin-like protein / M. galloprovincialis | 89.77 | 4.00E-108 | 184 | 2 | NLPMVLATISHVGTEAQR(62); YGVPNTSLFQTVELYEAR(96) | Calponin domain(PF00402) | MADRVKPMGMDRALISKMGAKYDSGLEYEVRGWIKQLIGEDIGEGPSNVEKSLRDGVILCNLMKKVIDGTPSESLPAACAKTDLKSSPSELPFKQMENIEKFLKAAHKYGVPNTSLFQTVELYEARNLPMVLATISHVGTEAQRHHFNGMTIGKKPTEKHIVNFSDEQLKAGQGII? |
| gi\|212814945 | *M.californianus* | gi\|14422379\| | calponin-like protein / M. galloprovincialis | 91.26 | 8.00E-115 | 184 | 2 | NLPMVLATISHVGTEAQR(62); YGVPNTSLFQTVELYEAR(96) | Calponin domain(PF00402) | MADRVKPMGMDRALISKMGAKYDSGLEYEVRGWIKQLIGEDIGEGPSNVEKSLRDGAILCNLMKKVIDGTPSESLPAACAKTDLKSSPSELPFKQMENIEKFLKAAHKYGVPNTSLFQTVELYEARNLPMVLATISHVGTEAQRLNFNGETIGSKPTVKHDVNFSYEQLKQSCGLIGLQSGTN? |
| gi\|223021924 | *M.galloprovincialis* | — | — | — | — | 177 | 3 | MMMMMPEMGGK(35); GMGMPMMMPEMGGK(49); GMGMPMMMMMPEMGGK(38) | Low complexity region; Met (42.3%), Gly (28.2%),Lys (10.6%) | ?WKKMMMMMPEMGGKGMGMMMPEMGGKGMGMMMPEMGGKGMGMMMPEMGGKGMGVMMPEMGGKGMGMMMPEMGGKGMGMMMPEMGGKGMGMMEKGMGMPMMMPEMGGKGMGMMEKMMMMMPEMGGKGMGMMKKGMGMPIMMPE? |
| gi\|223026183 | *M.galloprovincialis* | gi\|14422379\| | calponin-like protein / M. galloprovincialis | 96.11 | 2.00E-179 | 165 | 2 | HNYQGPTIGSKPTEK(37); YGVPNTSLFQTVELYEAR(96) | Calponin domain(PF00402) | MVLATISHVGTEVQRHNYQGPTIGSKPTEKHRVQFSYEQPKQSHGTIGLQSGTNKFATQKGMRIGSVRHISDIKVEDLDREGNTPLTLQAGTNRFASQKGMTGFGSVRHIADIRADQMDKEGENIITLQAGTNKFASQKGMTGFGAVRHVSDIRADDCDPQTSLHIGLQAGSNQFASQKGMTSMGAVRHICDIRADDLDREAQSVIPLQYGTNRGSNQKGMTSFGS? |
| gi\|58307710 | *M.galloprovincialis* | gi\|21105303\| | precollagen-D / M. galloprovincialis | 91.3 | 7.00E-15 | 153 | 3 | GSQGAVGPR(50); GETGPDGNQGQR(44); GSVGDQGAQGDQGATGADGK(103) | Collagen domain (PF01391) | ?FAPGPKGSVGDQGAQGDQGATGADGKKGEPGERGQQGAAGPVGRPGPRGDRGAKGIQGSRGRPGGMGRRGNRGSQGAVGPRGETGPDGNQGQRGEQGAPGVITLVIEDLRTAGVESPD* |
| gi\|58307802 | *M.galloprovincialis* | gi\|405967947\| | Transgelin-2 / C. gigas | 63.35 | 1.00E-64 | 153 | 3 | SPVNFQK(39); LINILLK(38); VMSPFVAMTNIENFNK(85) | Calponin homology domain ();Calponin domain(PF00402) | MSGSGRANKSGLGYEVEKKMEANYDREEAAGTPTHVVNWVNAILGSEHDPIPGTDWKSICNHLRDGVALCKLINILLKKDGKSPVNFQKKVMSPFVAMTNIENFNKGIQDYGVDRESEFQSGDLWEVRKGPFLNVINCISSLGFVANKKGVTPKYTGEIRKYLDNE* |
| gi\|238645227 | *M.galloprovincialis* | gi\|325504479\| | putative C1q domain containing protein MgC1q90 / M. galloprovincialis | 100 | 9.00E-132 | 150 | 2 | DGFIDLNDLR(60); EAPGPLNFTMFLSLFSEK(71) | Complement component C1q domain (SM000110) | MPSQCSTMQVICQTSPPTLTDVRKDLLDLDSYVDNKLRAIDTKIQQTACYNQGHTIVTFLARLVTPTHGTIAAKATLKFEKTTENVGNGYGNKTGIFTAPVKGLYHFTASARQSRSGYLHLGLYRNDEEMAVSVGVNYNSLTIGATFTLQYGDHVLVKNIWTQSSGIVGAGQSYFSGHLVHVM* |
| gi\|145890922 | *M.californianus* | gi\|317376180\| | Gigasin-2 / C. gigas | 28.63 | 2.00E-21 | 143 | 1 | TVTPDKVTYSIDVAVSEK(93) | Epidermal growth factor-like domain (SM000181) | MNILTKLTVFLLMCSVTAKYNCKMDGCQNGGNCTYFGNCECTHGFVGYDCGVQMALRSFGPSCSIKCMNGGTCYNTNMCYCTESFHGNRCQYQKENVQCGLEGIQITIMTSTKFQGEITTKPNSTGCQFRSFLTSNSTSLRSFRLITPLSLQTTNPCHRAINRTVTPDKVTYSIDVAVSEKEGVYNPLRDRIVNFKCVYPRRIGEGTTQDNTQSYTPVELLYLNTRLQPLTQEFKKGMSSL* |
| gi\|223023579 | *M.galloprovincialis* | — | — | — | — | 139 | 2 | FGGGFAGQVR(58); TGFGGVGSGFSGQFGGGFTGSAGR(108) | Transmembrane region; Gly (39.1%),Phe (13.7%), Ser (12.4%) | MYYVSALQQAYYTSSLRAGFGGFGSMIGGGLGGGFGSGFSGQFGGGFSGLAGRTGFGGVGSGFSGQFGGGFTGSAGRTGFGGVGSGFSGQFGGGFTGSAGRTGFGGVGNGFASRFGGGFAGQVRRFGVGTGGLTGSLGGGISGGFGSAFARRTSGFGSSGL? |
| gi\|223022206 | *M.galloprovincialis* | — | — | — | — | 110 | 2 | MMMMMPEMGGK(35); GMGMPMMMPEMGGK(49) | Met (44.1%), Gly (26.6%) | ?GMPMMMPEMGGKGMGMMEKMMMMMPEMGGKGMGMIEKGMGMPMMMPEMGGKGMGMM? |
| gi\|37650124 | *M.galloprovincialis* | gi\|405963175\| | 60 kDa neurofilament protein / C. gigas | 87.97 | 2.00E-72 | 107 | 2 | DIQSEYDNKVDQLR(75); IDLNNETLNHLDAENRR(48) | Filament domain (PF00038) | MEFLKKVHEQELKELAALAYRDTTEENREFWKSELSQAIRDIQSEYDNKVDQLRGDMESYYNLKVQEFRTGATKQNMEVTHVKEENKKLVK? |
| gi\|238643267 | *M.galloprovincialis* | gi\|405966499\| | Cathepsin L / C. gigas | 72.17 | 9.00E-120 | 106 | 3 | SDVGATDTGFTDVR(80); LIWEDNVAHIQQHNLAADR(32); TAGSTFLAPSNIGDLPDTVDWR(45) | Papain family cysteine protease Pept_C1 domain (SM000645) | MATDEFISIMNGYKMSINRTAGSTFLAPSNIGDLPDTVDWREKGYVTPIKNQGQCDSCWSFSATGSLEGQNFKKTGKLTSLSEQNLMDCSTAEGDHGCKGGLMDNAFRYIEKNKGIDTEMSYPYEAKNGMCRFKRSDVGATDTGFTDVRRGSEEELQQAVATVGPISVAIDAGHK? |
| gi\|238643266 | *M.galloprovincialis* | gi\|405966499\| | Cathepsin L / C. gigas | 77.44 | 3.00E-155 | 106 | 3 | SDVGATDTGFTDVR(80); LIWEDNVAHIQQHNLAADR(32); TAGSTFLAPSNIGDLPDTVDWR(45) | Papain family cysteine protease Pept_C1 domain (SM000645) | MATDEFISIMNGYKMSVNRTAGSTFLAPSNIGDLPDTVDWREKGYVTPIKNQGHCGSCWSFSATGSLEGQNFKKTGKLTSLSEQNLMDCSTAEGDHGCKGGLMDNAFRYIEKNKGIDTEMSYPYEAKNGMCRFKRSDVGATDTGFTDVRRGSEEELQQAVATVGPISVAIDAGHKSFQLYKTGVYSEPMCSSTRLDHGVLAVGYGTNMGQDYWLVKNSWGTKWGMEGYVM? |
| gi\|223024344 | *M.galloprovincialis* | — | — | — | — | 101 | 2 | GMGMMMPEMGGK(32); GMGMPMMMPEMGGK(49) | Met (44.1%), Gly (26.6%) | ?MPMMMPEMGGKGKGMPMMMPEMGGKGMGMPMMMPEMGGKGMGMMMPEMGGKGMGMMEGGMGG? |
| gi\|212821329 | *M.californianus* | gi\|405976801\| | Sodium-coupled monocarboxylate transporter 1 / C. gigas | 45.86 | 2.00E-34 | 99 | 2 | ELDATNLQTLNR(70); IVGDELSTAHAVMK(61) | Low complexity region; Val (9.9%), Ser (9.4%), Thr (7.5%) | MNIVYFTLILPFSMVSGIPTTPPNLKNNSLQSSLKELDATNLQTLNRETRKALLKIVGDELSTAHAVMKSIEPEVNDCQQKVDQKYYDCVQCVDMKCQNRYKECNGSIQISAPNSGGVSVSNSVNDRGRPTTTICTVMWGQSKACTTVMNPEGAFATSIKSMGDTIHVHLRSVVNDFGGSAGMFVNQMHSVVGDMNTSRVANEAVERMLNEL? |
| gi\|238643268 | *M.galloprovincialis* | gi\|405966500\| | Cathepsin L / C. gigas | 76.81 | 6.00E-117 | 98 | 2 | SDVGATDTGFTDVR(80); TAGSTFLAPSNIGDLPDTVDWR(45) | Papain family cysteine protease(SM000645) | MSVNRTAGSTFLAPSNIGDLPDTVDWREKGYVTPIKNQGHCGSCWSFSATGSLEGQNFKKTGKLTSLSEQNLMDCSTAEGDHGCKGELMDNAFRYIEKNKGIDTEMSYPYEAKNGMCRFKRSDVGATDTGFTDVRRGSEEELQQAVATVGPISVAIDAGHKSFQLYKTGVYSEPMCSSTRLDHGVLAVGYGTNMGQDYWLVKNSWGTK? |
| gi\|145887968 | *M.californianus* | gi\|322966920\| | Shell matrix protein / M. californianus | 100 | 2.00E-171 | 93 | 2 | TGGNLEIR(69);GGLAFDYSHISLR(58) | Laminin_G_3 domain (PF13385) | MNGNGGLRGSARKQFRQCSAEFKINFDDGFKDISKGGLAFDYSHISLRRGKGVFVGNSKLYIWGFQSRFLGKTFAIRMKVKIKRGAGKYRPEPIISNCGPNGDSSVEIVVHRGKVIFKAKTSDNPEAVFITEDYDDDKWTDLTYYYDGNHFGGSCNGRPFRQRTGGNLEIRDNPMTIGLCTGQNGFHGEIDELEIYTACIPKDM* |
| gi\|145895570 | *M.californianus* | gi\|405966499\| | Cathepsin L / C. gigas | 72.76 | 4.00E-135 | 90 | 2 | SDVGATDTGFTDVR(80); LIWEDNVAHIQQHNLAADR(32) | Cathepsin propeptide inhibitor domain(SM000848);Papain family cysteine protease(SM000645) | MKLLVLVLCVGVALSKPLNQELDQEWQLYKNTYNKNYDVHEHILRRLIWEDNVAHIQQHNLAADRGEHTYWLGTNEYADMATDEFISIMNGYKMSINRTAGSTFLPPSNIGDLPDTVDWREKGYVTPIKNQEQCGSCWSFSATGSLEGQNFKKTGKLTSLSEQNLVDCSTAEGDHGCKGGLMDNAFRYIEKNKGIDTEMSYPYEAKNGMCRFKRSDVGATDTGFTDVRRGSEEELQQAVATVGPISVAIDAGHKSFQLYKTGVYSEPMCSS? |
| gi\|238641522 | *M.galloprovincialis* | gi\|405971603\| | Cathepsin L / C. gigas | 53.15 | 7.00E-75 | 89 | 3 | YFDIQPASVK(52); SYHTIEEETYRR(23); FLPPLNAVLPENVDWR(51) | Cathepsin propeptide inhibitor domain(SM000848);Papain family cysteine protease(SM000645) | MLLLCILLVAIATVFTAPQTQDQNSIIRYFDIQPASVKMTVTAPKGHVVTSFEPYDKSWEKFKLEHSKSYHTIEEETYRRTVFKKNALKIEEHNKQYSLGQKSYYLGINQFADLEHWEYMQHHGFQVKKTVNRTRTGSKFLPPLNAVLPENVDWRDKGYVTPVKNQGQCGSCWSFSTTGALEGQHFRKSGKLLSLSEQQLVDCSGDYGNEGCNGGLMDDAFKYI? |
| gi\|223027775 | *M.galloprovincialis* | — | — | — | — | 81 | 2 | TDSSHFGLAR(33); TAMDLYGDGQGYGGR(81) | Low complexity region; Gly 19.3%), Tyr (16.0%), Asp (11.6%), Lys (11.6%) | GRTWLALFVVATTFALSCADYSNVGQYNSYKKKESYGSYPVYGGQSQGYNNNQYYGSQSQSYNNDYDYGKNIYNKKDYGKKDNDYGKKDYGKKDYGKKDYGYDKKDSNRGGRRGGRTDSSHFGLARDLVILKTAMDLYGDGQGYGGRGGYRGGRDGYGSGGYGGYDNKGKKDYGYDDYKDY? |
| gi\|212816824 | *M.californianus* | gi\|405950795\| | Non-neuronal cytoplasmic intermediate filament protein / C. gigas | 58.78 | 5.00E-80 | 80 | 2 | ALLAEIDR(48); LNDQLGNNEGELANLR(61) | Filament domain (PF00038) | SRVVRSGRKAFGCAVGFKSRLVEKFKVSQEKVEIRRNIKTQPTIGTRSTVINRTSHGGGSIMPGGGSRSVSMRMSMGGSAPSFAQGTVSSMSHKNVANVLDTRAKEKTEMNVLNERFASYIEKVRFVEAQNKALLAEIDRLKKQKNFDASEIKELYEQEIADSRKIIDDLSDEKAKFDATLVSLQDQLEDERRDRINAEKTVDDLSNKIDRLNDQLGNNEGELANLRLRIETLEDENARLKKDKRTLXDDIGRIRADLDEETCKRIQAEMK? |
| gi\|58307533 | *M.galloprovincialis* | — | — | — | — | 80 | 3 | SSSSTNEMTVR(49); ALVDTETYVSPR(40); SALYEDTFIPEVIRPR(35) | Ser (14.2%), Arg (13.0%), Val (8.0%), Ala (8.0%) | MTVRRSRYSSVPPGYFASTKGHSALKRWSYAPQSRSALYEDTFIPEVIRPRSYYDTSREENDIRRGVNDELVYTSNLMDDTYDVAAKSRSRDQMLLRDAARALVDTETYVSPRSSVTSNRVRATSVVARPAPLTSRAVSCPPTSRRSNQPLYGGKSHWDEEG? |
